# Transmission dynamics of the 2016-18 outbreak of hepatitis A among men who have sex with men in England and cost-effectiveness analysis of vaccination strategies to prevent future outbreaks

**DOI:** 10.1016/j.lanepe.2022.100426

**Published:** 2022-06-17

**Authors:** Xu-Sheng Zhang, Jason J. Ong, Louis Macgregor, Tatiana G. Vilaplana, Simone T. Heathcock, Miranda Mindlin, Peter Weatherburn, Ford Hickson, Michael Edelstein, Sema Mandal, Peter Vickerman

**Affiliations:** a Statistics, Modelling and Economics, Data, Analytics & Surveillance, UK Health Security Agency, UK; b University of Bristol, Bristol, UK; c Monash University, Melbourne, Australia; d Immunisation and Vaccine Preventable Diseases Division, UK Health Security Agency, UK; e North East London Health Protection Team, UK Health Security Agency, UK; f South London Health Protection Team, UK Health Security Agency, UK; g London School of Hygiene & Tropical Medicine, UK

**Keywords:** Hepatitis A virus, Immunisation, Men who have sex with men, Cost-effectiveness

## Abstract

**Background:**

Despite being vaccine-preventable, hepatitis A virus (HAV) outbreaks occur among men who have sex with men (MSM). We modelled the cost-effectiveness of vaccination strategies to prevent future outbreaks.

**Methods:**

A HAV transmission model was calibrated to HAV outbreak data for MSM in England over 2016-2018, producing estimates for the basic reproduction number (R_0_) and immunity levels (seroprevalence) post-outbreak. For a hypothetical outbreak in 2023 (same R_0_ and evolving immunity), the cost-effectiveness of pre-emptive (vaccination between outbreaks among MSM attending sexual health services (SHS)) and reactive (vaccination during outbreak among MSM attending SHS and primary care) vaccination strategies were modelled. Effectiveness in quality-adjusted life-years (QALYs) and costs were estimated (2017 UK pounds) from a societal perspective (10-year time horizon; 3% discount rate). The incremental cost-effectiveness ratio (ICER) was estimated.

**Findings:**

R_0_ for the 2016-2018 outbreak was 3·19 (95% credibility interval (95%CrI) 2·87-3·46); seroprevalence among MSM increased to 70·4% (95%CrI 67·3-72·8%) post-outbreak. For our hypothetical HAV outbreak in 2023, pre-emptively vaccinating MSM over the preceding five-years was cost-saving (compared to no vaccination) if the yearly vaccine coverage rate among MSM attending SHS was <9·1%. Reactive vaccination was also cost-saving compared to no vaccination, but was dominated by pre-emptive vaccination if the yearly vaccination rate was >8%. If the pre-emptive yearly vaccination rate fell below this threshold, it became cost-saving to add reactive vaccination to pre-emptive vaccination.

**Interpretation:**

Although highly transmissible, existing immunity limited the recent HAV outbreak among MSM in England. Pre-emptive vaccination between outbreaks, with reactive vaccination if indicated, is the best strategy for limiting future HAV outbreaks.

**Funding:**

NIHR.

**Research in context**

**Evidence before this study**

Hepatitis A outbreaks in high-income countries occur periodically, particularly among men who have sex with men (MSM) subpopulations. These outbreaks can be prolonged and cause substantial morbidity. The most effective way to control these outbreaks is through immunisation. We did a search on PubMed on 25^th^ Jan 2022 for economic evaluations of hepatitis A vaccinations using the terms “Hepatitis A” AND “economic evaluation” OR “Cost-effectiveness” OR “Cost-benefit”. We found several economic evaluations for childhood vaccination and for travellers demonstrating mixed results regarding its cost-effectiveness. Two studies evaluated Hepatitis A vaccination in the US. One study reported that Hepatitis A and B vaccination among individuals attending STI clinics could be cost-effective, although they did not distinguish MSM from non-MSM, and another study reported that Hepatitis A vaccination would be cost-effective among MSM in USA. Currently there is no consensus on the best Hepatitis A vaccination strategy to prevent future outbreaks among MSM.

**Added value of this study**

Using data from the most recent Hepatitis A outbreak in the United Kingdom (2016-2018), we created a transmission dynamic model to examine the cost-effectiveness of various Hepatitis A vaccination strategies. We show that reactive vaccination of MSM during future outbreaks can be a cost-saving strategy for substantially reducing its magnitude. However, pre-emptively vaccinating MSM between outbreaks in sexual health services can save more money and have more impact if the pre-emptive vaccination rate is sufficiently high (9% vaccination rate per year among MSM attending Sexual Health Services if done for the 5 years preceding an outbreak), suggesting that this should be the best first choice.

**Implications of all the available evidence**

Maintaining sufficient background immunity (at least 70%) can prevent large Hepatitis A outbreaks. This immunity is best maintained through a pre-emptive vaccination strategy occurring between outbreaks, and then reactive vaccination strategies if the pre-emptive vaccination coverage is insufficient to prevent propagation of an outbreak.

Alt-text: Unlabelled box

## Introduction

Hepatitis A virus (HAV) is transmitted through the faecal-oral route, with most adults experiencing symptomatic disease. Although most individuals clear the infection with resultant lifelong immunity, many require hospitalisation with death from fulminant hepatitis being a rare outcome.^1^^,^^2^ Effective vaccines can prevent HAV infection.

In high-income countries (HIC) where the risk of HAV infection from contaminated food or water is low, periodic outbreaks of HAV occur among men who have sex with men (MSM).^3^ These outbreaks can be prolonged and cause substantial morbidity. The most effective way to control these outbreaks is through immunisation. However, many HIC do not have targeted vaccination programmes because of concerns that it may not be cost-effective, particularly if they already provide universal childhood vaccination for HAV.^4^ In the UK there is no universal childhood vaccination, but selective vaccination is recommended for those at risk, such as travellers to endemic countries and MSM.^5^

Following recent outbreaks of HAV among MSM,^6^^,^^7^ HAV vaccination has been recommended for MSM attending sexual health services (SHS) across England. However, implementation of this recommendation has been variable^8^ and uptake has not been routinely monitored. As a result, there was uncertainty around the susceptibility of MSM to HAV prior to the outbreak that mainly affected MSM across England in 2016-2018.^9^ This outbreak was initiated by importations of HAV among MSM returning from European countries.^9^ The main outbreak response was to increase HAV vaccination among MSM. Public Health England (PHE) - now UK Health Security Agency (UKHSA) - recommended that all MSM attending SHS should have at least one dose of HAV vaccine. However, immunisation was initially limited by vaccine supply constraints and local commissioning challenges. PHE also advised that contacts of cases should be vaccinated in primary care.^9^ The direct healthcare costs of the outbreak were estimated to be £1·5 million, primarily driven by hospitalisations.^9^ This estimation did not incorporate broader costs of the public health response, including health protection, awareness-raising, stakeholder engagement and incident response support.

Given this outbreak's health burden and costs, it is important to evaluate the cost-effectiveness of different HAV vaccination strategies for preventing future outbreaks. For England, we use an HAV transmission model to compare the cost-effectiveness of either vaccinating MSM before an outbreak occurs (pre-emptive vaccination) or only vaccinating MSM once an outbreak has started (reactive vaccination).

## Methods

In this paper, we firstly develop and use a HAV outbreak model with data from the 2016/18 outbreak (and other sources) to estimate the transmission potential and existing levels of immunity for HAV among MSM in England. This model is then adapted to simulate a hypothetical outbreak in 2023 to assess the impact and cost-effectiveness of different vaccine strategies to prevent it, either reactive, based on levels of vaccination achieved in the 2016/18 outbreak, or a hypothetical pre-emptive vaccination scenario with different yearly rates of vaccination.

### Outbreak model description

**Figure 1:**
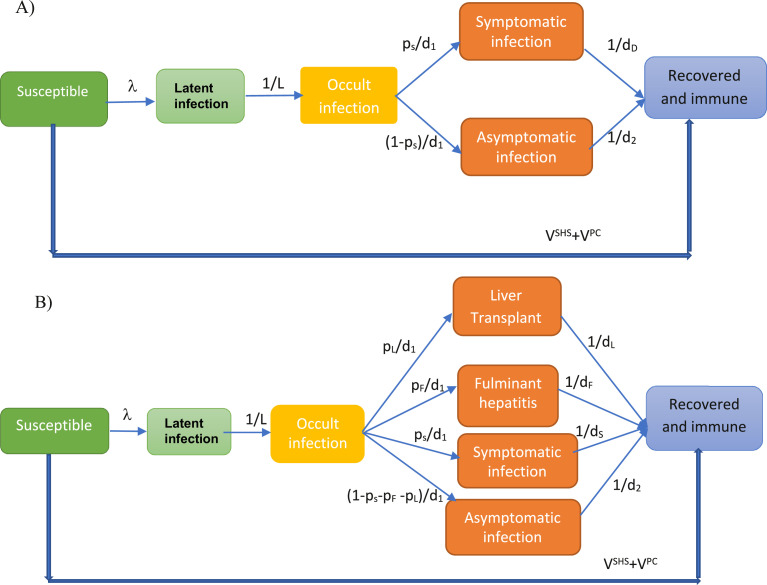
Schematic of the model of hepatitis A virus transmission among men who have sex with men for model fitting to the 2016/18 outbreak (A) and for assessing the impact and cost-effectiveness of different vaccination strategies for the hypothetical outbreak in 2023 (B). The thick blue line denotes the effect of vaccination. Within the transmission model for the 2016/18 outbreak (panel A), individuals start as *susceptible* to infection; under the force of infection *λ*, they are exposed to HAV. When *exposed* they become latently infected but not yet infectious, after a latent period of *L*, they then become *occultly* infectious, with a proportion (*p*_s_) of individuals after an infectious period *d*_1_ then becoming *symptomatic* and the rest remaining *asymptomatic* but infectious. They then *recover* after *d*_D_ days for symptomatic and *d*_2_ days for asymptomatic individuals and become immune to HAV. For the model assessing vaccination strategies (panel B), among *occultly* infectious individuals, there is a proportion *p*_F_ developing fulminant hepatitis and a proportion *p*_L_ requiring liver transplant in addition to those becoming symptomatic and asymptomatic individuals.

Figure 1 shows the schematic for our dynamic model of HAV transmission among MSM (further details in SI.1). The model includes six infection states. Individuals start as susceptible to infection. When exposed, they become latently infected but not yet infectious. They then become occultly infectious, with individuals either becoming symptomatic or remaining asymptomatic but infectious. A proportion of symptomatic cases are admitted to hospital. Individuals recover and become immune to HAV. The rare possibility of developing fulminant hepatitis is not included in this model for the 2016/18 outbreak.

The rate of infection is proportional to the number currently infectious and the rate of sexual contacts among MSM. However, we assume that symptomatic men are too sick to have sexual contacts^10^ and so are not infectious, but relax this assumption in the sensitivity analysis. Occult and asymptomatic infections are assumed to have the same transmissibility.

The model stratifies MSM into low and high sexual risk groups (defined by the frequency of new sexual partners among MSM in the last year) and by whether they attend SHS or not. Rates of sexual contact across these four groups are defined by a contact matrix that determines their infection rate.

The outbreak responses of immunising (i) all MSM attending SHS and (ii) close contacts of HAV cases in primary care were defined as two vaccination rates in the model. However, because HAV immune status is generally unknown, these vaccinations only protect MSM susceptible to HAV, with individuals assumed to be protected immediately after vaccination.^5^^,^^11^ As the 2016/18 outbreak was about two years, we ignored inflows and outflows of MSM when modelling the outbreak.

### Parameterisation for the 2016/18 outbreak

Previous studies were used to obtain fixed values for HAV natural history parameters and uncertainty distributions for other model parameters (Table 1). This included the duration of different disease stages. We used the occurrence of jaundice to categorise symptomatic infection, with data^14^ suggesting 84·1% of infected adults experience jaundice. European data suggests a wide range for susceptibility to HAV,^17^ so we assumed 20-70% for all MSM sub-groups.

**Table 1:** Prior and posterior distributions for parameters of the transmission model for 2016/18 outbreak.

Parameters (to be estimated)	Prior value or range	Posterior -median and [95% CrI (credible interval)]
Transmission coefficient (*β*) per week	U[0·4,2·6]	1·48 [1·33,1·60]
Initial seroprevalence (1-*ρ*) before the outbreak	U[0·2,0·7]	0·691 [0·659,0·716]
Seroprevalence post outbreak	–	0·704 [0·673,0·728]
Increase in seroprevalence due to outbreak	–	0·013 [0·012,0·014]
Reduction in contact rate (1-*w*) due to public health response	U[0·0,0·5]	0·169 [0·131,0·214]
Time point when reduction in contact rate occurs in weeks after the start of the outbreak (*t_c_*)	U[98/4,98]*	48·7 [43·1,54·1]
Basic reproduction number *R*_0_ before behavioural response	–	3·19 [2·87,3·46]
Effective reproduction number *R*_e_ after behavioural response	–	0·82 [0·77,0·85]
**Parameters (fixed)**	**value**	**Reference**
Number of MSM in England	531,559	^12^
Latent period duration (*L*)	10·0 days	^10^^,^^13^
Occult infection period duration (*d*_1_)	14·0 days	
Asymptomatic infection period duration (*d*_2_)	7·0 days	
Proportion of infections that become symptomatic amongst adults (aged 15 to 49 years) (*p*_S_)	84·1%	^14^
Probability of hospitalization for symptomatic cases (*ϕ*_H_)	57·0%	^2^
Time from symptom onset to hospitalisation (*d*_H_)·	2·0 days	
Infectious period duration of symptomatic cases (*d*_D_)	2·9 days	1/(*ϕ*_H_ /*d*_H_+(1-*ϕ*_H_)/*d*_2_)
Duration of immunity induced by one dose of vaccination	7 years	^15^^,^^16^
Duration of immunity induced by two doses of vaccination	25 years	
Vaccine efficacy against becoming infected	100%	^5^

U denotes uniform distribution· * 98 is the outbreak duration in weeks (July 2016 to May 2018).

Natural HAV infection confers lifelong immunity. However, the duration of immunity after vaccination is 5-7 years after one dose and about 25 years after two doses.^15^^,^^16^ We assumed that immunisation provides 100% efficacy against infection,^5^ with two doses effectively giving life-long protection. We used monthly data on vaccinations via SHS clinics, which started in July 2017 (Table S2), to parameterise the rate of reactive vaccination of MSM attending SHS, while the rate of vaccination of contacts in primary care used data on the number of vaccines done per case (SI.1.2). The estimated MSM population size for England was 531,559.^12^

Data from the European MSM Internet Survey (EMIS), which was undertaken in 22 European countries in 2010, including 18,000 MSM in the UK was used to define the sexual behaviour of MSM and the proportion attending SHS.^18^ The HAV transmission coefficient was estimated during model calibration. Additionally, because outbreak data suggests a downturn in the number of new cases after April 2017, we allowed a reduction in the contact rate after a specific date. Both parameters were estimated through model calibration (SI.1.3, SI.1.4).

### Calibration to 2016/18 outbreak

We used a Bayesian Markov Chain Monte Carlo (MCMC) framework to calibrate the model to data on the weekly number of cases during the outbreak and the seroprevalence post-outbreak. This included data from 725 male outbreak cases between 17 July 2016 to 30 May 2018, which were all assumed to be MSM despite fewer reported as such (n=507). For seroprevalence, pooled data from seven surveys estimated 65·8% (SD=15·0%) of MSM being antibody positive in England in 2018 (Table S3)(^19^^,^^20^ and PHE unpublished data). The calibration (see SI.1.4) involved varying four parameters which were estimated with uncertainty: (1) transmission coefficient; (2) proportion of MSM initially susceptible to infection; (3) reduction in contact rate during the HAV outbreak; and (4) time at which this reduction occurs.

### Vaccination strategies for future outbreaks

We considered the impact and cost-effectiveness of two vaccination strategies on a hypothetical modelled HAV outbreak in 2023: **pre-emptive vaccination** before the outbreak and **reactive vaccination** during the outbreak.

#### Pre-emptive vaccination (see SI.2.1)

We simulated different hypothetical levels of pre-emptive vaccination for SHS attendees during a 5-year non-outbreak period of 2018-2023. Although initial testing for immunity was considered, the cheapest strategy involved vaccinating without testing and so is considered here. We assumed a 50% return rate for second vaccination visits based on variable return rates (30-95%) achieved for other vaccines.^21^^,^^22^ We estimated the vaccinations required to achieve different increases in immunity/seroprevalence amongst SHS attendees over 2018-2023, which were then used in our model to simulate their effect on the modelled HAV outbreak.

#### Reactive vaccination (see SI.2.2)

We assumed that during the HAV outbreak, pre-emptive vaccination would be replaced with reactive vaccination initiatives in SHS and primary care. Reactive vaccination is assumed to be responsive to the ongoing number of cases, based on the responsive vaccination rate during the 2016/18 outbreak and starting when the outbreak was signalled. We assumed the daily number of vaccinations depended on the previous days’ number of cases, with the vaccines per case being 34·41 via SHS clinics and 3·31 via primary care.

For each vaccination scenario, we estimated its impact on the hypothetical HAV outbreak initiated in 2023 with 39 imported cases over 8 months (similar to 2016-18 outbreak). The model assumed the median basic reproductive rate (*R_0_*) for the 2016-18 outbreak, no reduction in contact rate during the outbreak, and the pooled seroprevalence/immunity estimate (65·8%; Table S3) following the end of the 2016/18 outbreak. The adapted model also included inflow and outflow of individuals, and so the immunity level decreased over 2018-2023 without pre-emptive vaccination. Following discussions with PHE, it was decided that an outbreak would be signalled if the number of cases exceeded 30 in a 3-month window, with the start date being the end of this period. Outcomes for the outbreak were then calculated up to when the case rate decreased to ≤1 per month. No uncertainty was assumed in the hypothetical outbreak or the parameters used to model the vaccination scenarios.

### Estimating health benefits and costs

The outputs for each modelled outbreak in 2023 incorporating different vaccination scenarios were used to estimate the costs from a societal perspective for that vaccination strategy, public health response (PHR), health care costs, productivity losses, and quality-adjusted life years (QALY) gained. To make these costs and QALYs comparable across different vaccination scenarios, they were always estimated over 10 years, with costs reported in 2017 UK pounds (GB£) and a 3% annual discount rate applied to all costs and QALYs. A model adaptation was included (Figure 1B) to incorporate the possibility of fulminant hepatitis or liver transplant.^15^ No uncertainty was incorporated into these model projections.

### Health benefits

To estimate QALYs, we incorporated utility weights for asymptomatic (0·83), symptomatic (0·64), fulminant (0·26) and post-liver transplant (0·73) cases,^15^^,^^23^ with other health states assumed to have a utility weight of 0·90 (averaged over three age ranges from^15^). See the supplementary materials for more details. The total QALYs were calculated for the outbreak by applying these utility weights to individuals in each model category over time.

### Productivity losses

We estimated the productivity losses due to HAV. Estimates of the weekly wage for adult males (£573) and their employment rate (80·4%)^24^ for England in 2020 were applied to the estimated days of absenteeism^15^ for every symptomatic outpatient case (15·5), inpatient case (33·2; not liver transplant) and transplant patient (153·2). These wage costs were summed across all new symptomatic cases over time.

### Costs of pre-emptive vaccination

The pre-emptive vaccination scenarios involve costs for staff time and vaccines used, with these calculations assuming that 25% of the cost for the first SHS clinic visit was allotted to vaccination (SI.2.1).

### Costs of outbreak and reactive vaccination

Outbreaks of HAV incur costs for the PHR and healthcare management of cases. These costs were estimated from the 2016/18 outbreak, with the PHR including costs for reactive vaccination initiatives, staff time for incidents of different magnitude, preparing and coordinating meetings and developing health promotional materials. Cost estimates are in Table S6, with more details in SI.3.

### Cost-effectiveness of vaccination strategies

We estimated the incremental cost-effectiveness ratio (ICER)^23^ for different pre-emptive and reactive vaccination initiatives, and both strategies combined. We used the NICE recommended willingness-to-pay threshold of £20,000 per QALY gained to determine if an intervention was cost-effective.^25^

### Sensitivity analyses

We undertook sensitivity analyses to test the effect of different model assumptions or uncertain parameter values on the cost-effectiveness of pre-emptive and reactive vaccination strategies, solely or combined, on the 2023 modelled outbreak. This sometimes included changing assumptions for the modelled outbreak in 2016/2018, with resulting implications then being included in the 2023 outbreak model if they resulted in significant changes. For pre-emptive vaccination, we only considered vaccination scenarios that increased seroprevalence among MSM attending SHS by 7 or 9% in absolute terms over the 5-year non-outbreak period. We considered the effect of assuming: 1) 10-year gap between the new outbreak and the 2016/2018 outbreak (instead of 5 years); 2) 20% like-with-like mixing among low and high-risk MSM (instead of 3·5%); 3) mildly symptomatic cases (those not admitted to hospital; 20·5% of cases in 2016/2018 outbreak) continue to be sexually active (instead of not being sexually active); 4) SHS attendees have greater immunity instead of equal immunity among four MSM sub-groups; 5) only those cases that stated being MSM (507 instead of 725) were modelled in the 2016/2018 outbreak; 6) level of immunity among MSM remains steady after the 2016/18 outbreak (instead of decreasing); 7) outbreak response is triggered after 50 cases over 3 months for 2023 outbreak instead of 30 cases; 8) reactive vaccinations start 52 weeks after outbreak response is triggered (similar to 2016/18 outbreak) instead of immediately; 9) all reactive and pre-emptive vaccinations are targeted to high-risk MSM attending SHS instead of all MSM attending SHS; 10) include reduction in sexual contact rate in the 2023 outbreak as seemed to occur in the 2016/18 outbreak; 11) 5-year duration of immunity after one vaccine dose instead of 7 years; 12) 75% return rate for 2^nd^ vaccine dose instead of 50%; 13) reactive vaccination rate is twice the rate used in baseline; and 14) utility weights are decreased by 10% in absolute terms.

### Role of the funding source

The funders had no role in the study design, data collection, data analysis, interpretation, writing of the report.

## Results

### Transmission characteristics of the 2016/18 outbreak

**Figure 2:**
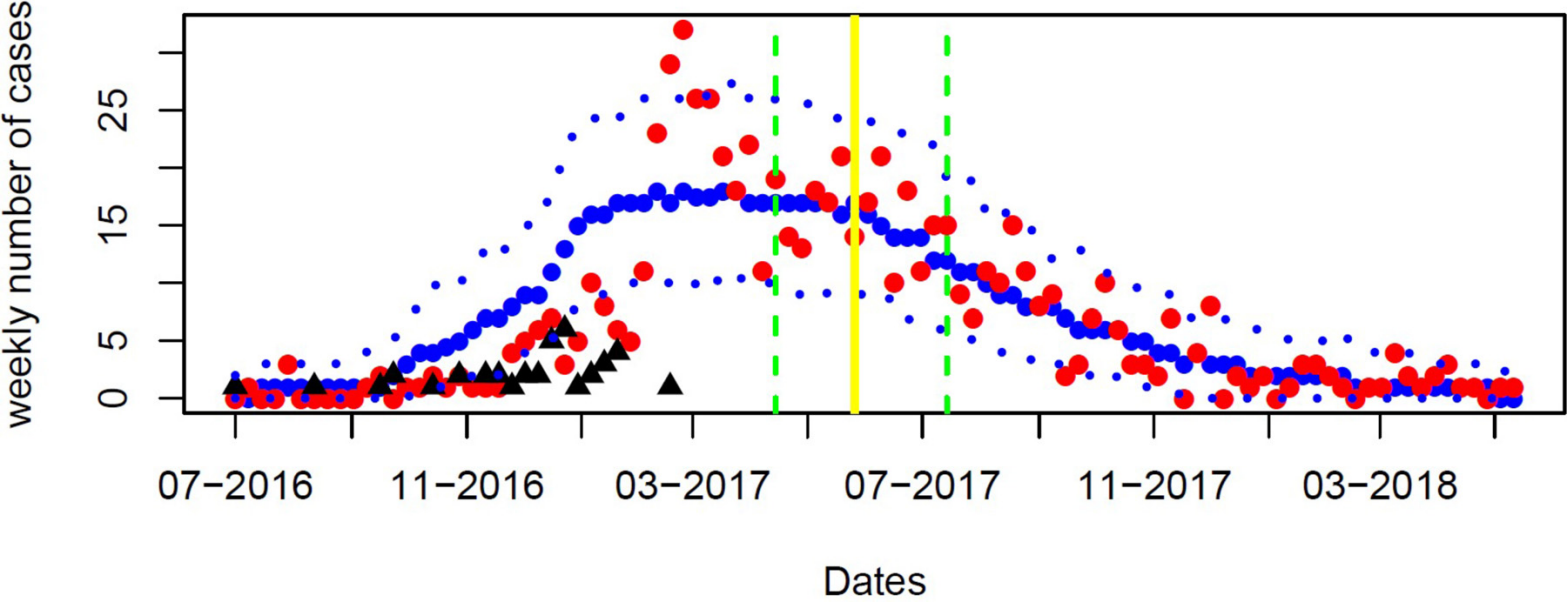
Model fitting to the 2016/18 hepatitis A outbreak data of 725-39=686 male cases (red dots for cases per week) with vaccination from July 2017 to July 2018. The black triangles represent the 39 imported cases. Thick blue dots represent the median and thin blue dots 95% credibility interval of the model projections. The yellow vertical line is the median of the estimated time when the model estimates the contact rate decreases, and the two green vertical lines represent the lower and upper of the 95% credibility interval.

Figure 2 shows the calibration of the model to the 2016/18 outbreak. The model estimates a *R*_0_ of 3·19 (95% credibility interval (95%CrI) 2·87-3·46), 740 (95%CrI: 336-1281) outbreak cases, and a 16·9% (95%CrI 13·1-21·4%) relative reduction in contact rate between May and July 2017 (months 10-12 of outbreak) due to the PHR (Table 1). The initial seroprevalence in July 2016 was 69·1% (95%CrI 65·9-71·6%), with this increasing to 70·4% (95%CrI 67·3-72·8%) following the outbreak, comparing reasonably with our data estimate (65·8%; 95%CI 54·9-76·9%). By the end of the outbreak, the effective reproduction number reduced to 0·82 (95%CrI 0·77-0·85).

The above scenario assumed reactive vaccinations occurred from week 50 of the outbreak. If these had not occurred, the model projects that 848 (95%CrI 396-1423) cases would have occurred. Conversely, if the MSM contact rate had not reduced, then 1160 (95%CrI 574-1906) cases would have occurred, indicating that the downturn in the outbreak was mainly due to this reduction. If these control measures had been initiated earlier, then the outbreak size would have reduced to 591 (95%CrI 257–1084) if reactive vaccinations had occurred from the start of outbreak and 368 (95%CrI 122–782) if the contact rate had reduced in the 4^th^ month.

#### Vaccination required for controlling outbreaks

**Figure 3:**
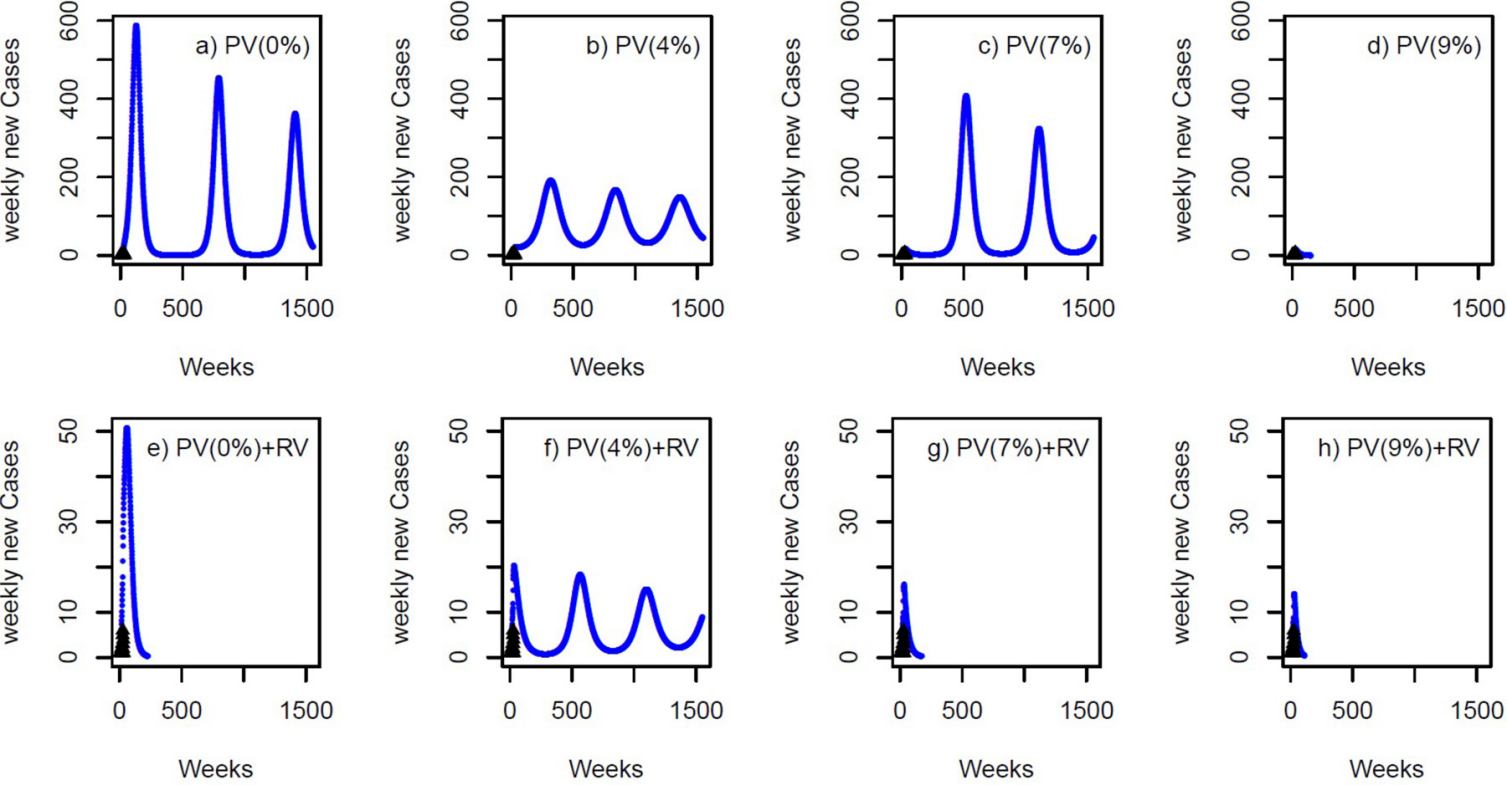
Modelled outbreaks from 2023 with either (a) no vaccination, or (b, c and d) just pre-emptive vaccination (PV=4%, 7% and 9% per year), or (e) just reactive vaccination (RV), or (f, g and h) PV with RV (PV(4%)+RV, PV(7%)+RV, PV(9%)+RV). The percentage in PV(%) represents the yearly rate of pre-emptive vaccination of MSM who attend SHS clinics. The outbreaks are generated by 39 imported cases (as occurred in the 2016/18 outbreak - black triangles). A 30-year time period is presented to show the periodic epidemics.

For our modelled outbreak in 2023, if no pre-emptive vaccination occurred over 2018-2023, the seroprevalence among MSM would reduce from 65·8% to 64·2% by 2023. Additionally, if there was no reactive vaccination or reduction in contact rate during the outbreak, then our hypothetical outbreak from 2023 becomes endemic and oscillates, resulting in 52,951 cases over 10 years (Figure 3, Figure S3, Table 2).

**Table 2:** Cost-effectiveness analysis under pre-emptive vaccination (PV) and reactive vaccination (RV) alone or in combination. No reduction in contact rate during the outbreak was assumed. Costs are in thousands of GB£ and ICER are in thousands of pounds per QALY.

Increase in seroprevalence among SHS attendees	Duration of outbreak (days)	Total cases during outbreak	Vaccination costs for PV only	Outbreak costs (includes RV costs)	Productivity losses	Total cost	QALYs	Incremental - comparing current scenario to previous scenario			Incremental - comparing current scenario to baseline scenario			Incremental - comparing the combination of RV and PV to PV alone with same coverage		
								Costs	QALYs	ICER	Costs	QALYs	ICER	Costs	QALYs	ICER
**Pre-emptive vaccination alone** without control measures taken during the outbreak, with the outbreak cost only including costs due to Clinical Case Management																

0(baseline)	3558^#^	52,961	0	110,978	8531	119,509	4,121,628									
0·01	3553^#^	44,109	1902	89,625	4,478	96,006	4,122,076	-23,503	448	CS	-23,503	448	CS			
0·05	3544^#^	41,785	5517	76,581	3,148	85,246	4,122,362	-10,759	286	CS	-34,262	734	CS			
0·06	3542^#^	40,673	6468	72,112	3,996	82,577	4,122,488	-2670	126	CS	-36,932	859	CS			
0·07	3540^#^	26,463	7610	45,880	4,948	58,437	4,123,111	-24,140	623	CS	-61,071	1483	CS			
0·08	3537^#^	3,825	8561	6904	1,082	16,547	4,123,818	-41,890	707	CS	-102,962	2190	CS			
0·09	1054	571	9702	1267	180	11,149	4,123,911	-5397	93	CS	-108,359	2283	CS			
0·1	797	464	10,844	1031	167	12,041	4,123,916	892	5	184	-107,467	2287	CS			

**Reactive vaccination alone or with pre-emptive vaccination**, with the outbreak cost including costs due to Clinical Case Management and Public Health Response																

RV alone	1597	3770	0	13,406	755	14,160	4,123,767				-105,348	2139	CS	-105,348	2138	CS
0·01+RV	3553^#^	2924	1902	10,341	486	12,729	4,123,808	-1431	41	CS	-106,779	2180	CS	-83,276	1732	CS
0·05+RV	3544^#^	2023	5517	6754	398	12,669	4,123,858	-60	49	CS	-106,840	2229	CS	-72,577	1495	CS
0·06+RV	3542^#^	1433	6468	4919	333	11,720	4,123,880	-949	22	CS	-107,788	2252	CS	-70,857	1392	CS
0·07+RV	1222	725	7610	2672	206	10,488	4,123,904	-1232	24	CS	-109,021	2276	CS	-47,949	793	CS
0·08+RV	956	588	8561	2175	189	10,925	4,123,910	437	6	71	-108,583	2282	CS	-5621	92	CS
0·09+RV	791	491	9702	1825	175	11,702	4,123,915	777	4	178	-107,807	2286	CS	553	4	153
0·1+RV	675	420	10,844	1565	163	12,571	4,123,918	869	3	268	-106,937	2289	CS	530	2	264

CS denotes cost saving where the option is cheaper compared to the comparator and QALYs are gained. **#** The outbreak is periodic (see Figure 3) for all these vaccine scenarios – they either undertake pre-emptive vaccination and increase the seroprevalence of MSM attending SHS by 1 to 8% (over 5 years) or they undertake reactive vaccination with pre-emptive vaccination and increase the seroprevalence of MSM who attend SHS by 1 to 6% (over 5 years). Estimates listed are for the first 10 years.

Without pre-emptive vaccination, the seroprevalence or immunity level reduces from 65·8% to 64·2% at the end of a 5-year period. The incoming outbreak is assumed to be induced by importation of infections as for the 2016/18 outbreak.

### Pre-emptive vaccination strategies

For this modelled outbreak initiating in 2023, our results in Table 2 show that incorporating pre-emptive vaccination would be cost-saving if it increased the seroprevalence among MSM attending SHS by ≤9·0% in absolute terms over 5 years. This occurs with a yearly pre-outbreak vaccination coverage rate among SHS attendees of ≤9·1% over 5 years. For instance, an annual vaccination coverage rate of 9% shortens the modelled outbreak to 1054 days with 571 cases (Table 2), which saves 2283 QALYs (compared to no control measures) over 10 years and reduces the total costs (outbreak plus vaccination costs) from £120 million to £11 million. Vaccination strategies that increase the seroprevalence further become less cost-effective because they go beyond the herd immunity threshold (1-1/R_0_= 68·7%). Additional model projections suggest that higher rates of pre-emptive vaccination become cost-effective if the initial seroprevalence was lower (Table S7).

### Reactive vaccination strategies

For the same modelled outbreak in 2023, adding reactive vaccination in SHS clinics and primary care once the outbreak starts shortens the outbreak to 1597 days with 3770 cases (Figure 3 and Table 2). This initiative saves 2139 QALYs over 10 years compared to having no outbreak control measures and reduces the total outbreak cost to £14 million. Comparing to pre-emptive vaccination (Table 2), our results suggest that if the pre-emptive vaccination increases seroprevalence among SHS attendees by 9% in absolute terms over 5 years then reactive vaccination saves less costs than pre-emptive vaccination and gains less QALYs (Table 2), whereas this is reversed if the pre-emptive vaccination increases seroprevalence among SHS attendees by ≤7% over 5 years. This indicates that pre-emptive vaccination is the best first choice if it achieves sufficient levels of vaccination, otherwise reactive vaccination is better.

### Combined vaccination strategies

Our analyses of combined vaccination strategies show that it is cost-saving to add reactive vaccination to pre-emptive vaccination if the pre-emptive initiative does not increase the seroprevalence among MSM attending SHS by over 8·0% in absolute terms over 5 years (Table 2). For instance, if the pre-emptive vaccination initiative increases seroprevalence among SHS attendees by 7% over 5 years, then adding reactive vaccination would further reduce the total outbreak costs by £48 million and save 793 QALYs. This suggests that reactive vaccination should be added to pre-emptive vaccination initiatives if the pre-emptive initiative has not increased seroprevalence by such an extent that reactive vaccination achieves little additional impact (Table 2).

### Sensitivity analysis

The effects of our sensitivity analyses on the cost-effectiveness of vaccination initiatives are generally small (Table 3), with their effect on the transmission dynamics given in SI.4. For a pre-emptive vaccination initiative that increases seroprevalence among SHS attendees by 7% in absolute terms over 5 years, our sensitivity analyses show that pre-emptive vaccination remains cost-saving, with the costs savings and/or QALYs gained only varying by over 20% in relative terms if mildly symptomatic infected MSM (non-hospitalised) transmit HAV (SI.4.9), utilities for HAV are decreased by 10%, (SI.4.13) SHS attendees have higher immunity (SI.4.8), immunity does not decrease between the outbreaks with no pre-emptive vaccination (SI.4.6), the contact rate reduces during the outbreak (SI.4.12), just high-risk MSM attending SHS are vaccinated (SI.4.2), or there is moderate assortative mixing (SI.4.7). Reactive vaccination also remains cost-saving across all sensitivity analyses, with the cost-savings and/or QALYs gained of reactive vaccination only varying by more than 20% in relative terms if mildly symptomatic infected MSM transmit HAV, utilities for HAV are decreased by 10%, the contact rate decreases during the outbreak, or if immunity does not decrease between the outbreaks. Lastly, for a pre-emptive vaccination initiative that increases seroprevalence among SHS attendees by 7% over 5 years, adding reactive vaccination remains cost-saving (Table 3) for all sensitivity analyses except if the contact rate decreases during the outbreak or immunity remained steady between the outbreaks without pre-emptive vaccination. This again changes if the pre-emptive vaccination initiative increases seroprevalence among SHS attendees by 9% in absolute terms over 5 years, whereupon adding reactive vaccination is no longer cost-effective unless mildly symptomatic MSM still transmit HAV or both vaccination strategies only targeted high-risk MSM (Table S9). Interestingly, increasing the time between outbreaks from 5 to 10 years does increase the overall levels of vaccination needed but has little effect on the cost-effectiveness results (Table S14).

**Table 3:** Sensitivity analysis on the costs (in millions of GB£), QALYs and incremental cost-effectiveness ratio (ICER in cost per QALY saved) of pre-emptive (PV) or reactive vaccination (RV) strategies, solely or in combination. The ICERs of the single interventions are compared to a counterfactual of no vaccination, while the combined scenario considers the ICER of adding reactive vaccination to the pre-emptive vaccination initiative that increases seroprevalence among SHS attendees by 7% over 5 years.

Scenario	No Intervention		Just pre-emptive vaccination of PV=7% compared to no intervention			Just reactive vaccination compared to no intervention			Both scenarios combined incremental to pre-emptive vaccination of PV=7%		
	Total Cost	Total QALYs	Incremental cost	Incremental QALYs	ICER	Incremental cost	Incremental QALYs	ICER	Incremental cost	Incremental QALYs	ICER
Baseline*	119·5	4,121,629	-61·1	1482·7	CS	-105·3	2138·8	CS	-47·95	793·1	CS
RV delayed to 52 weeks into outbreak	119·5	4,121,629	-61·1	1482·7	CS	-100·9	2091·0	CS	-47·58	787·9	CS
Contact rate reduces by 16·9% from week 49	40·4	4,123,199	-30·7	704·3	CS	-29·67	616·5	CS	0·65	4·99	CS
Vaccination of high-risk SHS attendees only	119·5	4,121,629	-32·3	611·0	CS	-109·2	2185·8	CS	-78·32	1623·8	CS
Alternative outbreak criterion (50 cases over 3 months)	119·4	4,121,631	-60·9	1480·1	CS	-105·3	2136·8	CS	-48·04	794·1	CS
10-year gap between outbreaks	119·8	4,121,245	-69·8	1646·8	CS	-124·5	2491·3	CS	-57·90	1010·7	CS
Steady immunity between outbreaks	49·9	4,122,941	-42·5	966·0	CS	-41·0	890·4	CS	0·59	5·0	117,842
Moderate assortative mixing parameter (b=20%)	122·7	4,121,582	-46·4	1103·0	CS	-107·5	2173·9	CS	-65·57	1216·8	CS
SHS attendees have higher immunity (1:0·9:0·8:0·7)	109·4	4,121,800	-96·8	2116·0	CS	-96·2	1979·9	CS	-5·27	82·2	CS
Mildly symptomatic MSM are sexually active	338·4	4,117,885	-88·0	1516·8	CS	-275·6	5313·5	CS	-198·3	4001·2	CS
5-year immunity for 1^st^ dose	119·5	4,121,629	-60·7	1482·7	CS	-105·3	2138·8	CS	-47·95	793·1	CS
75% return rate for 2^nd^ dose	119·5	4,121,629	-61·2	1482·7	CS	-103·5	2116·3	CS	-47·85	791·8	CS
Double number of reactive vaccinations done per case (K^PC^=2*3·31,K^SHS^=2*34·41)	119·5	4,121,629	-61·1	1482·7	CS	-109·3	2215·0	CS	-47·76	798·7	CS
Utility weight reduced by 10% (absolute) for HAV-related states· Other health states remain at 0·90·	119·5	4,121,629	-61·1	2057·4	CS	-105·3	2968·6	CS	-47·95	1101·3	CS

CS denotes cost saving where the option is cheaper compared to the comparator and QALYs are gained. KPC is the daily number of reactive vaccinations given in primary care per infection on the previous day and KSHS is the daily number of reactive vaccinations given in sexual health services per infection on the previous day. * The baseline scenario is defined by: an outbreak start criterion of >=30 cases reported within 3 month and ends when there is <=1 case within one month; the gap between outbreaks is 5 years; immunity decreases between outbreaks due to inflow of susceptible and outflow of immune MSM; mildly symptomatic MSM are not sexually active; weak assortative mixing (b=3·5%); equal initial immunity levels among 4 MSM sub-groups (high and low risk and SHS attendance or not); the utility weights are 0·83 for asymptomatic, 0·64 for symptomatic, 0·26 for fulminant, 0·73 for post liver transplant cases, and 0·90 for other health states. Baseline vaccination is to all SHS attendees equally with RV starting immediately once the outbreak starts (if RV is done) and no change in contact rate during the outbreak. Effect of first vaccine dose is assumed to last 7 years and 50% return for second dose.

The sensitivity analysis where we assumed a lower number of cases in the 2016/18 outbreak did not result in major changes to the resulting parameters for the 2023 outbreak model (Table S8) and so was not considered further.

## Discussion

Our modelling of the HAV outbreak among MSM in England in 2016/18 suggested that despite HAV being highly transmissible (R_0_=3·19), the outbreak was limited by existing high levels of immunity (69·1%) and a modest reduction (16·9%) in contact rates during the outbreak. Going forward, our modelling of the hypothetical outbreak in 2023 show that reactive vaccination of MSM during future outbreaks can be a cost-saving strategy for substantially reducing its magnitude. However, pre-emptive vaccination of MSM in sexual health services (SHS) is likely to be more cost-effective if the vaccination rate is sufficiently high, suggesting that this is the optimal first choice. Importantly, it can also be cost-effective to add reactive vaccination to pre-emptive vaccination initiatives if the coverage of pre-emptive vaccination is not too high and MSM contact rates do not decrease during the outbreak due to public health messaging.

During the 2016/18 HAV outbreak, Public Health England (PHE) recommended reactive vaccination of MSM through SHS.^11^ Concurrently, PHE also raised awareness of the outbreak and followed up cases and their close contacts to minimise the risk of transmission by providing hygiene advice and post-exposure vaccination.^9^ Our modelling suggests these public health measures may have reduced contact rates among MSM, reducing the outbreak by a third and being the main cause for its downturn. The reactive vaccinations had much less impact because they started late in the outbreak (after July 2017) due to a national vaccine shortage and other challenges. This contrasts with our projections going forward that show reactive vaccination could have considerable impact if initiated early in an outbreak. This emphasises the importance of early recognition of outbreaks and having vaccine supply and delivery systems in place to respond rapidly.^26^

Our estimates of R_0_ for HAV among MSM in England (R_0_=3·19) are in agreement with estimates from persons experiencing homelessness or who use drugs,^26^ larger but comparable to estimates from MSM elsewhere (R0=1·4-3·1),^10^^,^^27^^,^^28^ and much larger than estimates for other groups,^29^^,^^30^ emphasising the increased HAV risk in MSM. There are limited studies of the cost-effectiveness of HAV vaccination among MSM. Our findings are consistent with a US study reporting that Hepatitis A and B vaccination of individuals attending STI clinics is cost-effective, although they did not distinguish MSM from non-MSM,^31^ and another study reporting that HAV vaccination is cost-effective among MSM in USA.^32^ Although limited, this evidence agrees with our study that targeted HAV vaccination of individuals with heightened sexual risk is value for money.

### Strengths and limitations

Strengths of our analysis include calibrating our model to a recent large and prolonged HAV outbreak among MSM. This provided unique and rich information about the transmissibility of HAV among MSM in England and gave insights into why the outbreak died out. We also stratified MSM by levels of sexual risk behaviour and their attendance of SHS, which allowed us to assess the impact of vaccinating specific MSM subpopulations.

Limitations include uncertainty in numerous model parameters. Firstly, we assumed the same baseline seroprevalence among all MSM sub-groups. Although, it is likely that high-risk MSM and MSM attending SHS may have greater immunity due to increased chances of being infected or vaccinated, our sensitivity analyses suggest this should not have changed our conclusions. Additionally, we assumed the seroprevalence would decrease over time following the 2016/18 outbreak due to population turnover, and that the next outbreak would occur in 2023, which is highly uncertain. Although future outbreaks will tend to be bigger if there is a longer time gap, with vaccination having greater impact, it did not significantly affect the cost-effectiveness. Indeed, vaccination would remain cost-saving even if seroprevalence remained stable between the outbreaks. We also did not incorporate vaccination of MSM travelling to high-risk countries or for other reasons (∼60-70,000 vaccinations a month through primary care in 2016, NHS data),^5^^,^^33^ or immigration of MSM born in endemic countries who are likely to have pre-existing immunity, which may result in seroprevalence declining less between outbreaks. We also did not consider the potential emergence of vaccine-escape variants, which may possibly occur if some MSM receive post exposure-vaccination,^34^ because there is little data on its clinical or public health significance.

Secondly, we did not include age structure in our model, which can be associated with HAV seroprevalence, exposure risk and clinical presentation.^14^ Our modelling shows that targeting pre-emptive vaccination to high-risk MSM attending SHS clinics reduces the cost savings and QALYs gained compared to vaccinating all SHS attendees, suggesting that prioritising younger MSM may have no added benefit.

Thirdly, there were uncertainties around behavioural parameters such as levels of assortative mixing and whether mildly symptomatic MSM are sexually active. Our sensitivity analyses suggest these uncertainties do not affect our findings.

### Implications

Our study has implications for preventing HAV outbreaks. We found that although the transmissibility of HAV is high among MSM in England, existing high levels of immunity have prevented large HAV outbreaks. This immunity is best maintained in the future through pre-emptive vaccination strategies, as recommended by UKHSA,^5^ and then possibly reactive vaccination strategies if the pre-emptive vaccination coverage is insufficient to prevent an outbreak. Although our analyses suggest this can be achieved through solely vaccinating in sexual health services, we would also recommend that MSM should be vaccinated in other settings to ensure high coverage is achieved. Going forward, robust surveillance is needed to ensure that reactive vaccination is initiated promptly when an outbreak starts, but is not initiated due to natural variation in cases. Acting quickly requires a stable supply of vaccines, pre-agreed commissioning arrangements, and logistics and staff to rapidly scale-up. Although this can be challenging, our vaccine response to COVID-19 shows that it is possible. Lastly, improved monitoring of HAV seroprevalence among MSM is also needed to ensure that immunity levels are not decreasing so increasing the likelihood of future outbreaks.

## Contributors

XSZ, SM, PV, and JJO conceptualized and developed the analysis methodology. XSZ developed the model and performed all model analyses. All authors undertook data analyses for parameterising and calibrating the model. PV, JJO, ME and SM supervised the overall analysis, with PV, LM and JJO supervising the modelling and economic evaluation. All authors reviewed the analysis methodology and model results. XSZ and PV wrote the original draft of the manuscript. All authors reviewed and edited the manuscript.

## Data sharing statement

The model code and projections for this paper will be shared with interested parties upon reasonable request, which will be decided by Peter Vickerman and Xu-Sheng Zhang.

## Declaration of interests

PV has received unrestricted research grants from Gilead not related to the submitted work. This research was funded in whole, or in part, by the National Institute for Health Research Health Protection Research Unit for Behavioural Science and Evaluation and the Wellcome Trust [WT 220866/Z/20/Z].

## Appendix Supplementary materials

Image, application 1
