# Supplementary material for: Transmission dynamics of the 2016-18 outbreak of hepatitis A among men who have sex with men in England and cost-effectiveness analysis of vaccination strategies to prevent future outbreaks
Source: Lancet Reg Health Eur. 2022 Jun 17;19:100426. doi: 10.1016/j.lanepe.2022.100426 (PMC9417902; doi:10.1016/j.lanepe.2022.100426)
Supplement: Supplementary file 1 [file mmc1.docx]

**Supplementary Material**

**Transmission dynamics of the 2016-18 outbreak of hepatitis A among men who have sex with men in England and the cost-effectiveness of vaccination strategies to prevent future outbreaks**

Xu-Sheng Zhang^1^, Jason J. Ong^2,3^, Louis Macgregor^2^, Tatiana G Vilaplana^4^, Simone Thorn Heathcock^5^, Miranda Mindlin^6^, Peter Weatherburn^7^, Ford Hickson^7^, Michael Edelstein^4^, Sema Mandal ^2,4^ & Peter Vickerman^2^

1. Statistics, Modelling and Economics, Data, Analytics & Surveillance, UK Health Security Agency, UK
2. University of Bristol, Bristol, UK
3. Monash University, Melbourne, Australia
4. Immunisation and Vaccine Preventable Diseases Division, UK Health Security Agency, UK
5. North East London Health Protection Team, UK Health Security Agency, UK
6. South London Health Protection Team, UK Health Security Agency, UK
7. London School of Hygiene & Tropical Medicine, UK

Corresponding author: Peter Vickerman

**Content**

**page**

**SI.1 Technical details of Method and Models 3**

**SI.1.1 Contact rate parametrization 3**

**SI.1.2 Two types of vaccinations: V^SHS^ and V^PC^ 5**

**SI.1.3 Force of infection and imported infections 5**

**SI.1.4 Parameter Inference 6**

**SI.2 Design of vaccination to control the 2023 hypothetical outbreaks 8**

**SI.2.1 Pre-emptive vaccination during a non-outbreak period 8**

**SI.2.2. Reactive vaccination during an outbreak period 12**

**SI.3 Estimating health benefits and costs**  **12**

**SI.3.1 Health benefits 13**

**SI.3.2 Productivity losses 13**

**SI.3.3 Costs of the outbreak 13**

**SI.3.4 Cost-effectiveness of vaccination strategies 14**

**SI.3.5 Simulations 14**

**SI.4 Sensitivity analyses 17**

**SI.4.1 Outbreak only restricted to 507 MSM cases during 2016-18 20**

**SI.4.2 Target pre-emptive and reactive vaccination strategies to high risk MSM who attend SHS clinics 20**

**SI.4.3 Effect of the outbreak criterion 22**

**SI.4.4 Effect of late start of reactive vaccination 24**

**SI.4.5 The effect of different non-outbreak periods 27**

**SI.4.6 Steady seroprevalence between outbreaks 29**

**SI.4.7 Effect of assortative mixing among high risk MSM 31**

**SI.4.8 The effect of variation in the protection levels among four groups of MSM 33**

**SI.4.9 Effect of transmission of mildly symptomatic cases 35**

**SI.4.10 Effects of duration of immunity induced by one dose of vaccine and the return rate for 2^nd^ dose**

**of vaccination. 40**

**SI.4.11 Enhanced reactive vaccination 41**

**SI.4.12 Effect of reduction in contact rate due to sexual behavioural change during the outbreak 43**

**SI.4.13 Effect of variation in utility weights on the cost-effectiveness of vaccination strategies 45**

**Other Tables Table S6 47**

**References 48**

**SI.1 Technical details of Method and Models**

***Transmission dynamics model of Hepatitis A outbreak***

The diagram of the transmission model is shown in Figure 1 and the model equations are given by:

$\frac{dS_{j}(t)}{dt}=-\lambda_{j}S_{j}\left( t \right)-\Omega_{j}\left( t \right)\delta\left( t-t^{import} \right)-\left( t-t^{vacc} \right)(\frac{S_{j}\left( t \right)}{N_{j}^{*}\left( t \right)})[\frac{V_{j}^{SHS}\left( t \right)}{1+q}+V_{j}^{PC}$(t)],

$\frac{dE_{j}\left( t \right)}{dt}=\lambda_{j}S_{j}\left( t \right)-\frac{E_{j}\left( t \right)}{L}$,

$\frac{dO_{j}\left( t \right)}{dt}=\frac{E_{j}\left( t \right)}{L}+\Omega_{j}\delta\left( t-t^{import} \right)-\frac{O_{j}\left( t \right)}{d_{1}}$,

$\frac{dD_{j}(t)}{dt}=p_{S}\frac{O_{j(t)}}{d_{1}}-\frac{D_{j}(t)}{d_{D}}$, (S1)

$\frac{dA_{j}(t)}{dt}={(1-p}_{S})\frac{O_{j}(t)}{d_{1}}-\frac{A_{j}\left( t \right)}{d_{2}}$,

$\frac{dR_{j}(t)}{dt}=\frac{D_{j}(t)}{d_{D}}+\frac{A_{j}(t)}{d_{2}}+\left( t-t^{vacc} \right)(\frac{S_{j}\left( t \right)}{N_{j}^{*}\left( t \right)})[\frac{V_{j}^{SHS}\left( t \right)}{1+q}+V_{j}^{PC}(t$)].

Here $N_{j}^{*}\left( t \right)=N_{j}{-D}_{j}(t)$ is the number of MSM in group *j* without illness due to HAV. The definitions of other variables and parameters are given in Table S1, and the detailed explanations of equation (1) are given below.

**Table S1 Definitions of variables and parameters in equations (S1)**

| **Variable** | **Definition** |
| --- | --- |
| *S*_j_ | No of susceptible MSM in group *j* |
| *E*_j_ | No of exposed MSM in group *j* |
| *O*_j_ | No of occult infected MSM in group *j* |
| *D*_j_ | No of symptomatic cases in group *j* |
| *A*_j_ | No of asymptomatic cases in group *j* |
| *R*_j_ | No of MSM under protection in group *j* |
| V^SHS^_j_(t) | No of vaccinations ordered through SHS clinics (*j*=2,4) on day t |
| V^PC^_j_(t) | No of contact tracing vaccinations in primary care on group *j* on day t |
| **Parameter** | **Definition** |
| *q* | Fraction of MSM who have 2 doses of vaccine via V^SHS^ |
| *λ*_j_ | Force of infection for group j |
| 1-*w* | Reduction in contact rate due to public health response |
| *Ω*_j_(*t*) | No of imported occult infections in group *j* on day *t* |
| *ρ* | Initial proportion susceptible to HAV infection before the outbreak |
| *p*_S_ | Proportion of infections that develop symptoms |
| *L* | Latent period |
| *d*_1_ | Average delay from occult infection to symptom onset |
| *d*_D_ | Average infectious period for symptomatic cases |
| *d*_2_ | Average infectious period for asymptomatic case |
| *t*^import^ | The return dates of 39 imported cases during the 2016/18 outbreak |
| δ(t-t^1^) | Kronecker delta |

**SI.1.1 Contact rate parametrization**

The size of the MSM population in England was estimated to be 531,559 in 2018[1]. The distribution of total number of sexual partners in the last 12 months reported by MSM in the UK component of the European MSM Internet Survey (EMIS-UK) [2] was used to determine a cut-off for defining the high and low sexual risk MSM groups. EMIS was an online survey undertaken during June-August 2010, recruiting online and promoted offline through print media. Over 18,000 MSM living in the UK participated. EMIS-UK data also determined the frequencies of sexual partnerships for MSM in the low and high-risk groups. We chose the cut-off for high risk as greater than 10 partners in last 12 months due to the shape of the histogram in Figure S1.


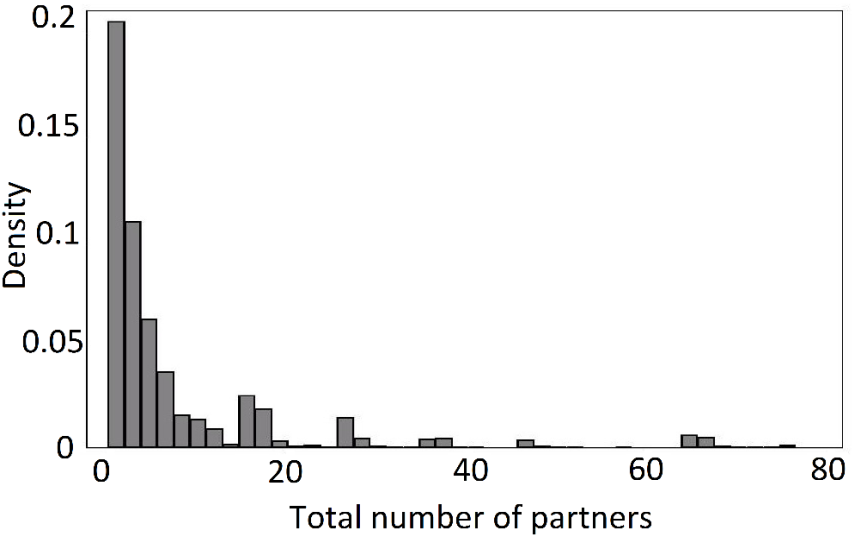


**Figure S1** **Histogram of the distribution of total number of sexual partners in the last year as given by EMIS-UK data**

The average number of partners in the last 12 months was assumed to be 2.3 in the low risk group and 17.9 in the high-risk group. Breaking down the EMIS dataset by sexual health service (SHS) clinics attendees and <10 partners *vs* 10+ partners in the last 12 months, we have the following proportions of MSM in the four groups of MSM: 47.3%, 31.0%, 6.7% and 15.0% for groups *j*=1 (non_SHS & low risk), 2 (SHS & low risk), 3 (non_SHS & high risk), and 4 (SHS & high risk), respectively.

Based on this cut-off of 10 partners, the mixing matrix among low and high-risk groups of MSM is given by

M_LL_= *b*+(1-*b*)*EM_LL_

M_LH_ = (1-*b*)*EM_LH_

M_HL_ = (1-*b*)*EM_HL_

M_HH_ = *b*+(1-*b*)*EM_HH_

where EM is the contact matrix if sexual mixing was random based on the number of sexual partnerships provided by MSM in the different subgroups:

$\mathrm{EM}=\left( \begin{matrix} 0.253 & 0.747 \\ 0.253 & 0.747 \end{matrix} \right)$

and *b* is the parameter which characterises the extent of assortative or like-with like mixing behaviours among high risk MSM [2]. From fitting to EMIS dataset on the degree of mixing, the mixing parameter *b* is estimated to be 3.5% for MSM in England [2]. Applying this estimate to the above equation, we have the mixing matrix among Low and High risk groups as:

$M_{Risk}=\left( \begin{matrix} 0.280 & 0.720 \\ 0.245 & 0.755 \end{matrix} \right)$.

(The effect of strong assortative mixing among high risk MSM on transmission dynamics will be investigated in SI.4.7)

Similarly, the mixing matrix among Non-SHS and SHS attendees is

$M_{SHS}=\left( \begin{matrix} 0.162 & 0.838 \\ 0.154 & 0.846 \end{matrix} \right)$.

Assuming the independence between risk and SHS attendance, the mixing matrix among these four groups is

$${\boldsymbol{M}=M}_{Risk}\otimes M_{SHS}=$$

$\left( \begin{matrix} \begin{matrix} 0.280\times0.162 & 0.280\times0.838 \\ 0.280\times0.154 & 0.280\times0.846 \end{matrix} & \begin{matrix} 0.720\times0.162 & 0.720\times0.838 \\ 0.720\times0.154 & 0.720\times0.846 \end{matrix} \\ \begin{matrix} 0.245\times0.162 & 0.245\times0.838 \\ 0.245\times0.154 & 0.245\times0.846 \end{matrix} & \begin{matrix} 0.755\times0.162 & 0.755\times0.838 \\ 0.755\times0.154 & 0.755\times0.846 \end{matrix} \end{matrix} \right)$.

**SI.1.2 Two types of vaccinations: V^SHS^ and V^PC^**

We consider reactive vaccination via SHS clinics and via primary care during the 2016/18 outbreak which were included in equation (S1). The vaccination is modelled by moving vaccinated people into immune class immediately.

V^SHS^ –vaccination via SHS clinics, started from July 2017 with the number of vaccines ordered from July 2017 to July 2018 listed in Table S2. [Note, reactive vaccination in SHS was recommended from April 2017 prioritised for MSM in SHS. To assess the effect of early opportunistic vaccinations, we undertake a sensitivity analysis in supplementary Information SI.4 (Table S8**)** where we assume reactive vaccinations started from April 2017, but with lower rates during the three months from April to July 2017.]

**Table S2 Vaccine dose orders through SHS clinics during the 2016/18 outbreak**

| Jul17 | Aug17 | Sep17 | Oct17 | Nov17 | Dec17 | Jan18 | Feb18 | Mar18 | Apr18 | May18 | Jun18 | Jul18 |
| --- | --- | --- | --- | --- | --- | --- | --- | --- | --- | --- | --- | --- |
| 3292 | 4437 | 4147 | 2513 | 1559 | 901 | 1179 | 1244 | 1073 | 1480 | 1556 | 2367 | 1643 |

For simplicity we assume that each vaccine ordered resulted in one MSM being vaccinated and that the vaccination took place daily only among SHS clinic attendees (i.e. those in group 2 and 4). We assume the daily number of vaccinations via SHS clinics is given at an average daily frequency with all SHS attendees having an equal likelihood of vaccination. For example, for each day of July 2017,

$V_{1}^{\mathrm{SHS}}\left( t \right)=0$,$V_{2}^{\mathrm{SHS}}\left( t \right)=\frac{N_{2}}{N_{2}+N_{4}}(\frac{3292}{31})$,

$V_{3}^{\mathrm{SHS}}\left( t \right)=0$,$V_{4}^{\mathrm{SHS}}\left( t \right)=\frac{N_{4}}{N_{2}+N_{4}}(\frac{3292}{31})$, *t*=1, …,31 July 2017

Here 3294 were the number of vaccines given out in July 2012 and *N*_j_ is the size of group *j*=1,2,3,4

V^PC^ – the vaccinations given to the contacts of reported cases through primary care. The average number of contacts per case in primary care is κ^PC^=3.31 (95%CI: 2.96, 3.67) and so in total 725×3.31≅2400 MSM were vaccinated in this way. We assume that V^PC^ started from 1^st^ July 2017 and was equally distributed at a daily rate from 1^st^ July 2017 to 31^st^ July 2018 among all 4 groups of MSM in accordance with their relative population sizes as follows

$V_{j}^{\mathrm{PC}}\left( t \right)=\frac{N_{j}}{N}\kappa^{\mathrm{PC}}(\frac{725}{365+31})$, *t*=1^st^ July 2017(day 1), …,31^st^ July 2018(day 396), *j*=1,…,4

Here *N* represents the total number of MSM and 725 is the total number of male cases during the outbreak.

**SI.1.3 Force of infection and imported infections**

In equation (S1) the parameter λ_j_ represents the force of infection for group *j* (=1,2,3,4 corresponding to the four groups of MSM defined in SI.1.1). One aspect of public health response is to raise public and professional awareness of the outbreak and follow up of cases and their contacts to minimise risk during sexual contacts [3]. These outbreak responses gave rise to behavioural changes which encapsulate both change in number of partners (contacts –risk of being exposed) as well as change in risky behaviour during sexual transmission (e.g., washing –i.e., hepatitis A being different from classical STIs as route of transmission is faecal-oral). To reflect this change [4, 5], we introduce a composite behaviour response parameter *w* in the force of infection as follows

$\lambda_{j}\left( t \right)=\left\{ \begin{matrix} \begin{matrix} \beta\sum_{i=1}^{4} \Pi_{j,i}\left( O_{i}\left( t \right)+A_{i}\left( t \right) \right) & t<t_{c} \end{matrix} \\ \begin{matrix} w\beta\sum_{i=1}^{4} \Pi_{j,i}(O_{i}\left( t \right)+A_{i}(t)) & t\geq t_{c} \end{matrix} \end{matrix} \right.$ (S2)

That is, we simply assume the composite behavioural response takes place at time *t*_c_, after which the effective contact rate reduces to *w* (<1) relative to the previous. Here we assume the composite behaviours that could change to include multiple factors such as sexual contact, safer sex, condom use, taking better care, personal hygiene, public health actions, etc. that can reduce the risk of exposure to HAV. In equation (S2), the matrix

$\Pi_{j,i}(t)={\boldsymbol{M}_{j,i}}/\Lambda$ (S3)

is a scaled version of the mixing matrix, with Λ being the dominant eigenvalue of the next generation matrix ***M****, whose element (*j*,*i*)*^th^* is given by $M_{j,i}^{*}={N_{j}M}_{j,i}$, where *N_j_* is the population size within group *j*. ${\beta\Pi}_{j,i}$ gives the infection pressure exerted on a susceptible individual within group *j* by a single infectious individual in group *i*.

*Ω*_j_($t^{import}$) in equation (S1) represents the number of travellers infected that belong to group *j* and returned to England on day $t^{import}$. δ$(t-t^{import})$ is the kronecker delta: it is 1 when *t* = $t^{import}$ and 0 otherwise. We assumed that imported cases are those who travelled prior to December 31^st^, 2016 and whose symptom onset occurred within the incubation period (15-50 days)[6, 7] before December 31^st^ 2016. In accordance with this definition, we found from the 2016/18 outbreak data in England that there were 39 imported cases among 725 cases and their return dates, represented by $t^{import}$. Among these imported cases, 37 had their symptom onset dates later than or identical to the return dates. In view of this, we simply assume that all the 39 imported cases were susceptible when leaving England but were in occult infection when returning. As the information of groups to which each of the 39 imported cases belonged was lacking, the imported cases were assumed to be only distributed among the high-risk groups (i.e., group 3 and 4) as

${}_{j}(t)=X_{j}(t)$ (S4)

with *Φ*(*t*) being the number of imported cases on day *t* and the vector *Χ* being in proportion to the sizes of the two high risk groups and given by *Χ*_1_=0; *Χ*_2_=0; *Χ*_3_= χ_3_/(χ_3_ + χ_4_); *Χ*_4_ = χ_4_/(χ_3_ + χ_4_). Here χ is the eigenvector of mixing matrix ***M***.

The basic reproduction number for hepatitis A, which is defined as the average number of new cases generated by an infected person in a completely susceptible population [8, 9], is given by the following expression [4, 10],

$R_{0}=\left[ d_{1}+(1-p_{S})d_{2} \right]$ (S5)

Here *β* is the transmission coefficient, *d*_1_ and *d*_2_ are the infectious periods of occult infection and asymptomatic infection, respectively, and *p*_S_ is the fraction of asymptomatic infections.

**SI.1.4 Parameter Inference**

To estimate model parameters Θ = {*β*, *ρ*, *t*_c_, *w*} as defined in Table S1, the two likelihood functions involved in the Bayesian Markov Chain Monte Carlo (MCMC) framework are: *Poisson likelihood function*

$l_{ST}\left( x\left( t \right) | \Theta, \right)=\frac{\exp(-\left( t \right))}{x\left( t \right)!}{(t)}^{x(t)}$ (S6a)

where *x*(*t*) is the reported numbers of cases on week *t* and *μ*(*t*) is the predicted number of the cases from equation (S1) of the transmission dynamics model given by,

$\mu\left( t \right)=(p_{S}/d_{1})\int_{t-1}^{t} \sum_{j=1}^{4} O_{j}()d$ (S6b)

and *beta likelihood function*

$l_{Post}\left( z | \Theta\right)=\frac{\Gamma(\nu_{1}+\nu_{2})}{\Gamma(\nu_{1})\Gamma(\nu_{2})}z^{\nu_{1}}\left( 1-z \right)^{\nu_{2}}$ (S7)

where *z* is the predicted seroprevalence post the outbreak given by

$z=\frac{1}{N}\sum_{j=1}^{4} R_{j}(T=98)$

with *R_j_*(T=98) representing the number of group *j* by week 98 since July 2016 that were immune/seropositive either through vaccination from July 2017 or natural infections. The two shape parameters of beta likelihood function are given by

$\nu_{1}=m(\frac{m\left( 1-m \right)}{v}-1)$, $\nu_{2}=(1-m)(\frac{m\left( 1-m \right)}{v}-1)$,

with *m* and *v* representing the mean and variance of seroprevalence post the outbreak obtained from Table S3.

Assuming that the weekly incidence observed: *x*(1), *x*(2), …, *x*(T) are conditionally independent, the total log-likelihood given model parameters Θ is

$L\left( \Theta,\eta;x,z \right)=\sum_{t=1}^{T} {log(l}_{ST}\left( x\left( t \right) | \Theta,\eta\right))+Tlog(l_{Post}\left( z | \Theta\right))$ (S8)

Here we introduce the weight *T* to likelihood due to the seroprevalence after the outbreak because it is the cumulative change over *T* weeks.

The prior distribution *f*(Θ) of model parameters are drawn from literature (see Table 1). Employing Bayesian framework through the combination of the prior distribution *f*(Θ) and the likelihood *L*(Θ,*η*; *x*, *z*), the posterior distribution can be sampled by Monte-Carlo Markov Chain (MCMC). From these samples, we can obtain means and their 95% credible intervals for the model parameters.

**Table S3 Seven surveys on hepatitis A seroprevalence of MSM attending SHS in England**

| **Study** | **Sample period** | **Sample age** | **Mean seroprevalence** | **References** |
| --- | --- | --- | --- | --- |
| Brighton | 2019 | All age | 56.8% | [11] |
| Durham and Darlington | March to August 2017 | Under 50 | 42.1% | [12] |
| Leeds | April to July 2017 | Under 45 | 56.4% | PHE data |
| St Thomas (London) | October to December 2018 | Under 45 | 70.1% | PHE data |
| Imperial (St Mary) | August to October 2018 | Under 45 | 82.1% | PHE data |
| Homerton (London) | January to July 2018 | Under 45 | 69.3% | PHE data |
| TDL (Northwick Park, Central Middlesex, Tudor Centre) | November 2017-September 2018 | Under 45 | 83.6% | PHE data |
| Pooled mean=65.8%, standard deviation =15.0%, 95% confidence interval: 54.9-76.9% | | | | |

The age distribution of immunity levels collected from surveys over the four sites in London

| **Age group (middle age)** | **Number of samples** | **Number of positives** | **positivity** |
| --- | --- | --- | --- |
| 18-25 (21.5) | 525 | 320 | 61.0% |
| 26-35(30.5) | 979 | 693 | 70.8% |
| 36-45(40.5) | 600 | 497 | 82.8% |
| 46 plus | 473 | 409 | 86.5% |
| The positivity linearly extrapolated to age 15 years old is 54.0% | | | |

**SI.2 Design of vaccination to control the 2023 hypothetical outbreaks**

**SI.2.1 Pre-emptive vaccination during a non-outbreak period**

Because of the short delay from vaccinated to protected (about two weeks), the MSM population during the non-outbreak time is divided into three compartments: *S*_j_ – the number of the susceptible, *R*_j_ – the number of MSM that have 1 dose of vaccine and are under short-term protection (1/ζ= 84 months); *P*_j_ – the number of MSM that have 2 doses of vaccine and are under long-term protection (lifelong). The total population size of group *j* is *N*_j_= *S*_j_ + *R*_j_ + *P*_j_. Further, as the non-outbreak period is longer than the 2016/18 outbreak (about 5 years, also consider 10 years in SI.4.5), we include entry and exit from the MSM population and fix its rate *α* at 1/((49-15+1)×365) per day by assuming that the sexual active MSM population are those of ages from 15 to 49 years old men. Note that the men of ages less than 15 years are not necessarily completely immunologically naïve to hepatitis A because of exposure during travel abroad or community infections during their childhood (see Table S3). The variables and parameters of pre-emptive vaccination dynamics are listed in Table S4. We will also consider another situation where vaccines are given only to high-risk SHS clinic attendees (SI.4.2).

**Table S4 Definition of parameters for pre-emptive vaccinations and associated costs**. We model 3 pre-emptive vaccination scenarios: 1) vaccinate without testing, 2) test then vaccinate, and 3) test and vaccinate (see below for details)

| **Parameter** | **Definition** | **Value** |
| --- | --- | --- |
| *T*_c_ | Cost of hepatitis A antibody screening test | £10.00 |
| *V*_c_ | Cost of hepatitis A vaccine per dose | £20.00 |
| *A*_c_ | Cost for each administration of vaccine | £10.00 |
| *G*_c_ | Cost for SHS consultation | £169.51 |
| *c_j_*^♠^ | Vaccination Coverage at group *j* (=1,2,3,4) | – |
| *r* | Proportion of 1^st^ SHS clinic visit time allotted to vaccination | 0.25 |
| *r*_2_ | Proportion of 2^nd^ SHS clinic visit time allotted to vaccination | 0.0 |
| *q*^♥^ | Proportion of those having first dose of vaccine that have 2^nd^ dose | 0.50 |
| *τ_j_* | Testing rate among group j for scenario 2 | – |
| *m* | Proportion of MSM that return after test for result and vaccination | 0.50 |

^♠^: In Scenarios 1 and 3, “Coverage” is defined among the general MSM population, while for scenario 2, “coverage” is defined among only the susceptible MSM. To avoid confusion, we view the question in different angle by asking: how many people will need to be vaccinated to achieve a target increase in immunity? Then the coverage c* can be calculated for scenario 2 in the same meaning as to scenarios 1 and 3; ^♥^: *q* can be generally defined as the proportion that return for second visit among these having 1^st^ dose of vaccine. For vaccination scenarios 1 and 2, these are the people that will certainly have 2^nd^ dose of vaccine. But for scenario 3, *q* is just the proportion that return for second visit and not necessarily being given 2^nd^ dose of vaccine. Hence q* can be calculated for scenario 3.

*Three pre-emptive vaccination scenarios*

We simulated the following three pre-emptive strategies with or without testing for SHS attendees during the 5-year non-outbreak period of 2018-2023.

**Scenario 1: Vaccinate without testing –** don’t test for previous exposure or whether vaccinated, just vaccinate at SHS clinic visit – allot a proportion *r* (=25%) of the first SHS visit cost to HAV vaccination but remove cost of second SHS visit cost (*r*_2_=0) and a proportion *q* (=50%) will come back for 2^nd^ dose. Assume a yearly coverage *c*_j_ for vaccination**.**

During the non-outbreak period, vaccination dynamics is described by

$\frac{dS_{j}}{dt}=\alpha\left( {}_{Y}N_{j}-S_{j} \right)-\frac{c_{j}}{365}S_{j}+R_{j}$

$\frac{dR_{j}}{dt}=(1-q)\frac{c_{j}}{365}S_{j}-(+\alpha)R_{j}$ (S9)

$$\frac{dP_{j}}{dt}=q\frac{c_{j}}{365}S_{j}-\alpha P_{j}+\alpha\left( {1-}_{Y} \right)N_{j}$$

Here *c_j_* is the yearly vaccination rate for group j, is the rate of losing immunity after one vaccine dose, *ρ*_Y_ =1-0.54 =0.46 is the susceptibility of males aged less than 15 years old (Table S3). A simple analysis shows that if the initial proportion under lifelong protection (*P*) is *φ*_j0_ for group *j*, this proportion will increase only if

$\frac{c_{j}}{365}>\frac{{}_{j0}-\alpha\left( {1-}_{Y} \right)N_{j}}{q(1-{}_{j0})}$.

We assume the pre-emptive vaccination will only be given to SHS attendees. That is, *c*_1_ (NonSHS low-risk) = *c*_3_ (NonSHS high-risk) =0. This implies that the proportion of immunity in non-SHS attendees will reduce as the old and potential protected MSM exit and the young and less protected young MSM enter. From equation (S9), the number of immune non-SHS attendees decay following the formulae:

$P_{j}\left( t \right)=\exp\left( -\alpha t \right)P_{j}\left( 0 \right)+\alpha\left( {1-}_{Y} \right)N_{j}$, *j*=1,3.

In equation (S9), ${\frac{c_{j}}{365}S}_{j}=\frac{c_{j}}{365}\frac{S_{j}}{N_{j}}N_{j}$ represents pre-emptive vaccinations that will successfully be given to susceptible people. The remaining $\frac{c_{j}}{365}(N_{j}-S_{j})$ vaccines given will not change anyone’s status and do not appear in the above equations, which are just wasted. The total number of vaccines given through the scenario during the five-year period is:

$\int_{0}^{5*365} \sum_{j=1}^{4} \frac{c_{j}}{365}N_{j}(1+q)dt=5{\{{(c}_{2}N}_{2}+{c_{4}N}_{4})\left( 1+q \right)\}$,

which is fixed if *c*_j_ is fixed over the five-year period. Note that *c*_j_ is the yearly coverage of people being vaccinated, with all getting 1 dose, and a proportion *q* of MSM getting the 2^nd^ dose. The total cost involved with scenario 1 vaccinations is

$$5\left( {c_{2}N}_{2}+{c_{4}N}_{4} \right)\left\{ \left( V_{c}+A_{c} \right)\left( 1+q \right)+G_{c}\left( r+r_{2}q \right) \right\}.$$

Here the full SHS clinic appointment cost (G_c_) for 2^nd^ dose of vaccine is removed (*r*_2_=0), while Vc is the cost of the vaccine and Ac is the cost for each administration of vaccine. For a given yearly coverage of vaccination, the increase in the lifelong immunity level will be obtained by solving equation (S9).

**Scenario 2: Test then vaccinate –** In this scenario we test for previous exposure/vaccinated and call back to vaccinate in 2^nd^ visits if non-immune – allot a proportion *r* (=25%) of the first SHS visit cost to HAV vaccination but remove cost of second SHS visit cost (*r*_2_=0); the proportion *m* of these tested return for test result and then the proportion *q* of these return for 2^nd^ vaccine dose.

We assume the test rate within group *j* is *τ*_j_, and the proportion of those who come back for results and potential vaccination is *mτ*_j_. The returners will get 1^st^ dose of vaccine if tested negative. During the non-outbreak period, vaccination dynamics is described by

$\frac{dS_{j}}{dt}=\alpha\left( {}_{Y}N_{j}-S_{j} \right)-\frac{m{}_{j}}{365}S_{j}+R_{j}$

$\frac{dR_{j}}{dt}=(1-q)\frac{m_{j}}{365}S_{j}-(\alpha+)R_{j}$ (S10)

$$\frac{dP_{j}}{dt}=q\frac{m_{j}}{365}S_{j}-\alpha P_{j}+\alpha\left( {1-}_{Y} \right)N_{j}$$

Because of the short interval from test to vaccination (about a couple of weeks), it is not necessary to introduce another compartment for MSM that were just tested but not yet vaccinated. Here ${\frac{m_{j}}{365}S}_{j}$represents total pre-emptive vaccination given to susceptible MSM, without any waste. The total number of vaccines through pre-emptive vaccination is:

$\int_{0}^{5*365} \sum_{j=1}^{4} \frac{m{}_{j}}{365}S_{j}(t)(1+q)dt$ ,

which is dependent on how many MSM are naïve to HAV. Note that the average yearly coverage of vaccination can be calculated as

$c^{*}=(\int_{0}^{5*365} [\frac{m{}_{2}}{365}S_{2}\left( t \right)+\frac{m{}_{4}}{365}S_{4}\left( t \right)]dt)/(5N_{2}+{5N}_{4})$.

The total cost with vaccination scenario 2 is the sum of the following two costs: (1) the cost involved with tests

$5{\{(_{2}N}_{2}+{}_{4}N_{4})\left( T_{c}+rG_{c} \right)\}$,

where Tc is the cost of the test; (2) the cost involved with vaccinations

$(1+q)(V_{c}+A_{c}+r_{2}G_{c})\int_{0}^{5*365} [\frac{m_{2}}{365}S_{2}\left( t \right)+\frac{m_{4}}{365}S_{4}\left( t \right)]dt$.

**Scenario 3**: **Test and Vaccinate and Vaccinate again if non-immune –** In this scenario we test and vaccinate at first visit and call back for 2^nd^ dose only if test is negative – allot a proportion *r* (=25%) of the first SHS visit cost to HAV vaccination but remove cost of second visit (*r*_2_=0). Assume *q*=50% come back to second visit. Assume the same yearly coverage.

During the non-outbreak period, vaccination dynamics is described by

$\frac{dS_{j}}{dt}=a\left( {}_{Y}N_{j}-S_{j} \right)-\frac{c_{j}}{365}S_{j}+R_{j}$

$\frac{dR_{j}}{dt}=(1-q\frac{S_{j}}{N_{j}})\frac{c_{j}}{365}S_{j}-(a+)R_{j}$ (S11)

$$\frac{dP_{j}}{dt}=q\frac{S_{j}}{N_{j}}\frac{c_{j}}{365}S_{j}-\alpha P_{j}+\alpha\left( {1-}_{Y} \right)N_{j}$$

Here $\frac{c_{j}}{365}S_{j}=\frac{c_{j}}{365}\frac{S_{j}}{N_{j}}N_{j}$ represents pre-emptive vaccination which will successfully be given to susceptible people. The remaining $\frac{c_{j}}{365}(N_{j}-S_{j})$ vaccines given will not change anyone’s status and do not appear in the above equations, which are just wasted. Because of test in the 1^st^ visit, the second dose will be given only to the susceptible, i.e., $q\frac{S_{j}}{N_{j}}$. The total number of vaccines through the pre-emptive vaccination scenario during the five-year period is:

$\int_{0}^{5*365} \sum_{j=1}^{4} \frac{c_{j}}{365}\left( N_{j}+qS_{j}\left( t \right) \right)dt=5\left[ N_{2}c_{2}+N_{4}c_{4} \right]+q\int_{0}^{5*365} [\frac{c_{2}}{365}S_{2}\left( t \right)+\frac{c_{4}}{365}S_{4}\left( t \right)]dt$,

which is also dependent on the proportion of susceptible MSM. The total cost with vaccination scenario 3 is the sum of the following two costs: the cost involved with tests and 1^st^ dose of vaccine

$$5{\{{(c}_{2}N}_{2}+{c_{4}N}_{4})\left( T_{c}+V_{c}+A_{c}+rG_{c} \right)\},$$

and the cost involved with 2^nd^ dose of vaccine

$q(V_{c}+A_{c}+{r_{2}G}_{c})\int_{0}^{5*365} [\frac{c_{2}}{365}S_{2}\left( t \right)+\frac{c_{4}}{365}S_{4}\left( t \right)]dt$.

Here the full SHS clinic appointment cost (G_c_) for 2^nd^ dose of vaccine is removed (*r*_2_=0). The mean proportion of MSM who get 2 doses of vaccine among these vaccinated over a period of five years can be calculated as $q^{*}=(q\int_{0}^{5*365} \left[ \frac{c_{2}}{365}S_{2}\left( t \right)+\frac{c_{4}}{365}S_{4}\left( t \right) \right]dt)/[5\left( {c_{2}N}_{2}+{c_{4}N}_{4} \right)]$.

To distinguish the costs due to tests and vaccination, it is assumed that the costs due to tests is $5{\{{(c}_{2}N}_{2}+{c_{4}N}_{4})\left( T_{c}+{0.5(V}_{c}+rG_{c} \right))\}$and all the rest are due to vaccination.

*Simulation results*

Assuming a five-year non-outbreak period with pre-emptive vaccination occurring in SHS, Figure S2 shows how seroprevalence (or immunity), the number of vaccines required, and costs increase with yearly coverage rates (vaccination rate or testing rate if test first).


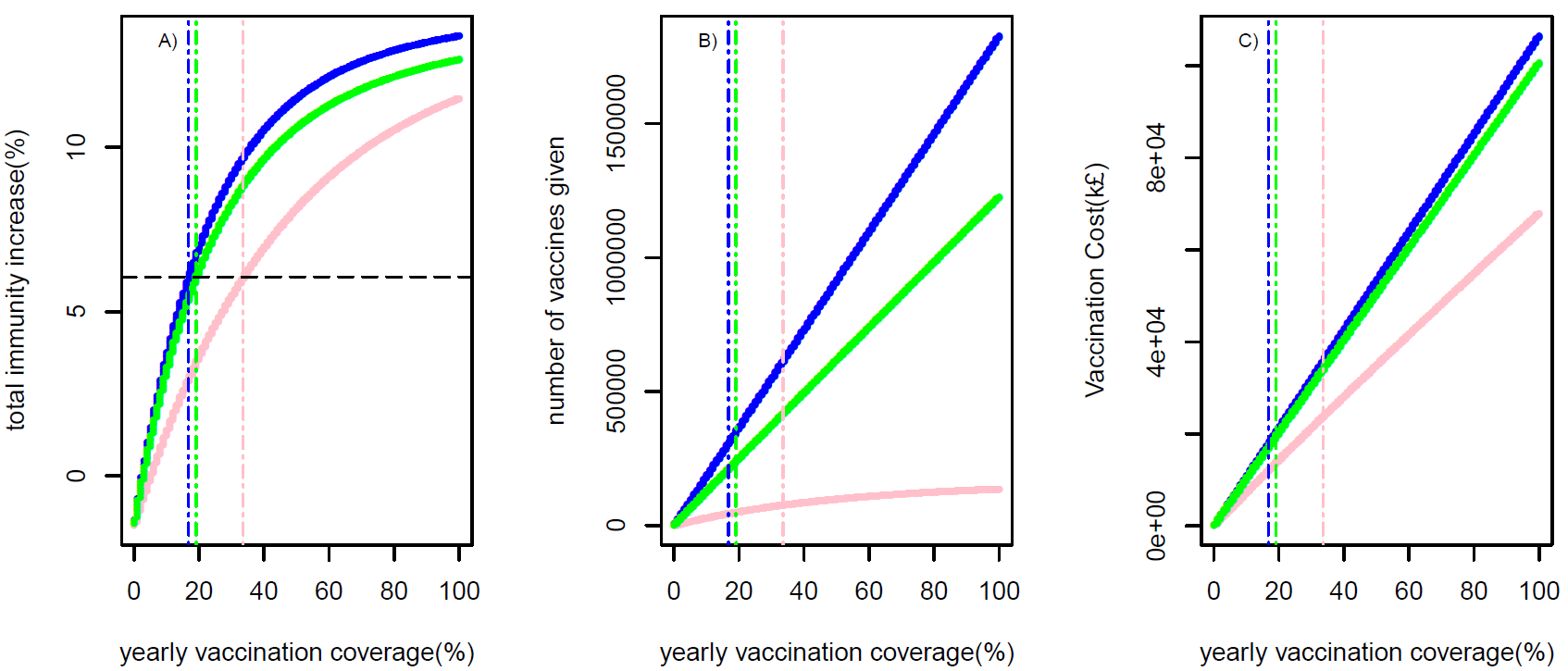


**Figure S2** **Effect on overall immunity/seroprevalence and cost for different yearly pre-emptive vaccination rates among MSM who attend SHS clinics over a five years’ non-outbreak period: change in overall immunity/seroprevalence (panel A), number of vaccines given over 5 years (panel B) and vaccination cost (panel C).** The colour blue, pink and green represent vaccination scenario 1, 2 and 3, respectively. The initial seroprevalence is set to 65.8% across four groups of MSM. The duration of immunity induced by one dose of vaccine is assumed to be 7 years. The vertical lines represent the yearly coverage rates (16.8%, 33.6%, 19.1%) required for scenarios 1, 2 and 3 to achieve an increase of 15% in immunity/seroprevalence among MSM who attend SHS clinics and the overall immunity change of 6.1% within the whole MSM population over 5 years. Note that for scenario 2, the “yearly coverage” is the yearly testing rate. For example, the mean yearly coverage required for achieving 15% increase in immunity of SHS attendee groups is 4.2% among the susceptible MSM

For a specific increase in seroprevalence/immunity among MSM attending SHS clinics, the results in Table S5 show that scenario 2 (test then vaccinate) requires the fewest vaccines, but scenario 1 (vaccinate without testing) is the cheapest strategy. We therefore consider scenario 1 going forward in the main analyses.

**Table S5** **Number of vaccines needed and cost of pre-emptive vaccination for increasing the seroprevalence or immunity level among MSM who attend SHS clinics by specific amounts over 5 years.** Outputs also include yearly coverage, number of vaccines required, and the total cost involved.

| **Vaccination scenario** | **Target increase in seroprevalence** | **Required yearly coverage of vaccination in SHS attendees; coverage of testing for scenario 2 in brackets** | **Number of vaccines needed over 5 years** | **Cost (1000s of GB£)** | **Proportion of costs due to tests** |
| --- | --- | --- | --- | --- | --- |
| 1 | 1% in SHS attendees; 0.39% in whole MSM | 1.79% | 32,658 | 1,902 | 0.0% |
| 2 |  | 0.63%(3.75%) | 11,536 | 2,740 | 87.4% |
| 3 |  | 1.96% | 25,306 | 2,013 | 43.0% |
| 1 | 5% in SHS attendees; 1.45% in whole MSM | 5.18% | 94,708 | 5,516 | 0.0% |
| 2 |  | 1.62% (10.36%) | 29,764 | 7,507 | 88.1% |
| 3 |  | 5.71% | 73,093 | 5,841 | 43.2% |
| 1 | 10% in SHS attendees; 3.75% in whole MSM | 10.18% | 186,151 | 10,843 | 0.0% |
| 2 |  | 2.91% (20.54%) | 53,350 | 14,714 | 89.1% |
| 3 |  | 11.43% | 144,935 | 11,646 | 43.3% |
| 1 | 15% in SHS attendees; 6.05% in whole MSM | 16.79% | 306,986 | 17,882 | 0.0% |
| 2 |  | 4.21% (33.57%) | 77,184 | 23,754 | 90.3% |
| 3 |  | 19.11% | 240,286 | 19,410 | 43.3% |

At the start, the immunity level of MSM is set to 65.8%, the seroprevalence after the 2016/18 outbreak. The duration of immunity derived by one dose of vaccine is 7 years. As pre-emptive vaccination is given via SHS clinics, non-SHS attendees will not be vaccinated during the 5-year non-outbreak period. The immunity level among non-SHS attendees decreases by 1.57%.

**SI.2.2. Reactive vaccination during an outbreak period**

Let the daily number of reported new cases be *C*(*t*) with *t*=1 representing the symptom onset date of the first case(s). Monitoring the total number of cases within a moving window of 3 months (here the cases includes both imported and local cases), once it exceeds a start threshold level (say 30 cases), the outbreak is signalled to start: the last day of the 3 months window is denoted as *t*^outbreak^ and the total number of cases on this window is denoted as *C*^start^ $=\sum_{t=1}^{t\mathrm{outbreak}} C(t)$.

We assume that upon an outbreak of Hepatitis A, pre-emptive vaccinations will be switched to the outbreak reactive vaccinations: V^SHS^ – vaccination through SHS clinics and V^PC^ – contact tracing vaccinations through primary care. Assume that reactive vaccinations start after day *t*^outbreak^, and are arranged as follows: before the start of outbreak

V^PC^_j_(*t*)=0, V^SHS^_j_(*t*) = 0, *t*=1,…, *t*^outbreak^, *j*=1,…,4 ;

at the first day of outbreak:

V^PC^_j_(*t*) = (*N*_j_/*N*)×κ^PC^×*C*^start^, *t*= t^outbreak^+1, *j*=1,…,4

V^SHS^_2_(*t*) = (*N*_2_/(*N*_2_+*N*_4_))×κ^SHS^×C^start^, V^SHS^_4_(t) = (*N*_4_/(*N*_2_+*N*_4_))×κ^SHS^×C^start^, *t*= t^outbreak^+1;

from the second day of the outbreak, vaccination responds to the reporting of daily number of cases of the previous day as

V^PC^_j_(*t*) = (*N*_j_/*N*)×κ^PC^×*C*(*t*-1), *t*= t^outbreak^+2,…, *j*=1,…,4

V^SHS^_2_(*t*) = (*N*_2_/(*N*_2_+*N*_4_))×κ^SHS^×*C*(*t*-1), V^SHS^_4_(*t*) = (*N*_4_/(*N*_2_+*N*_4_))×κ^SHS^×*C*(*t*-1), *t*= t^outbreak^+2,….

Here κ^SHS^ and κ^PC^ are the ratio of vaccines per case within SHS and primary care (Table S6 – at end), respectively.

When the effective reproductive number (i.e., *R*_e_=*ρR*_0_) is below the critical value 1.0, the number of infected persons will decrease. As there is a recruit rate α for the young men to enter to and the old men to exit from the MSM population, this increases the proportion of susceptible MSM because the young men are more likely to be susceptible than the old men (Table S3). When the pre-emptive vaccination is not strong enough, the proportion of the susceptible MSM will increase again and so will *R*_e_; as a result, periodic outbreaks can occur (See Figure 3). Taking this into account, we assume that the effective vaccination should be strong enough to avoid the periodic outbreaks.

**SI.3 Estimating health benefits and costs**

**SI.3.1 Health benefits**

To estimate the health benefit of vaccination characterised by QALYs gained for each scenario, occult infections were assumed to progress to either symptomatic, fulminant, liver transplant and asymptomatic cases, with the probabilities *p*_S_=84.1%, *p*_F_=0.37%, *p*_L_= 0.13% and 1-*p*_S_-*p*_F_-*p*_L_, respectively (see Figure 1). Two additional compartments *F* (fulminant cases) and *L*(liver transplant cases) are included in the cost-effectiveness model as shown in Figure 1B and differential equations. We assumed the duration of symptoms for outpatient and inpatient symptomatic HAV infections was *D*_out_=34.4 days and *D*_in_ =67.8 days, respectively, and so the average duration for symptomatic cases was *d*_S_ = 1/(*p*_in_/D_in_+*p*_out_/D_out_) with *p*_in_ = 63.7% being the proportion of symptomatic cases who admitted to hospitals (not including those with fulminant disease or liver transplant, see Table S6) and *p*_out_ = *1* -*p*_in_ being the proportion of symptomatic cases who were not admitted to a hospital. The assumption about the effective duration of symptoms for fulminant cases was *d*_F_ =67.8 days, and the duration of symptoms for liver transplant cases was *d*_L_ =153.2 days [13] while keeping the duration of infectiousness of asymptomatic cases at seven days (*d*_2_). The utility weights for healthy MSM, asymptomatic, symptomatic, fulminant and post liver transplant cases were UW_0_= 0.90, UW_A_= 0.83, UW_D_=0.64, UW_F_=0.26 and UW_L_=0.73 [13, 14], respectively. A sensitivity analysis of utility weight on the effectiveness analysis of vaccination strategy will be given in SI.4.13. The quality-adjusted life day gained over an outbreak of duration *D*_OTB_ is calculated as

$$QALDs={UW}_{0}\int_{0}^{D_{\mathrm{OTB}}} \sum_{j=1}^{4} \left( S_{j}\left( t \right)+E_{j}\left( t \right)+O_{j}\left( t \right)+R_{j}\left( t \right) \right)dt+{UW}_{A}\int_{0}^{D_{\mathrm{OTB}}} \sum_{j=1}^{4} A_{j}\left( t \right)dt+{UW}_{D}\int_{0}^{D_{\mathrm{OTB}}} \sum_{j=1}^{4} D_{j}\left( t \right)dt+{UW}_{F}\int_{0}^{D_{\mathrm{OTB}}} \sum_{j=1}^{4} F_{j}\left( t \right)dt+{UW}_{L}\int_{0}^{D_{\mathrm{OTB}}} \sum_{j=1}^{4} L_{j}\left( t \right)dt$$

To compare outbreaks under different situations, we consider a period of 10 years and the QALYs over this period should be calculated as:

QALYs= (QALDs+UW_0_×(10×365-D_OTB_) ×531559)/365.

Here 531559 is the size of MSM population in England.

**SI.3.2 Productivity losses**

Based on the Office for National Statistics (ONS) publications, the median weekly earnings for people aged 16-64 years old was 584.9 pounds for full-time jobs in 2019 [15]. The gender pay gap was 0.9%, 2.1%,1.9% and 11.4% for the people aged 18-21, 22-29, 30-39, 40-49 years old. Therefore, the naive average gender pay gap is 4.2%. This gives the male weekly earning as 585*1.021=597 pounds [15]. To convert to the UK currency of 2017 we account for inflation to give *W*_wk_=0.96*597= 573 pounds. The male employment rate in England 2020 is (*r*_e_=) 80.4% [16]. We assumed the duration of absenteeism from work was (A_out_=)15.5, (A_in_=)33.2, (A_F_=)33.2, and (A_L_=)153.2 days for outpatients, inpatients, fulminant patients and transplant patients, respectively [13]. The productivity losses (PL) over an outbreak of duration *D*_OTB_ was estimated by multiplying the number of these new patients by the number of days of their corresponding absenteeism,

$PL=r_{e}\frac{W_{wk}}{7}\int_{0}^{D_{\mathrm{OTB}}} \sum_{j=1}^{4} [D_{out,j}\left( t \right)A_{out}+D_{in,j}\left( t \right)A_{in}+F_{j}\left( t \right)A_{F}+L_{j}\left( t \right)A_{L}]dt$

Here the prefix Δ represents the corresponding daily number of new patients.

**SI.3.3 Costs of the outbreak**

The costs incurred by hepatitis A outbreaks were calculated by including three components: clinical case management (CCM), public health response (PHR) and coordination and training (CT) involved during the implementation of control measures. For an outbreak of a total size *M* and duration of *D*_OTB_, the total cost is

Cost__OTB_(*M*,*D*_OTB_) = Cost__CCM_(*M*)+ Cost__PHR_(*M*) + Cost__CT_(*M*)

The detail of each element and the cost parameters involved are listed in Table S6 (at end). If there are no outbreak control measures, the total cost during the outbreak is

Cost__OTB_(*M*,*D*_OTB_) = Cost__CCM_(*M*)

If reactive vaccination is launched but without CT (hence no reduction in contact rate induced), the total cost during the outbreak is

Cost__OTB_(*M*,*D*_OTB_) = Cost__CCM_(*M*)+ Cost__PHR_(*M*)

Following Dhankhar et el. [13], an annual 3% discount rate to QALYs and costs was used in the calculations.

**SI.3.4 Cost-effectiveness of vaccination strategies**

To assess whether vaccination strategy is cost-effective, we calculated the incremental cost-effectiveness ratio (ICER), which is defined as the incremental cost for control option A compared to a reference option B, divided by the incremental effectiveness (i.e. quality-adjusted life year (QALY) gained) of option A compared with option B.

ICER = (C_A_-C_B_)/(E_A_-E_B_)

Here *E* is its effectiveness and measured by QALYs saved, and *C* represents the total costs of an outbreak. Costs are reported in pounds (GB£) (currency of 2017).

**SI.3.5 Simulations**

Without reactive vaccination (RV), the modelled 2023 outbreak is periodic if pre-emptive vaccinations (PV) increase the immunity of SHS attendees by ≤8% over 5 years (Figure 3) (For convenience below, the PV rate is defined as X% if it increases the seroprevalence of SHS attendees by X% over 5 years). With RV alone, the modelled 2023 outbreak will end within 4.5 years with a size of 3770 cases. When combining both PV and RV vaccination together, periodic outbreaks will occur if PV rate is ≤ 6% (Figure 3). Some interesting phenomena are observed in Figure S3. For example, with PV alone, the outbreak size reduces when PV rate increases from 0% to 1%, and then it remains nearly unchanged from 2% to 5% (actually it slightly increases from 41,466 at PV rate of 4% to 41,785 at PV rate of 5%) (left panel of Figure S3). Intuitively, a continuing reduction is expected. This unusual event appears to be the consequence of interplay among immunity decay, immunity gained through an outbreak and PV vaccination. If immunity is assumed to remain unchanged over time (SI.4.6), then oscillating epidemics disappear and the outbreak size decreases with the PV rate.

With RV added, the duration of the outbreak increases as we increase the PV rate from 0% to 1%. This is a consequence of interaction between RV and continuing recruit of susceptible MSM (i.e., decaying immunity). In our analyses, we assume RV is proportional to the current number of cases. With a high initial proportion of susceptible MSM (with PV rate = 0% among SHS attendees), many individuals can be infected at an early stage; this will then induce strong RV which outnumbers the recruitment of the susceptible such that the immunity exceeds the herd immunity and the epidemic stops. With a lower initial proportion of susceptible MSM (such as if PV rates of 1-6%), the number of infected individuals is not high and quick enough to induce a large RV response that can outnumber the recruitment of new susceptibles and so herd immunity is not reached. With continuing recruitment of susceptible MSM, levels of immunity will oscillate and so does the number of infected MSM.

A further non-intuitive phenomenon is the decrease-increase-decrease-increase pattern of change in the total costs with an increasing PV rate (right panel of Figure S3). The weak increase in total costs around a PV rate of 4% is due to the combination of increases in PV cost, slow decreases in Cost due to CCM and PHR (the results of the nearly constant size of outbreak), and the unusual slight increase in Productivity losses (PL). The unexpected increase in PL when PV rate changes around 4% results from two effects: 1. When calculating PL, the PL of each patient is counted at its first day of illness rather than the end day of the absenteeism. The number of days off work due to illness was: 15.5, 33.2, 33.2, 153 for outpatients, inpatients, fulminant hepatitis patients and liver transplant patients respectively; and 2. Oscillating epidemics. With oscillating epidemics, the number of cases in the last week of a 10-year period is 10.8, 13.5 and 14.6 when PV rate increase from 3% to 5%. These patients reported on last week, and other patients with symptom onset within the 2 weeks (for outpatients) or one month (for inpatients or fulminant hepatitis patients) or 5 months (for liver transplant patients) before the last day of the 10-year period would have most or part of their PL occurring beyond the 10-year period (i.e., end effect because of oscillating epidemics). If we remove these contributions to PL, the unusual but small increase in PL should disappear. This is confirmed when we assume there is no decay in immunity (SI.4.6) or increasing the strength of RV (SI.4.11), oscillating epidemics disappear, and the increasing PL pattern vanishes. The end effect also reduces if we increase the discount rate to costs and QALYs. This unusual change pattern of total costs gives the strange pattern in the ICER. In this study, when calculating ICER to select the most cost-effective PV scenario, no PV rate was selected that resulted in oscillating epidemics based on the intuition that if a strategy cannot stop the spread of HAV, it cannot be regarded as effective.

If the initial seroprevalence is different from 65.8%, then the maximum pre-emptive vaccination coverage that is cost-effective is different as shown in Table S7.


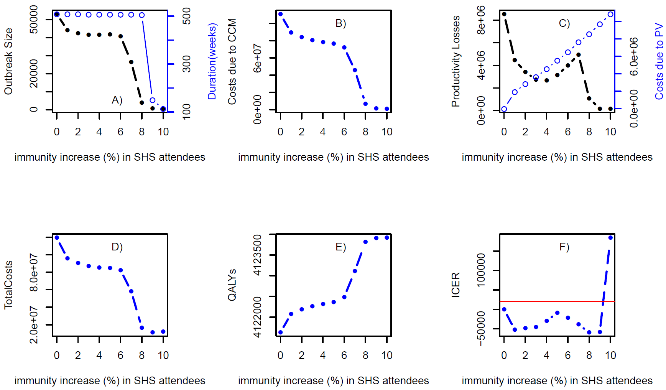

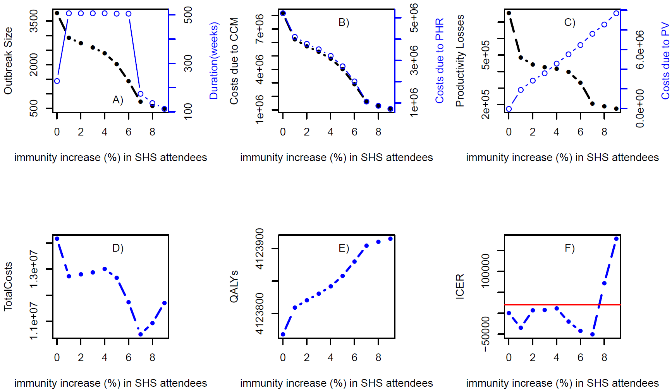
**Figure S3** **Effects of different increases in seroprevalence/immunity among SHS attenders due to pre-emptive vaccination without (left 6 figures) or with reactive vaccination (right 6 figures) on the proposed 2023 outbreak and its overall costs and QALYs**: A) outbreak size and duration; B) Costs due to Clinical Case Managements and Public Health Responses (if reactive vaccination too); C) Productivity Losses and Costs due to pre-emptive vaccination; D) Total costs, E) QALYs gained within 10 years, and F) ICER. Left side for pre-emptive vaccination alone and right side for Reactive vaccination added to Pre-emptive vaccination. Note that if the outbreak duration is about 10 years, it implies that the outbreak is oscillating.

**Table S7** **Maximum increases in seroprevalence in SHS attendees (with resulting increase in seroprevalence among all MSM in brackets) due to pre-emptive vaccination that is cost-effectiveness for different initial seroprevalences.**

| **Initial seroprevalence** | **Maximum increase (overall change) in seroprevalence among SHS attendees over a 5-year period** | **Yearly coverage of vaccination in SHS MSM** | **ICER compared to no vaccination** |
| --- | --- | --- | --- |
| 70.0% | 4.0%(0.69%) | 5.54% | -46,241 |
| 68.0% | 6.0% (1.76%) | 6.88% | -46,684 |
| 65.8% | 9.0% (3.29%) | 9.11% | -47,471 |
| 63.0% | 12.0% (4.87%) | 11.07% | -49,103 |
| 60.0% | 15.0% (6.47%) | 12.86% | -50,744 |

The increase in seroprevalence is among MSM who attend SHS clinics. Without pre-emptive vaccination, the reduction in seroprevalence during a 5-year period is 2.1%, 1.9%, 1.6%, 1.2% and 0.8% respectively for the initial seroprevalence 70.0%, 68.0%, 65.8%, 63% and 60%. Here we assume no outbreak control measures taken.

**SI.4 Sensitivity analyses**

The impacts of different sensitivity analyses on the transmission dynamics, costs and QALYs of the modelled outbreaks are summarised in Table S8, Table 3 (for PV rate =7% over 5 years), and Table S9 (for PV rate=9% over 5 years), respectively. In the below we will give the details of each model sensitivity analysis.

**Table S8 Effect of sensitivity analyses on the HAV transmission dynamics of the outbreak in 2023.** Here listed are the medians and their 95% credible interval (in brackets) of model parameters.

| Parameters | Baseline fitting to 725 male cases | Fitting to 507 MSM cases | Assortative mixing parameter (b=20%) | Heterogeneous immunity among groups (ratio 1:0.9:0.8:0.7) | Vaccination from April 2017 (same daily vaccines as 1 July) | Mild symptomatic cases transmit infection (proportion of mild cases: 20.5%) |
| --- | --- | --- | --- | --- | --- | --- |
| Transmission coefficient (*β*) per week | 1.48 [1.33,1.60] | 1.42 [1.27,1.60] | 1.48 [1.31,1.63] | 1.62 [1.48,1.78] | 141 [1.32,1.53] | 1.34  [1.28,1.44] |
| Initial susceptibility (*ρ*)^♦^ | 0.309 [0.284,0.341] | 0.310 [0.275,0.343] | 0.308 [0.280,0.348] | 0.306 [0.276,0.343] | 0.321 [0.295,0.345] | 0.307  [0.287,0.325] |
| Mean susceptibility after outbreak | 0.296 [0.272,0.327] | 0.299 [0.265,0.331] | 0.295 [0.268,0.334] | 0.268 [0.241,0.300] | 0.299 [0.275,0.322] | 0.292  [0.272,0.308] |
| Reduction in susceptibility due to outbreak | 0.013 [0.012,0.014] | 0.011 [0.010,0.012] | 0.013 [0.012,0.014] | 0.042 [0.038,0.047] | 0.022 [0.020,0.023] | 0.016  [0.015,0.017] |
| Time when sexual contact rate decreases (*t_c_*) - weeks | 48.7 [43.1,54.1] | 47.3 [41.1,53.4] | 48.4 [42.1,53.4] | 50.8 [45.0,57.3] | 50.5 [44.7,59.0] | 48.6  [42.2,54.3] |
| Reduction in sexual contact rate (1-*w*) after t_c_ | 0.169 [0.131,0.214] | 0.199 [0.141,0.261] | 0.166 [0.128,0.208] | 0.143 [0.102,0.194] | 0.129 [0.088,0.175] | 0.157  [0.116,0.201] |
| *R*_0_ before decrease in contact rate | 3.19 [2.87,3.46] | 3.06 [2.74,3.46] | 3.20 [2.82,3.52] | 3.50 [3.11,3.80] | 3.05 [2.85,3.30] | 3.18 [3.02,3.41] |
| *R*_e_ after decrease in contact rate | 0.82 [0.77,0.85] | 0.76 [0.70,0.81] | 0.82 [0.78,0.85] | 1.01 [0.95,1.05] | 0.85 [0.81,0.89] | 0.82 [0.78,0.86] |

The baseline scenario is defined by an outbreak start criterion defined as when there are >=30 cases reported within 3 month and ends when there is <=1 case within one month; the gap between outbreaks is 5 years; immunity decreases between outbreaks due to inflow of susceptible and outflow of immune MSM; mildly symptomatic MSM are not sexually active; weak assortative mixing (b=3.5%); equal initial immunity levels among 4 MSM sub-groups (high and low risk and SHS attendance or not). Baseline vaccination is to all SHS attendees equally with RV starting immediately once the outbreak starts (if RV is done) and no change in contact rate during the outbreak. Effect of 1^st^ vaccine dose is assumed to last 7 years and 50% return for second dose.

**Table S9 Sensitivity analysis on the costs (in millions of GB£), QALYs and incremental cost-effectiveness ratio (ICER in cost per QALY saved) of pre-emptive or reactive vaccination strategies, solely or in combination.** The ICERs of the single interventions are compared to a counterfactual of no vaccination, while the combined scenario considers the ICER of adding the reactive vaccination to the pre-emptive vaccination that increases seroprevalence among SHS attendees by 9% over 5 years.

| **Scenario** | **No Intervention** | | **Just pre-emptive vaccination of PV=9% compared to no intervention** | | | **Just reactive vaccination compared to no intervention** | | | **Both scenarios combined incremental to pre-emptive vaccination of PV=9%** | | |
| --- | --- | --- | --- | --- | --- | --- | --- | --- | --- | --- | --- |
|  | **Total Cost** | **Total QALYs** | **Incremental cost** | **Incremental QALYs** | **ICER** | **Incremental cost** | **Incremental QALYs** | **ICER** | **Incremental cost** | **Incremental QALYs** | **ICER** |
| Baseline* | 119.5 | 4,121,629 | -108.4 | 2,282.6 | CS | -105.3 | 2,138.8 | CS | 0.55 | 3.6 | 153,115 |
| RV delayed to 52 weeks into outbreak | 119.5 | 4,121,629 | -108.4 | 2,282.6 | CS | -100.9 | 2,091 | CS | 0.70 | 1.3 | 531,381 |
| Contact rate reduces by 16.9% from week 49 | 40.4 | 4,123,199 | -29.13 | 714.5 | CS | -29.67 | 616.5 | CS | 0.57 | 2.2 | 258,678 |
| Vaccination of just high-risk SHS attendees | 119.5 | 4,121,629 | -33.567 | 658.6 | CS | -109.2 | 2,185.8 | CS | -78.120 | 1595.8 | CS |
| Alternative outbreak criterion (50 cases over 3 months) | 119.4 | 4,121,631 | -108.27 | 2,281.1 | CS | -105.3 | 2,136.8 | CS | 0.54 | 3.3 | 163,705 |
| 10 year gap between outbreaks | 119.8 | 4,121,245 | -126.5 | 2,664.6 | CS | -124.5 | 2,491.3 | CS | 0.56 | 4.3 | 130,466 |
| Steady immunity between outbreaks | 49.9 | 4,122,941 | -41.1 | 968.0 | CS | -41.0 | 890.4 | CS | 0.52 | 5.0 | 105,265 |
| Moderate assortative mixing parameter (b=20%) | 122.7 | 4,121,582 | -111.5 | 2,327.9 | CS | -107.5 | 2,173.9 | CS | 0.53 | 4.6 | 113,760 |
| SHS attendees have higher immunity (1:0.9:0.8:0.7) | 109.4 | 4,121,800 | -96.819 | 2,116 | CS | -96.2 | 1,979.9 | CS | 5.3 | 1.6 | 332,377 |
| Mildly symptomatic MSM transmit HAV | 338.4 | 4,117,885 | -109.5 | 1904.5 | CS | -275.6 | 5,313.5 | CS | -179.3 | 3665.8 | CS |
| 5-year immunity for 1 dose vaccination | 119.5 | 4,121,629 | -107.8 | 2,282.6 | CS | -105.3 | 2,138.8 | CS | 0.55 | 3.6 | 153,115 |
| 75% return rate for 2^nd^ dose vaccination | 119.5 | 4,121,629 | -108.4 | 2,2282.6 | CS | -103.5 | 2,116.3 | CS | 0.58 | 3.3 | 179,874 |
| Doubling the strength of reactive vaccination (K^PC^=2×3.31, K^SHS^=2×34.41) | 119.5 | 4,121,629 | -108.4 | 2,282.6 | CS | -109.3 | 2,215.0 | CS | 0.86 | 5.8 | 149,988 |
| Utility weight reduced by 10% (absolute) for HAV-related states. Other health states remain at 0.90. | 119.5 | 4,121,629 | -108.4 | 3,168.3 | CS | -105.3 | 2,968.6 | CS | 0.55 | 5.0 | 110,313 |

CS denotes cost saving where the option is cheaper compared to the comparator and QALYs are gained. *: The baseline scenario is defined by an outbreak start criterion defined as when there are >=30 cases reported within 3 month and ends when there is <=1 case within one month; the gap between outbreaks is 5 years; immunity decreases between outbreaks due to inflow of susceptible and outflow of immune MSM; mildly symptomatic MSM are not sexually active; weak assortative mixing (b=3.5%); equal initial immunity levels among 4 MSM sub-groups (high and low risk and SHS attendance or not); the utility weights are: 0.83 for asymptomatic, 0.64 for symptomatic, 0.26 for fulminant, 0.73 for post liver transplant cases, and 0.90 for other health states. Baseline vaccination is to all SHS attendees equally with RV starting immediately once the outbreak starts (if RV is done) and no change in contact rate during the outbreak. Effect of 1^st^ vaccine dose is assumed to last 7 years and 50% return for second dose.

**SI.4.1 Outbreak only restricted to 507 MSM cases during 2016-18**

During the 2016/18 HAV outbreak, total number of HAV cases was 796 cases of which 71 were female. In the main text, transmission model was calibrated to 725 male cases among which 39 cases were imported. In this appendix, we refit the transmission model to the outbreak of 507 MSM cases among which 36 cases were imported. The estimates of model parameters are given in table S8. Other assumptions are the same as in Table 1. The results of Table 1 for the outbreak of 725 cases are also included for comparison. Table S8 shows that the differences in model parameter estimation for the two model fits are small.

**SI.4.2 Target pre-emptive and reactive vaccination strategies to high risk MSM who attend SHS clinics**

In the main text, we assume that V^SHS^ vaccination is given to both low and high-risk SHS attendees. Here we consider the V^SHS^ vaccination given only to the high-risk SHS attendees during both pre-emptive and reactive vaccination initiatives. The results are given in Table S10. Comparing with Table 2, we found that, to achieve a similar outcome, the strategies vaccinating only high-risk SHS MSM use less vaccines and save money than that vaccinating both low and high risk SHS MSM. For example, applying pre-emptive vaccination to limit the outbreak size around 571 cases, the strategy targeted to high risk SHS MSM required 129,923 vaccines while strategy targeted to both low and high risk SHS MSM required 166,556 vaccines. Their respective total costs are £11,149,000 and £9,015,000.

Comparing to Table 2, Table S10 shows that, with vaccination targeting only high risk SHS attendees, pre-emptive vaccination can be cost-effective (<£20,000/QALY) if it increases the seroprevalence among high risk MSM attending SHS by <18.0% over 5 years. This occurs if the yearly pre-outbreak vaccination coverage rate among high risk SHS attendees is <21.8%. As with the vaccination of all SHS attendees (Table 2), the optimal pre-emptive vaccination strategy saves more money and gains more QALYs than reactive vaccination, and therefore is more cost-effective. However, the combination of both vaccinations is also cost-effective if the pre-emptive vaccination increases the seroprevalence of high risk SHS attendees by less than 17% over 5 years.

**Table S10** **Cost-effectiveness analysis under pre-emptive vaccination (PV) and reactive vaccinations (RV) alone or together with PV and V^SHS^ of RV being targeted to high-risk SHS MSM only**. For PV among SHS attendees the cheapest scenario where just vaccinating without testing was assumed. No reduction in contact rate during the outbreak was assumed. The values for cost are in thousands of pounds and the values of ICER are in thousands of pounds per QALY.

| **Increase in seroprevalence among SHS attendees** | **Duration of outbreak (days)** | **Total cases during outbreak** | **Vaccination costs** | **Outbreak costs** | **Productivity losses** | **Total cos** | **QALYs** | **Incremental in comparing current scenario to previous scenario** | | | **Incremental in comparing current scenario to no vaccination** | | | **Incremental in comparing the combination of RV and PV to PV alone scenario** | | |
| --- | --- | --- | --- | --- | --- | --- | --- | --- | --- | --- | --- | --- | --- | --- | --- | --- |
|  |  |  |  |  |  |  |  | **costs** | **QALYs** | **ICER** | **costs** | **QALYs** | **ICER** | **costs** | **QALYs** | **ICER** |
| 1. **PV alone without control measures taken during the outbreak so the outbreak cost is the cost due to Clinical Case Management** | | | | | | | | | | | | | | | | |
| 0 | 3558^#^ | 52961 | 0 | 110977 | 8531 | 119508 | 4121629 |  |  |  |  |  |  |  |  |  |
| 0.01 | 3556^#^ | 47625 | 620 | 98430 | 6086 | 105137 | 4121891 | -14371 | 262 | CS | -14371 | 262 | CS |  |  |  |
| 0.05 | 3552^#^ | 43026 | 1799 | 85500 | 3364 | 90664 | 4122165 | -14472 | 274 | CS | -28843 | 536 | CS |  |  |  |
| 0.1 | 3547^#^ | 43024 | 3535 | 79074 | 3178 | 85789 | 4122310 | -4875 | 144 | CS | -33719 | 681 | CS |  |  |  |
| 0.11 | 3546^#^ | 43077 | 3970 | 77694 | 3643 | 85308 | 4122350 | -480 | 39 | CS | -34199 | 721 | CS |  |  |  |
| 0.12 | 3545^#^ | 41846 | 4404 | 74089 | 4185 | 82680 | 4122452 | -2628 | 102 | CS | -36828 | 823 | CS |  |  |  |
| 0.13 | 3544^#^ | 36267 | 4838 | 63227 | 4780 | 72846 | 4122731 | -9833 | 278 | CS | -46661 | 1102 | CS |  |  |  |
| 0.14 | 3543^#^ | 21949 | 5334 | 38067 | 4830 | 48232 | 4123271 | -24614 | 539 | CS | -71276 | 1642 | CS |  |  |  |
| 0.15 | 3542^#^ | 7289 | 5831 | 12873 | 2036 | 20741 | 4123719 | -27490 | 448 | CS | -98766 | 2091 | CS |  |  |  |
| 0.16 | 3541^#^ | 1973 | 6327 | 3737 | 552 | 10617 | 4123869 | -10124 | 149 | CS | -108890 | 2240 | CS |  |  |  |
| 0.17 | 1133 | 642 | 6947 | 1418 | 199 | 8565 | 4123908 | -2052 | 38 | CS | -110943 | 2279 | CS |  |  |  |
| 0.18 | 935 | 567 | 7568 | 1256 | 191 | 9015 | 4123911 | 450 | 3.362 | 134 | -110492 | 2283 | CS |  |  |  |
| 1. **RV via SHS clinics and primary care given (proportional to the daily incidence) once the outbreak is signalled to have started, and the outbreak cost is the sum of costs due to Clinical Case Management (CCM) and Public Health Response (PHR)** | | | | | | | | | | | | | | | | |
| RV alone | 1423 | 2717 | 0 | 5923 | 601 | 10323 | 4123815 |  |  |  | -109185 | 2186 | CS | -109185 | 2186 | CS |
| 0.01+RV | 1569 | 2337 | 620 | 5089 | 486 | 9481 | 4123832 | -843 | 17 | CS | -110028 | 2203 | CS | -95656 | 1941 | CS |
| 0.05+RV | 1940 | 1755 | 1799 | 3817 | 355 | 8481 | 4123858 | -1000 | 26 | CS | -111028 | 2230 | CS | -82184 | 1693 | CS |
| 0.10+RV | 1615 | 1075 | 3536 | 2350 | 261 | 7720 | 4123889 | -761 | 30 | CS | -111789 | 2260 | CS | -78069 | 1579 | CS |
| 0.11+RV | 1432 | 961 | 3970 | 2108 | 248 | 7735 | 4123894 | 15 | 5 | 3 | -111773 | 2265 | CS | -77573 | 1544 | CS |
| 0.12+RV | 1278 | 862 | 4404 | 1895 | 237 | 7801 | 4123898 | 66 | 4 | 15 | -111708 | 2270 | CS | -74879 | 1446 | CS |
| 0.13+RV | 1147 | 775 | 4839 | 1709 | 226 | 7914 | 4123902 | 113 | 4 | 29 | -111595 | 2273 | CS | -64933 | 1171 | CS |
| 0.14+RV | 1035 | 700 | 5335 | 1547 | 217 | 8130 | 4123906 | 216 | 3 | 64 | -111378 | 2277 | CS | -40102 | 634 | CS |
| 0.15+RV | 940 | 636 | 5831 | 1406 | 208 | 8384 | 4123908 | 253 | 3 | 88 | -111125 | 2280 | CS | -12358 | 188 | CS |
| 0.16+RV | 858 | 581 | 6328 | 1286 | 200 | 8672 | 4123911 | 288 | 2 | 116 | -110836 | 2282 | CS | -1946 | 42 | CS |
| 0.17+RV | 788 | 533 | 6948 | 1184 | 193 | 9114 | 4123913 | 441 | 2 | 205 | -110395 | 2284 | CS | 548 | 5 | 112 |
| 0.18+RV | 726 | 492 | 7568 | 1094 | 186 | 9577 | 4123915 | 464 | 2 | 248 | -109931 | 2286 | CS | 561 | 3 | 164 |

CS denotes cost saving where the option is cheaper compared to the comparator and QALYs are gained. ^#^: For the situations with pre-emptive vaccination that increase the seroprevalence of MSM who attend SHS by less than 17% over 5 years, or the situations with reactive vaccination added to pre-emptive vaccination that increases the seroprevalence of MSM who attend SHS by 6% to 8% over 5 years (not shown), the outbreak is periodic with a long period. Estimates listed are for the first 10 years.

Without pre-emptive vaccination, the seroprevalence or immunity level reduces from 65.8% to 64.2% at the end of a 5-year period. The incoming outbreak is assumed to be induced by importation of infections as for the 2016/18 outbreak.

**SI.4.3 Effect of the outbreak criterion**

In the main text, we assume that an outbreak is started when 30 cases over 3 months are reported and stops when there are less than 1 cases within one month, denoted as (30,1). Here we compare this outbreak criterion with another one: start when 50 cases over 3 months and stop when less than 3 cases per month, denoted as (50,3). The results listed in Table S11 show that for these situations where the outbreak starts and ends in accordance with the new criterion (50,3), the size and duration of the outbreak are shortened. Compared to undertaking pre-emptive vaccination under outbreak criterion (30,1) (Table 2), under the criterion (50,3) pre-emptive vaccination is cost-effective if it increases the seroprevalence of MSM who attend SHS clinics by ≤8% instead of ≤9% over 5 years. The different criteria do not change the results of cost-effectiveness analyses.

**Table S11** **Cost-effectiveness analysis under pre-emptive vaccination (PV) and reactive vaccination (RV) alone or in combination under the outbreak criterion of (50,3): start when 50 cases over 3 months and stop when less than 3 cases per month**. No reduction in contact rate during the outbreak was assumed. Costs are in thousands of GB£ and the values of ICER are in thousands of pounds per QALY.

| Increase in seroprevalence among SHS attendees | Duration of outbreak (days) | Total cases during outbreak | Vaccination costs | Outbreak costs | Productivity losses | Total cost | QALYs | Incremental in comparing current scenario to previous scenario | | | Incremental in comparing current scenario to no vaccination | | | Incremental in comparing the combination of RV and PV to PV alone scenario | | |
| --- | --- | --- | --- | --- | --- | --- | --- | --- | --- | --- | --- | --- | --- | --- | --- | --- |
|  |  |  |  |  |  |  |  | Costs | QALYs | ICER | Costs | QALYs | ICER | Costs | QALYs | ICER |
| **Pre-emptive vaccination alone without control measures taken during the outbreak, with the outbreak cost only including costs due to Clinical Case Management** | | | | | | | | | | | | | | | | |
| 0 | 2804 | 52884 | 0 | 110852 | 8529 | 119381 | 4121631 |  |  |  |  |  |  |  |  |  |
| 0.01 | 3527^#^ | 44104 | 1902 | 89625 | 4478 | 96006 | 4122077 | -23375 | 446 | CS | -23375 | 446 | CS |  |  |  |
| 0.05 | 3516^#^ | 41779 | 5517 | 76581 | 3148 | 85246 | 4122362 | -10759 | 286 | CS | -34135 | 731 | CS |  |  |  |
| 0.06 | 3513^#^ | 40666 | 6468 | 72112 | 3996 | 82577 | 4122488 | -2670 | 126 | CS | -36804 | 857 | CS |  |  |  |
| 0.07 | 3510^#^ | 26456 | 7610 | 45880 | 4948 | 58437 | 4123111 | -24140 | 623 | CS | -60944 | 1480 | CS |  |  |  |
| 0.08 | 1030 | 722 | 8561 | 1602 | 196 | 10358 | 4123905 | -48079 | 793 | CS | -109023 | 2273 | CS |  |  |  |
| 0.09 | 703 | 547 | 9702 | 1228 | 180 | 11110 | 4123912 | 752 | 8 | 96 | -108271 | 2281 | CS |  |  |  |
| 0.10 | 556 | 446 | 10844 | 1008 | 167 | 12018 | 4123917 | 908 | 5 | 197 | -107363 | 2286 | CS |  |  |  |
| 0.11 | 464 | 377 | 12175 | 859 | 155 | 13190 | 4123920 | 1171 | 3 | 369 | -106191 | 2289 | CS |  |  |  |
| **Reactive vaccination alone or with pre-emptive vaccination, with the outbreak cost including costs due to Clinical Case Management and Public Health Response** | | | | | | | | | | | | | | | | |
| RV alone | 1284 | 3756 | 0 | 13354 | 757 | 14111 | 4123768 |  |  |  | -105269 | 2137 | CS | -105269 | 2137 | CS |
| 0.01+RV | 1588 | 2699 | 1902 | 9605 | 458 | 11965 | 4123816 | -2146 | 48 | CS | -107415 | 2185 | CS | -84040 | 1739 | CS |
| 0.05+RV | 1399 | 1164 | 5517 | 4227 | 251 | 9995 | 4123885 | -1971 | 69 | CS | -109386 | 2254 | CS | -75251 | 1522 | CS |
| 0.06+RV | 1051 | 889 | 6468 | 3254 | 226 | 9949 | 4123897 | -46 | 12 | CS | -109432 | 2266 | CS | -72628 | 1409 | CS |
| 0.07+RV | 826 | 701 | 7610 | 2581 | 206 | 10397 | 4123906 | 448 | 8 | 53 | -108984 | 2274 | CS | -48040 | 794 | CS |
| 0.08+RV | 673 | 570 | 8561 | 2109 | 190 | 10860 | 4123911 | 463 | 6 | 78 | -108521 | 2280 | CS | 502 | 7 | 73 |
| 0.09+RV | 564 | 476 | 9702 | 1770 | 175 | 11647 | 4123916 | 787 | 4 | 184 | -107734 | 2284 | CS | 537 | 3 | 164 |
| 0.10+RV | 483 | 406 | 10844 | 1521 | 163 | 12527 | 4123919 | 880 | 3 | 275 | -106854 | 2288 | CS | 509 | 2 | 272 |
| 0.11+RV | 421 | 353 | 12175 | 1325 | 152 | 13652 | 4123921 | 1125 | 2 | 456 | -105728 | 2290 | CS | 463 | 1 | 398 |

CS denotes cost saving where the option is cheaper compared to the comparator and QALYs are gained. ^#^_:_ For the situations with pre-emptive vaccination that increases the seroprevalence of MSM who attend SHS from 1% to 7% over 5 years, or the situations with reactive vaccination added to pre-emptive vaccination that increases the seroprevalence of MSM who attendees SHS by the range from 3% to 4% over 5 years (not shown here), the outbreak is periodic with a long period. Estimates listed are for the first 10 years. Without pre-emptive vaccination, the seroprevalence or immunity level reduces from 65.8% to 64.2% at the end of a 5-year period. The incoming outbreak is assumed to be induced by importation of infections as for the 2016/18 outbreak.

**SI.4.4 Effect of late start of reactive vaccination**

In the main text, the reactive vaccination is assumed to start once the outbreak is signalled to have started. Here we investigate how different start times of reactive vaccination affect the outbreak and the cost-effectiveness of vaccination. We consider four initial overall seroprevalence at the modelled 2023 outbreak: 64.2%, 66.3%, 68.2% and 69.1% due to the pre-emptive vaccinations that increase the seroprevalence of SHS attendees by 0%, 3%, 7% and 9% respectively during a 5-year non-outbreak period. Reactive vaccinations are started at six times along the outbreak: 0, 13, 26, 39, 53, 65 weeks from the start of the outbreak. The results are given in Figure S4 and Table S12.

Under the situation of no pre-emptive vaccination, periodic outbreaks emerge if there is no RV. With RV starting at different times, the outbreak stops within 4.4 years. The number of cases increases but the duration of outbreak shortens with the delay of RV. The results in Table S12 show that the earlier RV starts, the more money and more QALYS saved. However, the difference between immediately starting RV or delaying by about 3 months doesn’t make any noticeable difference in total costs and QALYS saved.

Under pre-emptive vaccination that result in the seroprevalence of SHS attendees increasing by 1-7% over 5 years, the outbreak oscillates if there is no RV (Figure 3). Periodic outbreaks still emerge if RV starts early; however, the periodicity is avoided if RV starts late. For example, if PV rate =3%, the outbreak stops within 4.6 years if RV starts one year late (52 weeks). The results in Table S12 show that starting RV one year late saves most money and gains most QALYs comparing to no control at all. This implies that under these situations, the early RV might not be a good choice. However, under the situation of strong pre-emptive vaccination (9%) so that the outbreak stops within 10 years, the impact of RV improves as it is started closer to the outbreak start.


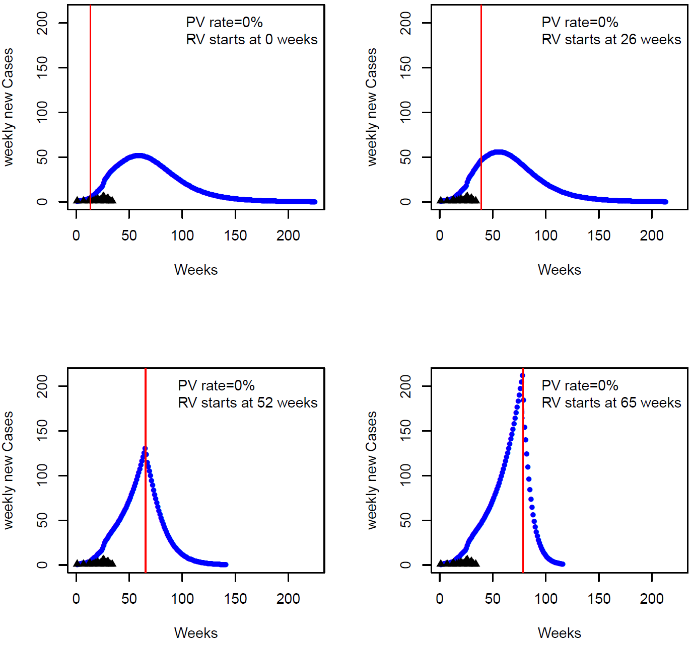

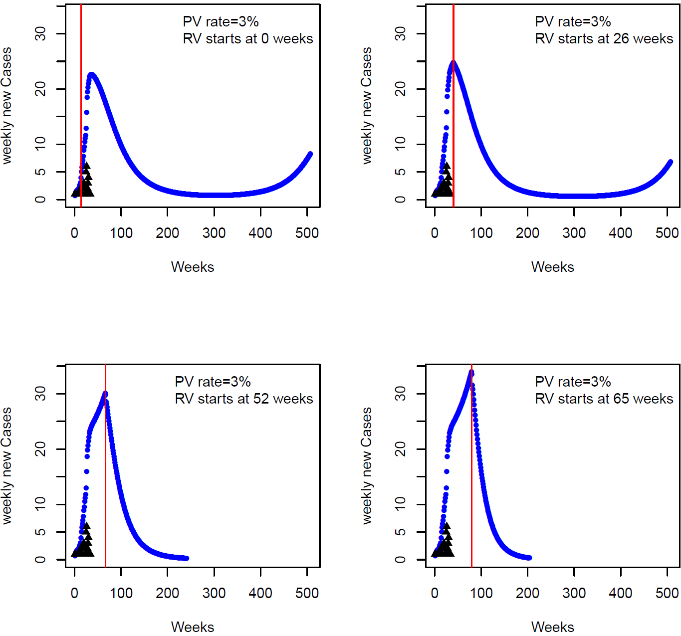

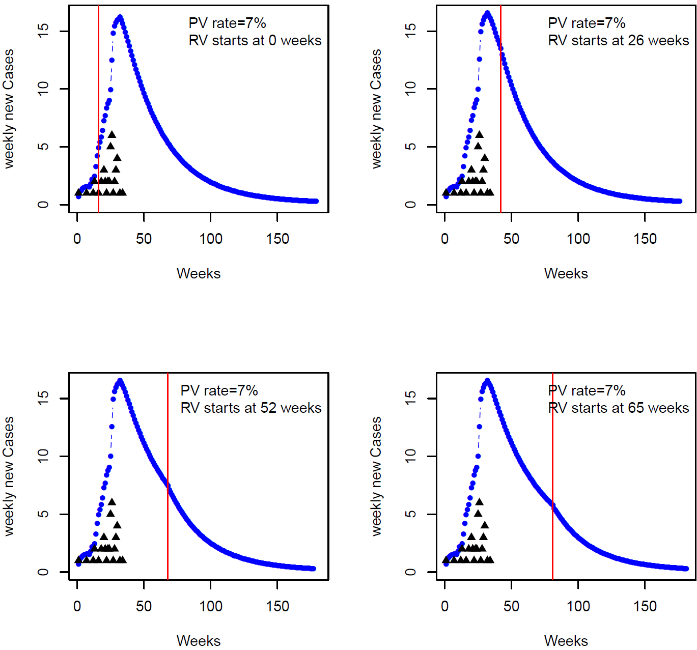

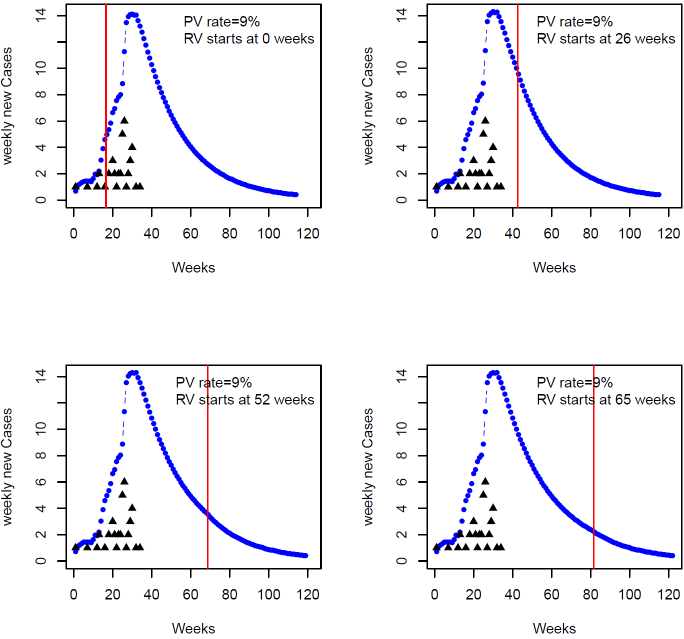


**Figure S4** **Effect of timing of reactive vaccinations with different levels of pre-emptive vaccination on the proposed 2023 outbreaks.** Six different pre-emptive vaccination rates are considered (0%, 3%, 7%, and 9%) and four start times of reactive vaccinations (0, 26, 52, 65 weeks after the start of the outbreak), with these being marked by vertical red lines. Other model parameters are the same as the baseline situation.

**Table S12** **Effects of start time of reactive vaccination on the proposed 2023 outbreak and impact of vaccination.** Under six initial seroprevalences, cost-effectiveness analyses were performed for three comparisons. No reduction in contact rate during the outbreak was assumed. All other parameters are the same as the baseline situation. Costs are in thousands of GB£ and the values of ICER are in thousands of pounds per QALY.

| Start time of Reactive vaccination (from outbreak start) | Duration of outbreak (days) | Total cases during outbreak | Vaccination costs | Outbreak costs | Productivity losses | Total cost | QALYs | Incremental in comparing current scenario to previous scenario | | | Incremental in comparing current scenario to no vaccination | | | Incremental in comparing the combination of RV and PV to no reactive vaccination | | |
| --- | --- | --- | --- | --- | --- | --- | --- | --- | --- | --- | --- | --- | --- | --- | --- | --- |
|  |  |  |  |  |  |  |  | costs | QALYs | ICER | costs | QALYs | ICER | costs | QALYs | ICER |
| PV rate =0% (Increase in seroprevalence among SHS attendees) initial overall seroprevalence =64.2% | | | | | | | | | | | | | | | | |
| (No vaccination | 3558^#^ | 52961 | 0 | 110978 | 8531 | 119509 | 4121629 |  |  |  |  |  |  |  |  |  |
| 65 weeks | 816 | 6531 | 0 | 23055 | 2560 | 25615 | 4123643 | -93893 | 2014.3 | CS | -93893 | 2014.3 | CS | -93893 | 2014.3 | CS |
| 52 weeks | 993 | 4802 | 0 | 17019 | 1608 | 18627 | 4123720 | -6987 | 76.8 | CS | -100881 | 2091.2 | CS | -100881 | 2091.2 | CS |
| 39 weeks | 1270 | 4049 | 0 | 14384 | 1061 | 15445 | 4123754 | -3182 | 34.2 | CS | -104063 | 2125.4 | CS | -104063 | 2125.4 | CS |
| 26 weeks | 1497 | 3842 | 0 | 13641 | 826 | 14466 | 4123764 | -978 | 9.8 | CS | -105042 | 2135.2 | CS | -105042 | 2135.2 | CS |
| 13 weeks | 1575 | 3815 | 0 | 13530 | 772 | 14302 | 4123765 | -164.5 | 1.5 | CS | -105206 | 2136.7 | CS | -105206 | 2136.7 | CS |
| 0 weeks | 1579 | 3817 | 0 | 13537 | 770 | 14308 | 4123765 | 5.9 | -0.1 | CS | -105200 | 2136.6 | CS | -105200 | 2136.6 | CS |
| PV rate=3% initial overall seroprevalence = 66.3% | | | | | | | | | | | | | | | | |
| (No RV) | 3549^#^ | 41579 | 3615 | 80848 | 2745 | 87208 | 4122264 |  |  |  | -32300 | 635.3 | CS |  |  |  |
| 65 | 1431 | 2482 | 3615 | 8791 | 494 | 12900 | 4123826 | -74308.1 | 1561.6 | CS | -106608 | 2196.9 | CS | -74308 | 1562 | CS |
| 52 | 1688 | 2221 | 3615 | 7899 | 441 | 11954 | 4123837 | -945.7 | 11.7 | CS | -107554 | 2208.6 | CS | -75254 | 1573 | CS |
| 39 | 3549^#^ | 2320 | 3615 | 8162 | 439 | 12215 | 4123837 | 261.5 | -0.7 | CS | -107293 | 2207.9 | CS | -74992 | 1573 | CS |
| 26 | 3549^#^ | 2493 | 3615 | 8584 | 437 | 12636 | 4123833 | 420.2 | -4.1 | CS | -106872 | 2203.8 | CS | -74572 | 1569 | CS |
| 13 | 3549^#^ | 2596 | 3615 | 8841 | 428 | 12884 | 4123830 | 247.9 | -2.9 | CS | -106625 | 2200.9 | CS | -74324 | 1566 | CS |
| 0 | 3549^#^ | 2605 | 3615 | 8869 | 427 | 12911 | 4123829 | 27.2 | -0.3 | CS | -106597 | 2200.6 | CS | -74297 | 1565 | CS |
| PV rate=5% initial overall seroprevalence = 67.2% | | | | | | | | | | | | | | | | |
| (No RV) | 3544^#^ | 41785 | 5517 | 76581 | 3148 | 85246 | 4122362 |  |  |  | -34262 | 733.7 | CS |  |  |  |
| 65 | 1708 | 1516 | 5517 | 5410 | 273 | 11200 | 4123869 | -74045.8 | 1506.7 | CS | -108308 | 2240.4 | CS | -74046 | 1507 | CS |
| 52 | 3544^#^ | 1658 | 5517 | 5847 | 320 | 11684 | 4123866 | 484.0 | -2.9 | CS | -107824 | 2237.4 | CS | -73562 | 1504 | CS |
| 39 | 3544^#^ | 1772 | 5517 | 6107 | 361 | 11985 | 4123864 | 300.2 | -2.1 | CS | -107523 | 2235.3 | CS | -73262 | 1502 | CS |
| 26 | 3544^#^ | 1936 | 5517 | 6519 | 394 | 12430 | 4123860 | 445.4 | -4.2 | CS | -107078 | 2231.2 | CS | -72816 | 1497 | CS |
| 13 | 3544^#^ | 2052 | 5517 | 6825 | 401 | 12743 | 4123857 | 312.8 | -3.3 | CS | -106765 | 2227.9 | CS | -72503 | 1494 | CS |
| 0 | 3544^#^ | 2068 | 5517 | 6862 | 401 | 12781 | 4123856 | 37.8 | -0.5 | CS | -106727 | 2227.4 | CS | -72466 | 1494 | CS |
| PV rate =9% initial overall seroprevalence = 69.1% | | | | | | | | | | | | | | | | |
| (No RV) | 1054 | 571 | 9702 | 1267 | 180 | 11149 | 4123911 |  |  |  | -108359.5 | 2282.2 | CS |  |  |  |
| 65 | 858 | 551 | 9702 | 1992 | 180 | 11875 | 4123912.3 | 725.7 | 1.3 | 548.0 | -107633.8 | 2283.6 | CS | 725.728 | 1.324 | 548.0 |
| 52 | 835 | 542 | 9702 | 1966 | 180 | 11848 | 4123912.7 | -26.4 | 0.4 | CS | -107660.2 | 2284.0 | CS | 699.348 | 1.716 | 407.6 |
| 39 | 817 | 530 | 9702 | 1926 | 180 | 11808 | 4123913.2 | -39.6 | 0.5 | CS | -107699.8 | 2284.5 | CS | 659.711 | 2.252 | 293.0 |
| 26 | 805 | 515 | 9702 | 1873 | 180 | 11755 | 4123913.9 | -53.6 | 0.7 | CS | -107753.4 | 2285.2 | CS | 606.079 | 2.943 | 205.9 |
| 13 | 801 | 500 | 9702 | 1824 | 177 | 11703 | 4123914.6 | -52.1 | 0.7 | CS | -107805.5 | 2285.8 | CS | 554.014 | 3.605 | 153.7 |
| 0 | 801 | 498 | 9702 | 1813 | 176 | 11691 | 4123914.7 | -11.8 | 0.1 | CS | -107817.3 | 2286.0 | CS | 542.203 | 3.722 | 145.7 |

CS denotes cost saving where the option is cheaper compared to the comparator and QALYs are gained. ^#^For these situations, the outbreak is periodic with a long period. Estimates listed are for the first 10 years.

**SI.4.5 The effect of different non-outbreak periods**

In the main text for pre-emptive vaccination, a five years’ non-outbreak period is assumed. In Table S13 the effect of different lengths of non-outbreak period is shown by comparing three different periods: 3, 5, and 10 years. The results suggest that the longer the gap between the outbreaks, the higher the coverage and more vaccinations will be required to achieve the same target increase in immunity level. The reason is that the vaccination is assumed to be given only to SHS attendees, and so the longer the non-outbreak period, the more deterioration of immunity occurs among non SHS attendees.

**Table S13** **Impact of duration of non-outbreak gap on required pre-emptive vaccination coverage and cost for increasing the immunity level among MSM attending SHS clinics by different amounts, and effect on overall seroprevalence in all MSM**

1. Effect of length of the non-outbreak period under the target increase in the immunity of SHS attendees: 5%

| **Non-outbreak period (years)** | **Decrease in the immunity of non SHS attendees** | **Net change in the immunity of the whole MSM** | **Yearly coverage required** | **Number of vaccines needed** | **Cost (1000s pounds)** |
| --- | --- | --- | --- | --- | --- |
| 3 | 1.0% | 1.78% | 7.32% | 80,339 | 4,679 |
| 5 | 1.57% | 1.45% | 5.18% | 94,708 | 5,516 |
| 10 | 2.92% | -0.72% | 3.73% | 137,164 | 7,990 |

1. Effect of length of the non-outbreak period under the target increase in the immunity of the whole MSM: 1.45%

| **Non-outbreak period (years)** | **Decrease in the immunity of non SHS attendees** | **Increase in the immunity of the SHS attendees required** | **Yearly coverage required** | **Number of vaccines needed** | **Cost (1000s pounds)** |
| --- | --- | --- | --- | --- | --- |
| 3 | 0.97% | 4.3% | 6.25% | 68,582 | 3,995 |
| 5 | 1.57% | 5.0% | 5.18% | 94,708 | 5,516 |
| 10 | 2.93% | 6.6% | 4.64% | 169,822 | 9,892 |

We also considered how a ten-year gap between outbreaks affects the outcome of vaccination strategies. Simulation results are given in Table S14. Comparing to Table 2, Table S14 shows that under a 10-year gap between outbreaks, pre-emptive vaccination can be cost-effective (<£20,000/QALY) if it increases the seroprevalence among MSM attending SHS by <10.0% over 10 years. This occurs if the yearly pre-outbreak vaccination coverage rate among SHS attendees is <7.0%. Table S14 shows that under the 10-year gap between outbreaks, reactive vaccination alone is also cost-effective, but less so than the pre-emptive vaccination. Reactive vaccination added to a pre-emptive vaccination is cost-effective if the pre-emptive vaccination increases the seroprevalence of the SHS attendees by <9% over 10 years.

**Table S14** **Cost-effectiveness analysis under pre-emptive vaccination (PV) and reactive vaccination (RV) alone or in combination when the gap between outbreaks is 10 years.** No reduction in contact rate during the outbreak was assumed. Costs are in thousands of GB£ and the values of ICER are in thousands of pounds per QALY

| Increase in seroprevalence among SHS attendees | Duration of outbreak (days) | Total cases during outbreak | Vaccination costs | Outbreak costs | Productivity losses | Total cos | QALYs | Incremental in comparing current scenario to previous scenario | | | Incremental in comparing current scenario to no vaccination | | | Incremental in comparing the combination of RV and PV to PV alone scenario | | |
| --- | --- | --- | --- | --- | --- | --- | --- | --- | --- | --- | --- | --- | --- | --- | --- | --- |
|  |  |  |  |  |  |  |  | costs | QALYs | ICER | costs | QALYs | ICER | costs | QALYs | ICER |
| **Pre-emptive vaccination alone** **without control measures taken during the outbreak, with the outbreak cost only including costs due to Clinical Case Management** | | | | | | | | | | | | | | | | |
| 0 (baseline) | 2385 | 61174 | 0 | 129410 | 11981 | 141391 | 4121245 |  |  |  |  |  |  |  |  |  |
| 0.01 | 3554 | 45312 | 3424 | 92415 | 4824 | 100663 | 4122018 | -40728 | 773 | CS | -40728 | 773 | CS |  |  |  |
| 0.05 | 3545 | 42333 | 7990 | 78143 | 3045 | 89178 | 4122328 | -11485 | 309 | CS | -52213 | 1083 | CS |  |  |  |
| 0.06 | 3543 | 41922 | 9131 | 74855 | 3848 | 87834 | 4122420 | -1343 | 92 | CS | -53556 | 1175 | CS |  |  |  |
| 0.07 | 3541 | 32289 | 10653 | 56114 | 4845 | 71613 | 4122892 | -16222 | 472 | CS | -69778 | 1647 | CS |  |  |  |
| 0.08 | 3539 | 6552 | 11795 | 11589 | 1837 | 25221 | 4123742 | -46391 | 850 | CS | -116169 | 2497 | CS |  |  |  |
| 0.09 | 1161 | 614 | 13317 | 1356 | 186 | 14859 | 4123909 | -10363 | 168 | CS | -126532 | 2665 | CS |  |  |  |
| 0.10 | 843 | 492 | 14839 | 1094 | 172 | 16105 | 4123915 | 1246 | 5 | 227 | -125285 | 2670 | CS |  |  |  |
| **Reactive vaccination alone or with pre-emptive vaccination**, **with the outbreak cost including costs due to Clinical Case Management and Public Health Response** | | | | | | | | | | | | | | | | |
| RV alone | 1398 | 4455 | 0 | 15849 | 1004 | 16853 | 4123736 |  |  |  | -124537 | 2491 | CS | -124537 | 2491 | CS |
| 0.01+RV | 3554 | 2998 | 3424 | 10634 | 506 | 14565 | 4123805 | -2289 | 69 | CS | -126826 | 2560 | CS | -86098 | 1787 | CS |
| 0.05+RV | 3545 | 2125 | 7990 | 7095 | 408 | 15493 | 4123854 | 928 | 49 | 19 | -125898 | 2609 | CS | -73685 | 1526 | CS |
| 0.06+RV | 3543 | 1572 | 9131 | 5349 | 353 | 14834 | 4123875 | -658 | 21 | CS | -126556 | 2630 | CS | -73000 | 1455 | CS |
| 0.07+RV | 1307 | 773 | 10653 | 2845 | 213 | 13711 | 4123902 | -1123 | 27 | CS | -127680 | 2657 | CS | -57902 | 1011 | CS |
| 0.08+RV | 1005 | 623 | 11795 | 2303 | 196 | 14293 | 4123909 | 582 | 7 | 87 | -127098 | 2664 | CS | -10928 | 167 | CS |
| 0.09+RV | 824 | 518 | 13317 | 1923 | 181 | 15420 | 4123914 | 1127 | 5 | 238 | -125970 | 2669 | CS | 562 | 4 | 130 |
| 0.10+RV | 700 | 441 | 14839 | 1639 | 168 | 16646 | 4123917 | 1226 | 3 | 350 | -124745 | 2672 | CS | 541 | 2 | 234 |

CS denotes cost saving where the option is cheaper compared to the comparator and QALYs are gained. ^#^_:_ For the situations with pre-emptive vaccination that increases the seroprevalence of MSM who attend SHS from 1% to 8% over the preceding ten-year period, or the situations with reactive vaccination added to pre-emptive vaccination that increases the seroprevalence of MSM who attendees SHS by the range from 1% to 6% over the preceding ten-year period, the outbreak is periodic with a long period. Estimates listed are for the first 10 years.

Without pre-emptive vaccination, the seroprevalence or immunity level reduces from 65.8% to 62.9% at the end of a 10-year period. The incoming outbreak is assumed to be induced by importation of infections as for the 2016/18 outbreak.

**SI.4.6 Steady seroprevalence between outbreaks**

In the assessment of cost-effectiveness of vaccination in the main text, we assume that the baseline immunity level of MSM population will reduce because of inflows of the younger and more susceptible men and outflow of old and more immune men. Here we consider a situation where the baseline immunity remains unchanged over time if there was no vaccination. The results are shown in Tables S15. To achieve the same target increase in immunity, much smaller numbers of vaccines are required and therefore much money is saved. The pre-emptive vaccination is cost-effective if it increases the seroprevalence of MSM who attend SHS clinics by less than 7% over 5 years. Compared with reactive vaccination, pre-emptive vaccination is a better choice.

**Table S15** **Cost-effectiveness analysis under pre-emptive vaccination (PV) and reactive vaccination (RV) alone or in combination under the assumption of steady immunity.** No reduction in contact rate during the outbreak was assumed. Costs are in thousands of GB£ and the values of ICER are in thousands of pounds per QALY.

| Increase in seroprevalence among SHS attendees | Duration of outbreak (days) | Total cases during outbreak | Vaccination costs | Outbreak costs | Productivity losses | Total cost | QALYs | Incremental in comparing current scenario to previous scenario | | | Incremental in comparing current scenario to no vaccination | | | Incremental in comparing the combination of RV and PV to PV alone scenario | | |
| --- | --- | --- | --- | --- | --- | --- | --- | --- | --- | --- | --- | --- | --- | --- | --- | --- |
|  |  |  |  |  |  |  |  | Costs | QALYs | ICER | costs | QALYs | ICER | costs | QALYs | ICER |
| **Pre-emptive vaccination alone** **without control measures taken during the outbreak, with the outbreak cost only including costs due to Clinical Case Management** | | | | | | | | | | | | | | | | |
| 0 | 3231 | 23114 | 0 | 47670 | 2206 | 49876 | 4122941 |  |  |  |  |  |  |  |  |  |
| 0.01 | 3552^#^ | 16314 | 571 | 33292 | 1193 | 35056 | 4123241 | -14820 | 301 | CS | -14820 | 301 | CS |  |  |  |
| 0.02 | 3550^#^ | 9663 | 1332 | 19622 | 559 | 21512 | 4123528 | -13544 | 286 | CS | -28364 | 587 | CS |  |  |  |
| 0.03 | 3547^#^ | 4534 | 2283 | 9359 | 333 | 11975 | 4123742 | -9538 | 214 | CS | -37902 | 801 | CS |  |  |  |
| 0.04 | 2859 | 2153 | 3044 | 4581 | 277 | 7902 | 4123842 | -4073 | 100 | CS | -41975 | 901 | CS |  |  |  |
| 0.05 | 1830 | 1273 | 3805 | 2762 | 242 | 6809 | 4123880 | -1092 | 38 | CS | -43067 | 939 | CS |  |  |  |
| 0.06 | 1309 | 883 | 4756 | 1937 | 217 | 6910 | 4123897 | 101 | 17 | 6 | -42966 | 956 | CS |  |  |  |
| 0.07 | 1014 | 671 | 5707 | 1479 | 197 | 7384 | 4123907 | 474 | 10 | 50 | -42493 | 966 | CS |  |  |  |
| 0.08 | 826 | 538 | 6658 | 1195 | 181 | 8034 | 4123908 | 651 | 1 | 651 | -41842 | 967 | CS |  |  |  |
| 0.09 | 696 | 448 | 7610 | 997 | 167 | 8774 | 4123909 | 739 | 1 | 739 | -41103 | 968 | CS |  |  |  |
| **Reactive vaccination alone or with pre-emptive vaccination, with the outbreak cost including costs due to Clinical Case Management and Public Health Response** | | | | | | | | | | | | | | | | |
| RV alone | 1384 | 2343 | 0 | 8374 | 459 | 8833 | 4123831 |  |  |  | -41043 | 890 | CS | -41043 | 890 | CS |
| 0.01+RV | 1396 | 1947 | 571 | 6979 | 382 | 7932 | 4123849 | -901 | 18 | CS | -41944 | 908 | CS | -27124 | 608 | CS |
| 0.02+RV | 1368 | 1587 | 1332 | 5713 | 326 | 7371 | 4123865 | -561 | 16 | CS | -42505 | 925 | CS | -14141 | 338 | CS |
| 0.03+RV | 1292 | 1276 | 2283 | 4616 | 285 | 7184 | 4123879 | -188 | 14 | CS | -42693 | 939 | CS | -4791 | 137 | CS |
| 0.04+RV | 1177 | 1021 | 3044 | 3714 | 253 | 7011 | 4123891 | -173 | 11 | CS | -42866 | 950 | CS | -891 | 49 | CS |
| 0.05+RV | 1043 | 823 | 3805 | 3011 | 228 | 7044 | 4123900 | 33 | 9 | 4 | -42833 | 959 | CS | 234 | 20 | 12 |
| 0.06+RV | 911 | 673 | 4756 | 2476 | 207 | 7439 | 4123907 | 396 | 7 | 58 | -42437 | 966 | CS | 529 | 9 | 56 |
| 0.07+RV | 795 | 561 | 5707 | 2072 | 190 | 7969 | 4123912 | 530 | 5 | 104 | -41907 | 971 | CS | 585 | 5 | 118 |
| 0.08+RV | 698 | 476 | 6658 | 1764 | 176 | 8598 | 4123913 | 629 | 1 | 629 | -41279 | 972 | CS | 563 | 5 | 113 |
| 0.09+RV | 617 | 410 | 7610 | 1524 | 163 | 9297 | 4123914 | 699 | 1 | 699 | -40580 | 973 | CS | 523 | 5 | 105 |

CS denotes cost saving where the option is cheaper compared to the comparator and QALYs are gained. ^#^_:_ For the situations with pre-emptive vaccination that increases the seroprevalence of MSM who attend SHS from 1% to 3% over 5 years, the outbreak is periodic with a long period. Estimates listed are for the first 10 years.

**SI.4.7 Effect of assortative mixing among high risk MSM**

In the main text the value of the mixing parameter *b*=3.5% was used which was obtained from fitting to EMIS data. Here we consider five different values of the mixing parameter *b* to assess how the assortative mixing among high risk MSM impacts the transmission dynamics. The transmission model was refitted to the outbreak of 725 male cases under values of *b* = 10%, 20%, 30%, 40% and 50%. Other assumptions are the same as that gave rise to Table 1. The estimates of model parameters are given in Table S16. The results of Table 1 for *b*=3.5% are also included for comparison. It can be seen from Table S16 that the assortative mixing among high risk groups does not have a large impact on transmission dynamics unless the mixing parameter is large (≥40%).

**Table S16** **Effects of assortative mixing on transmission dynamics**

| Parameters | b=3.5% | b=10% | b=20% | b=30% | b=40% | b=50% |
| --- | --- | --- | --- | --- | --- | --- |
| Transmission coefficient (*β*) per week | 1.48 [1.33,1.60] | 1.49 [1.38,1.69] | 1.48 [1.31,1.63] | 1.50 [1.35,1.67] | 1.53 [1.39,1.71] | 1.57 [1.43,1.78] |
| Initial susceptibility (*ρ*)^♦^ | 0.309 [0.284,0.341] | 0.308 [0.272,0.338] | 0.308 [0.280,0.348] | 0.311 [0.279,0.346] | 0.310 [0.278,0.344] | 0.316 [0.280,0.346] |
| Mean susceptibility after outbreak | 0.296 [0.272,0.327] | 0.295 [0.260,0.324] | 0.295 [0.268,0.334] | 0.299 [0.267,0.332] | 0.298 [0.267,0.330] | 0.303 [0.268,0.332] |
| Reduction in susceptibility due to outbreak | 0.013 [0.012,0.014] | 0.013 [0.012,0.014] | 0.013 [0.012,0.014] | 0.013 [0.012,0.014] | 0.013 [0.012,0.014] | 0.013 [0.012,0.014] |
| Time when sexual contact rate decreases (*t_c_*) - weeks | 48.7 [43.1,54.1] | 48.7 [43.1,54.8] | 48.4 [42.1,53.4] | 47.2 [41.2,52.6] | 45.7 [37.8,50.1] | 42.3 [36.0,47.9] |
| Reduction in sexual contact rate (1-*w*) after t_c_ | 0.169 [0.131,0.214] | 0.163 [0.122,0.201] | 0.166 [0.128,0.208] | 0.173 [0.141,0.212] | 0.191 [0.160,0.227] | 0.213 [0.185,0.243] |
| *R*_0_ before decrease in contact rate | 3.19 [2.87,3.46] | 3.21 [2.92,3.64] | 3.20 [2.82,3.52] | 3.25 [2.91,3.61] | 3.31 [2.99,3.69] | 3.39 [3.09,3.84] |
| *R*_e_ after decrease in contact rate | 0.82 [0.77,0.85] | 0.83 [0.79,0.86] | 0.82 [0.78,0.85] | 0.83 [0.79,0.86] | 0.83 [0.80,0.87] | 0.85 [0.81,0.87] |

Further we consider how the assortative mixing of parameter *b*=20% affects the outcome of vaccination strategies. Simulation results are given in Table S17. Comparing to Table 2, Table S17 shows that, under the assortative behaviour (*b*=20%), pre-emptive vaccination can be cost-effective (<£20,000/QALY) if it increases the seroprevalence among MSM attending SHS by <10.0% over 5 years. This occurs if the yearly pre-outbreak vaccination coverage rate among SHS attendees is <10.2%. As with the weak assortative mixing (Table 2), Table S17 shows that the reactive vaccination added to a pre-emptive vaccination which increases the seroprevalence of MSM attending SHS clinics by less than 9% over 5 years is also cost-effective.

**Table S17** **Cost-effectiveness analysis under pre-emptive vaccination (PV) and reactive vaccination (RV) alone or in combination under the assortative mixing of *b*=20%.** No reduction in contact rate during the outbreak was assumed. Costs are in thousands of GB£ and the values of ICER are in thousands of pounds per QALY.

| Increase in seroprevalence among SHS attendees | Duration of outbreak (days) | Total cases during outbreak | Vaccination costs | Outbreak costs | Productivity losses | Total cos | QALYs | Incremental in comparing current scenario to previous scenario | | | Incremental in comparing current scenario to no vaccination | | | Incremental in comparing the combination of RV and PV to PV alone scenario | | |
| --- | --- | --- | --- | --- | --- | --- | --- | --- | --- | --- | --- | --- | --- | --- | --- | --- |
|  |  |  |  |  |  |  |  | Costs | QALYs | ICER | costs | QALYs | ICER | costs | QALYs | ICER |
| **Pre-emptive vaccination alone without control measures taken during the outbreak, with the outbreak cost only including costs due to Clinical Case Management** | | | | | | | | | | | | | | | | |
| 0 | 3556^#^ | 53789 | 0 | 113216 | 9463 | 122679 | 4121582 |  |  |  |  |  |  |  |  |  |
| 0.01 | 3550^#^ | 44089 | 1902 | 90450 | 5098 | 97451 | 4122058 | -25228 | 477 | CS | -25228 | 477 | CS |  |  |  |
| 0.05 | 3541^#^ | 40422 | 5517 | 75431 | 2774 | 83721 | 4122380 | -13730 | 322 | CS | -38958 | 799 | CS |  |  |  |
| 0.06 | 3538^#^ | 40699 | 6468 | 73488 | 3462 | 83419 | 4122434 | -303 | 53 | CS | -39261 | 852 | CS |  |  |  |
| 0.07 | 3536^#^ | 36765 | 7610 | 64299 | 4392 | 76300 | 4122685 | -7119 | 251 | CS | -46380 | 1103 | CS |  |  |  |
| 0.08 | 3533^#^ | 13229 | 8561 | 22996 | 3473 | 35030 | 4123549 | -41270 | 864 | CS | -87649 | 1967 | CS |  |  |  |
| 0.09 | 1296 | 607 | 9702 | 1340 | 178 | 11220 | 4123910 | -23810 | 360 | CS | -111460 | 2328 | CS |  |  |  |
| 0.10 | 870 | 473 | 10844 | 1050 | 163 | 12056 | 4123916 | 836 | 6 | 139 | -110623 | 2334 | CS |  |  |  |
| **Reactive vaccination alone or with pre-emptive vaccination, with the outbreak cost including costs due to Clinical Case Management and Public Health Response** | | | | | | | | | | | | | | | | |
| RV alone | 1511 | 4028 | 0 | 14318 | 846 | 15164 | 4123756 |  |  |  | -107515 | 2174 | CS | -107515 | 2174 | CS |
| 0.01+RV | 3550^#^ | 3104 | 1902 | 11018 | 529 | 13449 | 4123800 | -1715 | 44 | CS | -109230 | 2218 | CS | -84002 | 1741 | CS |
| 0.05+RV | 3541^#^ | 2418 | 5517 | 7957 | 424 | 13898 | 4123843 | 449 | 43 | 10 | -108781 | 2261 | CS | -69823 | 1463 | CS |
| 0.06+RV | 3538^#^ | 1939 | 6468 | 6399 | 399 | 13266 | 4123863 | -632 | 20 | CS | -109413 | 2281 | CS | -70152 | 1429 | CS |
| 0.07+RV | 1530 | 789 | 7610 | 2912 | 206 | 10728 | 4123902 | -2539 | 39 | CS | -111952 | 2320 | CS | -65572 | 1217 | CS |
| 0.08+RV | 1075 | 618 | 8561 | 2287 | 188 | 11036 | 4123909 | 308 | 8 | 40 | -111644 | 2327 | CS | -23994 | 360 | CS |
| 0.09+RV | 857 | 504 | 9702 | 1872 | 172 | 11746 | 4123914 | 711 | 5 | 138 | -110933 | 2333 | CS | 527 | 5 | 114 |
| 0.10+RV | 715 | 422 | 10844 | 1576 | 159 | 12578 | 4123918 | 832 | 4 | 226 | -110101 | 2336 | CS | 522 | 2 | 228 |

CS denotes cost saving where the option is cheaper compared to the comparator and QALYs are gained. ^#^_:_ For the situations with pre-emptive vaccination that increases the seroprevalence of MSM who attend SHS by less than 9% over 5 years, or the situations with reactive vaccination added to pre-emptive vaccination that increases the seroprevalence of MSM who attendees SHS by the range from 1% to 6% over 5 years, the outbreak is periodic with a long period. Estimates listed are for the first 10 years. Without pre-emptive vaccination, the seroprevalence or immunity level reduces from 65.8% to 64.2% at the end of a 5-year period. The incoming outbreak is assumed to be induced by importation of infections as for the 2016/18 outbreak.

**SI.4.8 The effect of variation in the protection levels among four groups of MSM**

In the main text, we assumed the four groups of MSM have the same susceptibility at the beginning of the outbreak. Here we assume that the four groups of MSM have different susceptibility with ratio set up as: 1.0:0.9:0.8:07, or 1.0:0.8:0.7:0.5. The results listed in Table S18 show that with this variation in susceptibility across the four groups of MSM, the estimate of transmissibility (R_0_) of HAV increases; at the same time the reductions in contact rate (1-*w*) due to public health response slightly decrease. This implies that a weaker composite behaviour response is required to control the 2016/18 outbreak among higher transmissible but heterogeneously susceptible MSM. Nevertheless, the goodness of the model fit judged by Deviance Information Criterion (DIC) becomes worse. This may indicate that the equal susceptibility across the four groups should be a reasonable assumption.

**Table S18** **Posterior distributions of model parameters under the heterogenous susceptibility across four groups of MSM with the ratio *h*_1_:*h*_2_:*h*_3_:*h*_4_.** The mean seroprevalence after the outbreak is 65.8% and the standard deviation is 15.0% (Table S3). The situation where the four groups are equally susceptible is included for comparison.

| **Parameters** | **Ratios of initial susceptibility across four groups of MSM** | | |
| --- | --- | --- | --- |
|  | **1.0:1.0:1.0:1.0 (average 1.0)** | **1.0:0.9:0.8:0.7 (average 0.911)** | **1.0:0.8:0.7:0.5 (average 0.843)** |
| Transmission coefficient (*β*) per week | 1.48 [1.33,1.60] | 1.62[1.48,1.78] | 1.72[1.57,1.88] |
| Initial susceptibility (*ρ*)^♦^ | 0.309 [0.284,0.341] | 0.306[0.276,0.343] | 0.315[0.288,0.344] |
| Mean susceptibility after outbreak | 0.296 [0.272,0.327] | 0.268[0.241,0.300] | 0.256[0.234,0.280] |
| Reduction in susceptibility due to outbreak | 0.013 [0.012,0.014] | 0.042[0.038,0.047] | 0.070[0.064,0.076] |
| Time when sexual contact rate decreases (*t_c_*) - weeks | 48.7 [43.1,54.1] | 50.8[45.0,57.3] | 52.6[45.8,60.0] |
| Reduction in sexual contact rate (1-*w*) after t_c_ | 0.169 [0.131,0.214] | 0.143[0.102,0.194] | 0.126[0.080,0.178] |
| *R*_0_ before decrease in contact rate | 3.19 [2.87,3.46] | 3.50[3.11,3.80] | 3.72[3.40,4.07] |
| *R*_e_ after decrease in contact rate | 0.82 [0.77,0.85] | 1.01[0.95,1.05] | 1.22[1.14,1.27] |
| Gelman et al (2004)’s DIC | 301.53 | 311.53 | 318.81 |

^♦^: the initial susceptibility over the four groups are: *ρ*_1_= *h*_1_*ρ, ρ*_2_*=* *h*_2_*ρ, ρ*_3_*=* *h*_3_*ρ,* and *ρ*_4_= *h*_4_*ρ.* The mean susceptibility is calculated as *ρ,*(*N*_1_*h*_1_+ *N*_2_*h*_2_+ *N*_3_*h*_3_+*N*_4_*h*_4_)/*N*.

Further we consider how the variation in the susceptibility levels with ratio 1:0.9:0.8:0.7 affects the outcome of vaccination strategies. Simulation results are given in Table S19. Comparing to Table 2, Table S19 shows that, under the heterogeneous immunity level among MSM groups, pre-emptive vaccination can be cost-effective (<£20,000/QALY) if it increases the seroprevalence among MSM attending SHS by <9.0% over 5 years. This occurs if the yearly pre-outbreak vaccination coverage rate among SHS attendees is <9.1%. As with the homogeneous levels of immunity (Table 2), Table S19 shows that the reactive vaccination added to a pre-emptive vaccination which increases the seroprevalence of MSM attend SHS clinics by less than 7% over 5 years is also cost-effective.

**Table S19** **Cost-effectiveness analysis under pre-emptive vaccination (PV) and reactive vaccination (RV) alone or in combination when four groups of MSM have different susceptibility with ratio of 1.0:0.9:0.8:07.** No reduction in contact rate during the outbreak was assumed. Costs are in thousands of GB£ and the values of ICER are in thousands of pounds per QALY.

| Increase in seroprevalence among SHS attendees | Duration of outbreak (days) | Total cases during outbreak | Vaccination costs | Outbreak costs | Productivity losses | Total cos | QALYs | Incremental in comparing current scenario to previous scenario | | | Incremental in comparing current scenario to no vaccination | | | Incremental in comparing the combination of RV and PV to PV alone scenario | | |
| --- | --- | --- | --- | --- | --- | --- | --- | --- | --- | --- | --- | --- | --- | --- | --- | --- |
|  |  |  |  |  |  |  |  | Costs | QALYs | ICER | costs | QALYs | ICER | costs | QALYs | ICER |
| **Pre-emptive vaccination alone** without control measures taken during the outbreak, with the outbreak cost only including costs due to Clinical Case Management | | | | | | | | | | | | | | | | |
| 0 | 3560^#^ | 49737 | 0 | 102886 | 6527 | 109412 | 4121800 |  |  |  |  |  |  |  |  |  |
| 0.01 | 3555^#^ | 45577 | 2663 | 90659 | 3715 | 97038 | 4122061 | -12374 | 261 | CS | -12374 | 261 | CS |  |  |  |
| 0.05 | 3545^#^ | 45881 | 6658 | 81174 | 4939 | 92771 | 4122296 | -4267 | 236 | CS | -16641 | 496 | CS |  |  |  |
| 0.06 | 3543^#^ | 31326 | 7800 | 54059 | 6156 | 68015 | 4122974 | -24756 | 677 | CS | -41397 | 1173 | CS |  |  |  |
| 0.07 | 3541^#^ | 3583 | 8941 | 6460 | 1135 | 16537 | 4123829 | -51478 | 855 | CS | -92876 | 2029 | CS |  |  |  |
| 0.08 | 898 | 545 | 10083 | 1209 | 189 | 11480 | 4123913 | -5056 | 84 | CS | -97932 | 2112 | CS |  |  |  |
| 0.09 | 707 | 450 | 11414 | 1004 | 174 | 12593 | 4123917 | 1113 | 4 | 257 | -96819 | 2117 | CS |  |  |  |
| 0.10 | 593 | 384 | 12746 | 857 | 162 | 13766 | 4123920 | 1173 | 3 | 389 | -95647 | 2120 | CS |  |  |  |
| **Reactive vaccination alone or with pre-emptive vaccination**, with the outbreak cost including costs due to Clinical Case Management and Public Health Response | | | | | | | | | | | | | | | | |
| RV alone | 3560^#^ | 3557 | 0 | 12602 | 654 | 13256 | 4123780 |  |  |  | -96156 | 1980 | CS | -96156 | 1980 | CS |
| 0.01+RV | 3555^#^ | 3392 | 2663 | 11427 | 568 | 14658 | 4123799 | 1402 | 19 | 75 | -94754 | 1999 | CS | -82380 | 1738 | CS |
| 0.05+RV | 3545^#^ | 1895 | 6658 | 6194 | 465 | 13317 | 4123867 | -1341 | 68 | CS | -96095 | 2067 | CS | -79454 | 1571 | CS |
| 0.06+RV | 1106 | 708 | 7800 | 2610 | 217 | 10627 | 4123905 | -2690 | 38 | CS | -98785 | 2105 | CS | -57388 | 932 | CS |
| 0.07+RV | 872 | 577 | 8941 | 2131 | 199 | 11271 | 4123911 | 644 | 6 | 109 | -98141 | 2111 | CS | -5265 | 82 | CS |
| 0.08+RV | 724 | 484 | 10083 | 1794 | 184 | 12061 | 4123915 | 789 | 4 | 187 | -97351 | 2115 | CS | 581 | 3 | 208 |
| 0.09+RV | 621 | 415 | 11414 | 1542 | 171 | 13127 | 4123919 | 1067 | 3 | 340 | -96285 | 2118 | CS | 535 | 2 | 332 |
| 0.10+RV | 543 | 362 | 12746 | 1351 | 159 | 14256 | 4123921 | 1128 | 2 | 466 | -95156 | 2121 | CS | 490 | 1 | 485 |

^#^_:_ For the situations with pre-emptive vaccination that increases the seroprevalence of MSM who attend SHS by less than 8% over 5 years, or the situations with reactive vaccination added to pre-emptive vaccination that increases the seroprevalence of MSM who attendees SHS by less than 7% over 5 years, the outbreak is periodic with a long period. Estimates listed are for the first 10 years. Without pre-emptive vaccination, the seroprevalence or immunity level reduces from 65.8% to 64.7% at the end of a 5-year period. The incoming outbreak is assumed to be induced by importation of infections as for the 2016/18 outbreak.

**SI.4.9 Effect of transmission of mildly symptomatic cases**

In the main text we assume the symptomatic cases were not sexually active and therefore did not contribute to the spread of HAV. In reality, cases with mild symptom might still have sexual contacts with others MSM and hence contributed to the transmission of HAV. In view of this, in this appendix we include the transmission due to the mildly symptomatic cases and assess how this alters the transmission dynamics. For simplicity, we assume those who did not admit to hospitals or visit GP during the outbreak had mild symptoms; there were 158 such cases, occupying ξ= 20.5% of the 796 cases. Hence, we propose that mildly symptomatic cases can transmit HAV by continuously contacting others during their infectious periods. Further dividing the symptomatic cases into mildly (*M*) and severe (*D*) symptomatic cases and assuming *M* cases can transmit HAV during its infectious period *d*_2_, equation (S1) becomes

$\frac{dS_{j}(t)}{dt}=-\lambda_{j}S_{j}\left( t \right)-\Omega_{j}\left( t \right)\delta\left( t-t^{import} \right)-\left( t-t^{vacc} \right)(\frac{S_{j}\left( t \right)}{N_{j}^{*}\left( t \right)})[\frac{V_{j}^{GUM}\left( t \right)}{1+q}+V_{j}^{PC}$(t)],

$\frac{dE_{j}\left( t \right)}{dt}=\lambda_{j}S_{j}\left( t \right)-\frac{E_{j}\left( t \right)}{L}$,

$\frac{dO_{j}\left( t \right)}{dt}=\frac{E_{j}\left( t \right)}{L}+\Omega_{j}\delta\left( t-t^{import} \right)-\frac{O_{j}\left( t \right)}{d_{1}}$,

$\frac{dM_{j}(t)}{dt}=\frac{O_{j(t)}}{d_{1}}-\frac{M_{j}(t)}{d_{2}}$,

$\frac{dD_{j}(t)}{dt}={(p}_{S}-)\frac{O_{j(t)}}{d_{1}}-\frac{D_{j}(t)}{d_{D}}$, (S1a)

$\frac{dA_{j}(t)}{dt}={(1-p}_{S})\frac{O_{j}(t)}{d_{1}}-\frac{A_{j}\left( t \right)}{d_{2}}$.

$\frac{dR_{j}(t)}{dt}=\frac{D_{j}(t)}{d_{D}}+\frac{A_{j}(t)}{d_{2}}+\frac{M_{j}(t)}{d_{2}}+\left( t-t^{vacc} \right)(\frac{S_{j}\left( t \right)}{N_{j}^{*}\left( t \right)})[\frac{V_{j}^{GUM}\left( t \right)}{1+q}+V_{j}^{PC}(t$).

Here $N_{j}^{*}\left( t \right)=N_{j}{-D}_{j}(t){-M}_{j}(t)$ is the number of MSM in group *j* without illness due to HAV. The force of infection of the model system (S1) becomes

$\lambda_{j}\left( t \right)=\left\{ \begin{matrix} \begin{matrix} \beta\sum_{i=1}^{4} \Pi_{j,i}\left( O_{i}\left( t \right)+A_{i}\left( t \right)+M_{i}(t) \right) & t<t_{c} \end{matrix} \\ \begin{matrix} w\beta\sum_{i=1}^{4} \Pi_{j,i}(O_{i}\left( t \right)+A_{i}(t)+M_{i}(t)) & t\geq t_{c} \end{matrix} \end{matrix} \right.$ (S2a)

and the expression of basic reproduction number is

$R_{0}=\left[ d_{1}+\left( 1-p_{S} \right)d_{2}+d_{2} \right]$ (S5a)

We refit the transmission model to the outbreak of 725 male cases under this assumption with estimates of model parameters are given in Table S8. Other assumptions are the same as that gave rise to Table 1. The results for the case where symptomatic cases do not contribute to the spread of HAV are also included for comparison. It can be seen from Table S8 that although the transmission coefficient decreases from 1.48 to 1.34 per week, the basic reproduction number remains nearly the same.

Further we consider how the transmission of such mildly symptomatic infections affects the outcome of vaccination strategies. As defined above, these infections have mild symptoms and so do not get vaccinated. To control the spread of HAV, a stronger pre-emptive vaccination is required (Figures S5 and S6): periodic epidemics are only avoided when PV rate is larger 25% over the 5 year pre-outbreak period, which is more than twice the rate of pre-emptive vaccination for the situation where all symptomatic cases are not assumed to transmit HAV (see Figures 3 and S3). With reactive vaccination added, it still requires a PV rate larger than 24% over the 5 year pre-outbreak period to avoid periodic epidemics (right panels of Figure S5).

Simulation results for the cost-effectiveness of vaccinations are given in Table S20. Comparing to Table 2, Table S20 shows that, when the mildly symptomatic cases also transmit HAV, pre-emptive vaccination can be cost-effective (<£20,000/QALY) if it increases the seroprevalence among MSM attending SHS by ≤25.5% over 5 years. This occurs if the yearly pre-outbreak vaccination coverage rate among SHS attendees is <43%. Table S20 also shows that reactive vaccination is also cost-effective, but less so than pre-emptive vaccination.

**Just Pre-emptive vaccination Pre-emptive vaccination plus reactive vaccination**


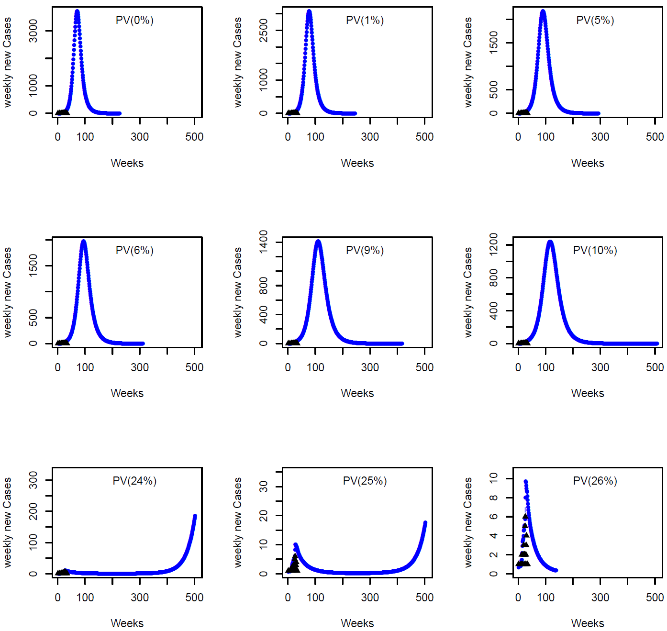

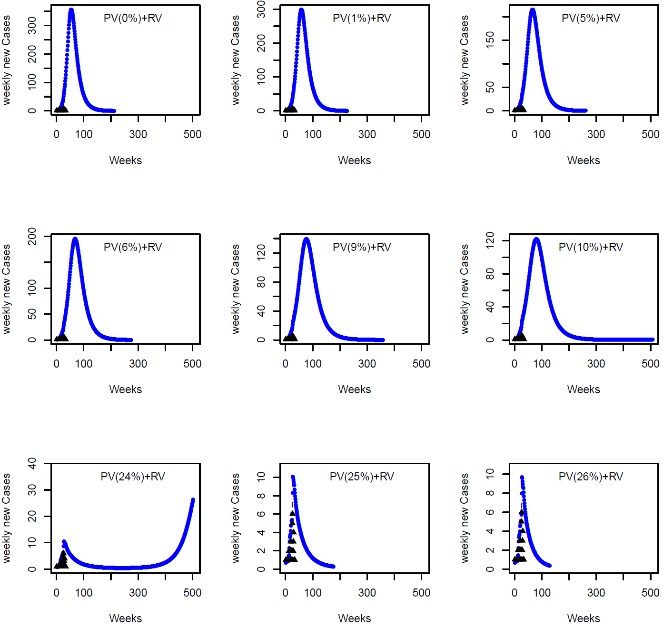


**Figure S5** **Effect of different levels of pre-emptive vaccination without (left figures) and with reactive (right figures) vaccination on the modelled 2023 outbreak of HAV among MSM population** **when mildly symptomatic cases can transmit HAV**. The percentage in PV(%) is the increase in seroprevalence of MSM who attend SHS clinics due to pre-emptive vaccination over the 5 year pre-outbreak period. The outbreak is started by imported cases (black dots) as for the 2016-18 outbreak in England.

**Just Pre-emptive vaccination Pre-emptive vaccination plus reactive vaccination**


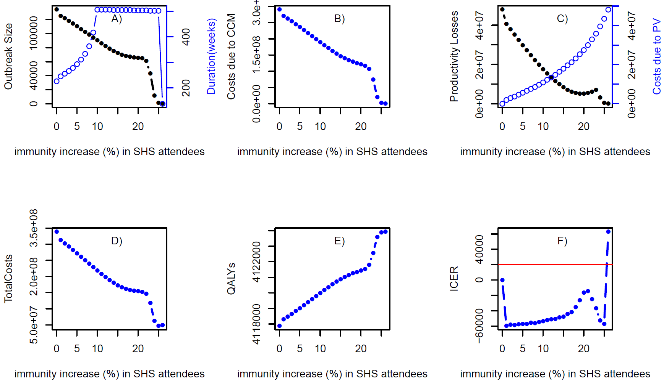

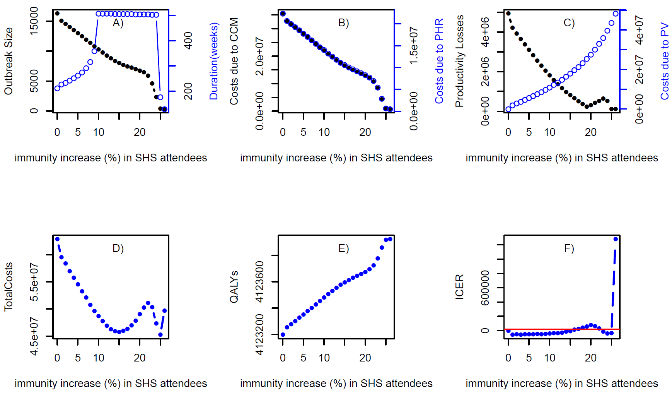


**Figure S6** **Effect of different increases in seroprevalence/immunity among SHS attenders due to pre-emptive vaccination without (left figures) and with reactive (right figures) vaccination on the modelled costs, QALYs and ICER for the 2023 HAV outbreak when mildly symptomatic cases can transmit HAV**: A) outbreak size and duration; B) Costs due to Clinical case managements and Public Health Responses (if reactive vaccination too); C) Productivity Losses and Costs due to pre-emptive vaccination; D) Total costs, E) QALYs gained within 10 years, and F) ICER. Note that if the outbreak duration is about 10 years, it implies that the outbreak is oscillating.

**Table S20** **Cost-effectiveness analysis under pre-emptive vaccination (PV) and reactive vaccination (RV) alone or in combination when the mildly symptomatic cases can transmit HAV.** No reduction in contact rate during the outbreak was assumed. Costs are in thousands of GB£ and the values of ICER are in thousands of pounds per QALY.

| Increase in seroprevalence among SHS attendees | Duration of outbreak (days) | Total cases during outbreak | Vaccination costs | Outbreak costs | Productivity losses | Total cost | QALYs | Incremental in comparing current scenario to previous scenario | | | Incremental in comparing current scenario to no vaccination | | | Incremental in comparing the combination of RV and PV to PV alone scenario | | |
| --- | --- | --- | --- | --- | --- | --- | --- | --- | --- | --- | --- | --- | --- | --- | --- | --- |
|  |  |  |  |  |  |  |  | Costs | QALYs | ICER | costs | QALYs | ICER | costs | QALYs | ICER |
| **Pre-emptive vaccination alone without control measures taken during the outbreak, with the outbreak cost only including costs due to Clinical Case Management** | | | | | | | | | | | | | | | | |
| 0 | 1582 | 134403 | 0 | 290390 | 48047 | 338438 | 4117885 |  |  |  |  |  |  |  |  |  |
| 0.01 | 1721 | 125377 | 1902 | 270226 | 40716 | 312844 | 4118313 | -25593 | 428 | CS | -25593 | 428 | CS |  |  |  |
| 0.05 | 2055 | 110381 | 5517 | 236312 | 29811 | 271640 | 4119025 | -10503 | 183 | CS | -66798 | 1140 | CS |  |  |  |
| 0.06 | 2179 | 106453 | 6468 | 227295 | 27218 | 260982 | 4119212 | -10658 | 187 | CS | -77456 | 1327 | CS |  |  |  |
| 0.09 | 2920 | 94344 | 9702 | 199342 | 19891 | 228936 | 4119789 | -32046 | 578 | CS | -109502 | 1905 | CS |  |  |  |
| 0.10 | 3550^#^ | 90293 | 10844 | 189957 | 17635 | 218436 | 4119985 | -10499 | 195 | CS | -120002 | 2100 | CS |  |  |  |
| 0.24 | 3517^#^ | 11743 | 39570 | 20334 | 3258 | 63161 | 4123601 | -155275 | 3616 | CS | -275276 | 5716 | CS |  |  |  |
| 0.25 | 3515^#^ | 1583 | 43565 | 2937 | 435 | 46937 | 4123885 | -16225 | 285 | CS | -291501 | 6000 | CS |  |  |  |
| 0.255 | 1152 | 389 | 45848 | 868 | 118 | 46834 | 4123919 | -103 | 34 | CS | -291604 | 6034 | CS |  |  |  |
| 0.26 | 971 | 349 | 48321 | 778 | 114 | 49213 | 4123921 | 2379 | 2 | 1310 | -289224 | 6036 | CS |  |  |  |
| **Reactive vaccination alone or with pre-emptive vaccination, with the outbreak cost including costs due to Clinical Case Management and Public Health Response** | | | | | | | | | | | | | | | | |
| RV alone | 1484 | 16308 | 0 | 57886 | 4943 | 62830 | 4123198 |  |  |  | -275608 | 5313 | CS | -275608 | 5313 | CS |
| 0.01+RV | 1585 | 15078 | 1902 | 53383 | 4213 | 59498 | 4123255 | -3332 | 57 | CS | -278940 | 5371 | CS | -253346 | 4943 | CS |
| 0.05+RV | 1831 | 13020 | 5517 | 45893 | 3100 | 54510 | 4123351 | -4988 | 96 | CS | -283927 | 5467 | CS | -217130 | 4327 | CS |
| 0.06+RV | 1923 | 12477 | 6468 | 43924 | 2831 | 53222 | 4123377 | -1288 | 25 | CS | -285215 | 5492 | CS | -207759 | 4166 | CS |
| 0.09+RV | 2506 | 10803 | 9702 | 37858 | 2054 | 49615 | 4123455 | -3607 | 78 | CS | -288823 | 5570 | CS | -179321 | 3666 | CS |
| 0.10+RV | 3550^#^ | 10293 | 10844 | 36000 | 1815 | 48658 | 4123479 | -956 | 25 | CS | -289779 | 5595 | CS | -169778 | 3495 | CS |
| 0.24+RV | 3517^#^ | 2366 | 39570 | 7225 | 515 | 47310 | 4123858 | -1348 | 378 | CS | -291127 | 5973 | CS | -15851 | 258 | CS |
| 0.25+RV | 1232 | 414 | 43565 | 1569 | 121 | 45254 | 4123918 | -2056 | 60 | CS | -293183 | 6034 | CS | -1682 | 33 | CS |
| 0.255+RV | 1032 | 371 | 45848 | 1406 | 117 | 47371 | 4123920 | 2116 | 2 | 1083 | -291067 | 6036 | CS | 536 | 0.84 | 641 |
| 0.26+RV | 907 | 336 | 48321 | 1277 | 113 | 49711 | 4123922 | 2340 | 2 | 1528 | -288727 | 6037 | CS | 498 | 0.55 | 900 |

^#^_:_ For the situations with pre-emptive vaccination that increases the seroprevalence of MSM who attend SHS in the range from 10% to 25% over 5 years, or the situations with reactive vaccination added to pre-emptive vaccination that increases the seroprevalence of MSM who attendees SHS in the range from 10% to 24% over 5 years, the outbreak is periodic with a long period. Estimates listed are for the first 10 years.

Without pre-emptive vaccination, the seroprevalence or immunity level reduces from 65.8% to 64.2% at the end of a 5-year period. The incoming outbreak is assumed to be induced by importation of infections as for the 2016/18 outbreak.

**SI.4.10 Effects of duration of immunity induced by one dose of vaccine and the return rate for 2^nd^ dose of vaccination.**

In the main text we use the duration of 7 years for immunity induced by one dose vaccine. Though we assume the return rate for 2^nd^ dose of vaccine is *q*=50% in the main text, the potential rates might vary around this value. Here we consider how a short immunity duration (5 years) and different return rates for 2^nd^ dose vaccine could affect our results. Results are given in Tables S21 and S22.

Given the return rate for 2^nd^ dose vaccine, a short duration of protection will require a larger number of vaccines and cost more to achieve a certain increase in immunity of SHS attendees, but the size of the change is small (Tables S21 and S22). For example, given *q*=50%, reducing immunity duration from 7 to 5 years will increase by 5.9% the number and cost of vaccines required to increase the immunity level of SHS attendees by 9%. With a high return rate for the 2^nd^ dose vaccine, the total number of vaccines required to achieve an increase in SHS attendees increases, but this only causes a tiny change in the cost of pre-emptive vaccination (Tables S21 and S22). For example, assuming the duration of immunity induced by the one vaccine dose is 7 years, increasing the return rate for 2^nd^ dose vaccines from 25% to 75% increases by 18% the number of vaccines required to increase the immunity of SHS attendees by 9% while the cost of pre-emptive vaccination decreases by 0.3%.

For our modelled outbreak in 2023 with no reactive vaccination and no reduction in contact rate during the outbreak, our results in Table S22 show that maximum annual coverage of pre-emptive vaccination that is cost-effective is not effected much by changes in the duration of immunity from one vaccine dose or changes in the return rate for 2^nd^ dose vaccine.

**Table S21** **Effect of pre-emptive vaccination in increasing the immunity level/seroprevalence among MSM who attend SHS clinics and all MSM for different assumptions of the return rate for the 2^nd^ vaccine dose rate and duration of immunity from 1 vaccine dose.** Also shows effect on yearly coverage, number of vaccines required, and the total cost involved. Here we consider an increase in seroprevalence among SHS attendees of 5% during the non-outbreak period with an overall increase in seroprevalence of 1.45%.

| **Protection duration of 1^st^ dose and return rate for 2 dose (q)** | **Required Yearly coverage of vaccination** | **Number vaccines needed** | **Cost (1000s pounds)** |
| --- | --- | --- | --- |
| *q*=50%; 7-year protection | 5.18% | 94,708 | 5,516 |
| *q*=50%; 5-year protection | 5.54% | 101,240 | 5,897 |
| *q*=25%; 7-year protection | 5.71% | 87,088 | 5,565 |
| *q*=75%; 7-year protection | 4.82% | 102,873 | 5,577 |
| *q*=25%; 5-year protection | 6.25% | 95,253 | 6,086 |
| *q*=75%; 5-year protection | 4.82% | 102,873 | 5,577 |

Note: At the start, the immunity level of MSM is set to 65.8%, the seroprevalence post the 2016/18 outbreak. As pre-emptive vaccination is given via SHS clinics, non-SHS attendees will not be vaccinated during the 5-year non-outbreak period. The immunity level among non-SHS attendees decreases by 1.57%.

**Table S22** **Cost-effectiveness analysis of pre-emptive vaccination with no control measures taken during the outbreak, including no reactive vaccination or reduction in contact rate.** The total outbreak cost comes from clinical cases management (CCM). Costs are in thousands of GB£ and ICER are in thousands of pounds per QALY. Compared to baseline, the duration of immunity after 1 dose of vaccine is set to X years and the return rate for 2^nd^ dose is *q*.

|  | **Pre-emptive vaccination** | | | | **Outbreak** | | | | | |  |
| --- | --- | --- | --- | --- | --- | --- | --- | --- | --- | --- | --- |
| **Target increase in immunity of SHS attendees** | **Overall change in seroprevalence over the whole MSM population** | **Yearly coverage** | **Vaccines required** | **Cost** | **Duration (days)** | **Total cases** | **Vaccine Costs** | **PL** | **Total costs (£)** | **QALYs** | **ICER compared to previous scenario** |
|  | X=7, q=50% (baseline situation) | | | |  |  |  |  |  |  |  |
| 0% | -1.57% | 0 | 0 | 0 | 3,559 | 52,951 | 117,892 | 8,989 | 126,881 | 5,312,148 | – |
| 8.0% | 2.83% | 8.04% | 146,961 | 8,560 | 3,538 | 3,820 | 8,441 | 1,377 | 18,378 | 5,315,387 | CS |
| 8.5% | 3.06% | 8.57% | 156,759 | 9,131 | 1,356 | 656 | 1,436 | 189 | 10,756 | 5,315,547 | CS |
| 9.0% | 3.29% | 9.11% | 166,556 | 9,702 | 1,056 | 574 | 1,264 | 184 | 11,150 | 5,315,553 | 76 |
| 10.0% | 3.75% | 10.18% | 186,151 | 10,843 | 802 | 468 | 1,046 | 171 | 12,060 | 5,315,560 | 130 |
|  | X=5, q=50%. | | | |  |  |  |  |  |  |  |
| 0% | -1.57% | 0 | 0 | 0 | 3,559 | 52,951 | 117,892 | 8,989 | 126,881 | 5,312,148 | – |
| 8.0% | 2.83% | 8.57% | 156,759 | 9,131 | 3,538 | 3,820 | 8,441 | 1,377 | 18,949 | 5,315,387 | CS |
| 8.5% | 3.06% | 9.11% | 166,556 | 9,702 | 1,356 | 656 | 1,436 | 189 | 11,327 | 5,315,547 | CS |
| 9.0% | 3.29% | 9.64% | 176,354 | 10,272 | 1,056 | 574 | 1,264 | 184 | 11,720 | 5,315,553 | 76.2 |
| 10.0% | 3.75% | 10.89% | 199,214 | 11,604 | 802 | 468 | 1,046 | 171 | 12,821 | 5,315,560 | 157.3 |
|  | X=7, q=25%. | | | |  |  |  |  |  |  |  |
| 0% | -1.57% | 0 | 0 | 0 | 3,559 | 52,951 | 117,892 | 8,989 | 126,881 | 5,312,148 | – |
| 8.0% | 2.83% | 8.93% | 136,075 | 8,695 | 3,538 | 3,820 | 8,441 | 1,377 | 18,513 | 5,315,387 | CS |
| 8.5% | 3.06% | 9.46% | 144,240 | 9,217 | 1,356 | 656 | 1,436 | 189 | 10,842 | 5,315,547 | CS |
| 9.0% | 3.29% | 10.0% | 152,404 | 9,738 | 1,056 | 574 | 1,264 | 184 | 11,186 | 5,315,553 | 66.7 |
| 10.0% | 3.75% | 11.25% | 171,455 | 10,956 | 802 | 468 | 1,046 | 171 | 12,173 | 5,315,560 | 141.0 |
|  | X=7, q=75%. | | | |  |  |  |  |  |  |  |
| 0% | -1.57% | 0 | 0 | 0 | 3,559 | 52,951 | 117,892 | 8,989 | 126,881 | 5,312,148 | – |
| 8.0% | 2.83% | 7.32% | 156,215 | 8,469 | 3,538 | 3,820 | 8,441 | 1,377 | 18,287 | 5,315,387 | CS |
| 8.5% | 3.06% | 7.86% | 167,645 | 9,088 | 1,356 | 656 | 1,436 | 189 | 10,713 | 5,315,547 | CS |
| 9.0% | 3.29% | 8.39% | 179,075 | 9,708 | 1,056 | 574 | 1,264 | 184 | 11,156 | 5,315,553 | 85.9 |
| 10.0% | 3.75% | 9.29% | 198,126 | 10,741 | 802 | 468 | 1,046 | 171 | 11,958 | 5,315,560 | 114.6 |
|  | X=5, q=25%. | | | |  |  |  |  |  |  |  |
| 0% | -1.57% | 0 | 0 | 0 | 3,559 | 52,951 | 117,892 | 8,989 | 126,881 | 5,312,148 | – |
| 8.0% | 2.83% | 9.64% | 146,961 | 9,391 | 3,538 | 3,820 | 8,441 | 1,377 | 19,209 | 5,315,387 | CS |
| 8.5% | 3.06% | 10.36% | 157,847 | 10,086 | 1,356 | 656 | 1,436 | 189 | 11,711 | 5,315,547 | CS |
| 9.0% | 3.29% | 11.07% | 168,733 | 10,782 | 1,056 | 574 | 1,264 | 184 | 12,230 | 5,315,553 | 100.6 |
| 10.0% | 3.75% | 12.32% | 187,784 | 11,999 | 802 | 468 | 1,046 | 171 | 13,216 | 5,315,560 | 140.9 |
|  | X=5, q=75% | | | |  |  |  |  |  |  |  |
| 0% | -1.57% | 0 | 0 | 0 | 3,559 | 52,951 | 117,892 | 8,989 | 126,881 | 5,312,148 | – |
| 8.0% | 2.83% | 7.50% | 160,025 | 8,675 | 3,538 | 3,820 | 8,441 | 1,377 | 18,493 | 5,315,387 | CS |
| 8.5% | 3.06% | 8.04% | 171,455 | 9,295 | 1,356 | 656 | 1,436 | 189 | 10,920 | 5,315,547 | CS |
| 9.0% | 3.29% | 8.57% | 182,885 | 9,915 | 1,056 | 574 | 1,264 | 184 | 11,363 | 5,315,553 | 85.9 |
| 10.0% | 3.75% | 9.64% | 205,746 | 11,154 | 802 | 468 | 1,046 | 171 | 12,371 | 5,315,560 | 144.0 |

^#^_:_ For the situation without pre-emptive vaccination, the outbreak is periodic with a long period. Here listed is the value within the first 10 years.

Without pre-emptive vaccination, the protection level reduces from 65.8% to 64.2% at the end of a 5-year period. The incoming outbreak is assumed to be induced by importation as that during the 2016/18 outbreak.

**SI.4.11 Enhanced reactive vaccination**

In the main text the rate of reactive vaccination is defined by two ratios: k^PC^ =3.31 and k^SHS^ =34.41 compared to the number of outbreak cases, which were obtained from the 2016-18 outbreak. In this sensitivity analysis, we double the rate of reactive vaccination by doubling these two ratios. The results are shown in Table S23. With the enhanced RV, periodic epidemics are avoided, and the RV becomes a slightly better choice in view of the total costs of outbreaks (11149 k£ versus 10194 k£).

**Table S23** **Cost-effectiveness analysis under pre-emptive vaccination (PV) and reactive vaccination (RV) alone or in combination when the strength of RV doubled.** No reduction in contact rate during the outbreak was assumed. Costs are in thousands of GB£ and ICER are in thousands of pounds per QALY.

| Increase in seroprevalence among SHS attendees | Duration of outbreak (days) | Total cases during outbreak | Vaccination costs | Outbreak costs | Productivity losses | Total cos | QALYs | Incremental in comparing current scenario to previous scenario | | | Incremental in comparing current scenario to baseline scenario | | | Incremental - comparing the combination of RV and PV to PV alone | | |
| --- | --- | --- | --- | --- | --- | --- | --- | --- | --- | --- | --- | --- | --- | --- | --- | --- |
|  |  |  |  |  |  |  |  | costs | QALYs | ICER | costs | QALYs | ICER | costs | QALYs | ICER |
| **Pre-emptive vaccination alone without control measures taken during the outbreak, with the outbreak cost only including costs due to Clinical Case Management** | | | | | | | | | | | | | | | | |
| 0(baseline) | 3558^#^ | 52961 | 0 | 110978 | 8531 | 119509 | 4121628 |  |  |  |  |  |  |  |  |  |
| 0.01 | 3553^#^ | 44109 | 1902 | 89625 | 4478 | 96006 | 4122076 | -23503 | 448 | CS | -23503 | 448 | CS |  |  |  |
| 0.02 | 3551^#^ | 42404 | 2853 | 84460 | 3409 | 90723 | 4122186 | -5282 | 110 | CS | -28785 | 558 | CS |  |  |  |
| 0.03 | 3549^#^ | 41579 | 3614 | 80848 | 2745 | 87207 | 4122264 | -3515 | 77.06 | CS | -32300 | 635 | CS |  |  |  |
| 0.04 | 3547^#^ | 41466 | 4565 | 78423 | 2662 | 85651 | 4122316 | -1555 | 52.8 | CS | -33856 | 688 | CS |  |  |  |
| 0.05 | 3544^#^ | 41785 | 5516 | 76581 | 3147 | 85246 | 4122362 | -405 | 45.5 | CS | -34262 | 733 | CS |  |  |  |
| 0.06 | 3542^#^ | 40673 | 6468 | 72112 | 3996 | 82576 | 4122488 | -2669 | 125 | CS | -36931 | 859 | CS |  |  |  |
| 0.07 | 3540^#^ | 26463 | 7609 | 45879 | 4947 | 58437 | 4123111 | -24139 | 623 | CS | -61071 | 1482 | CS |  |  |  |
| 0.08 | 3537^#^ | 3825 | 8560 | 6904 | 1081 | 16546 | 4123818 | -41890 | 707 | CS | -102961 | 2189 | CS |  |  |  |
| 0.09 | 1054 | 571 | 9702 | 1267 | 180 | 11149 | 4123911 | -5397 | 93 | CS | -108359 | 2282 | CS |  |  |  |
| 0.1 | 797 | 464 | 10843 | 1031 | 166 | 12041 | 4123916 | 891 | 4.8 | 184 | -107467 | 2287 | CS |  |  |  |
| Enhanced reactive vaccination alone or with pre-emptive vaccination, with the outbreak cost including costs of Clinical Case Management and Public Health Response | | | | | | | | | | | | | | | | |
| RV alone | 1159 | 2063 | 0 | 9678 | 516 | 10194 | 4123843 |  |  |  | -109314 | 2215 | CS | -109313 | 2215.0 | CS |
| 0.01+RV | 1267 | 1537 | 1902 | 7230 | 365 | 9498 | 4123867 | -696 | 24.0 | CS | -110010 | 2239 | CS | -86507 | 1790.9 | CS |
| 0.02+RV | 1285 | 1341 | 2853 | 6321 | 323 | 9498 | 4123876 | 0.6 | 8.9 | 0.1 | -110010 | 2247 | CS | -81224 | 1689.7 | CS |
| 0.03+RV | 1268 | 1155 | 3614 | 5465 | 288 | 9367 | 4123885 | -130 | 8.4 | CS | -110140 | 2256 | CS | -77839 | 1621.0 | CS |
| 0.04+RV | 1207 | 984 | 4565 | 4668 | 259 | 9493 | 4123892 | 125 | 7.7 | 16.3 | -110015 | 2264 | CS | -76158 | 1575.9 | CS |
| 0.05+RV | 1106 | 832 | 5516 | 3965 | 235 | 9717 | 4123899 | 224 | 6.8 | 32.8 | -109790 | 2270 | CS | -75528 | 1537.2 | CS |
| 0.06+RV | 987 | 704 | 6468 | 3359 | 215 | 10043 | 4123905 | 325 | 5.8 | 56.0 | -109465 | 2276 | CS | -72533 | 1417.3 | CS |
| 0.07+RV | 871 | 598 | 7609 | 2864 | 198 | 10672 | 4123910 | 629 | 4.8 | 131.6 | -108836 | 2281 | CS | -47764 | 798.7 | CS |
| 0.08+RV | 766 | 513 | 8560 | 2462 | 183 | 11207 | 4123914 | 534 | 3.9 | 138.6 | -108301 | 2285 | CS | -5339 | 95.6 | CS |
| 0.09+RV | 677 | 444 | 9702 | 2140 | 170 | 12013 | 4123917 | 806 | 3.1 | 259.8 | -107495 | 2288 | CS | 863 | 5.8 | 150.0 |
| 0.1+RV | 603 | 389 | 10843 | 1884 | 159 | 12887 | 4123919 | 874 | 2.5 | 349.5 | -106621 | 2290 | CS | 846 | 3.4 | 247.7 |

^#^_:_ For the situations with pre-emptive vaccination that increases the seroprevalence of MSM who attend SHS by less than 9% over the 5 year pre-outbreak period, the outbreak is periodic with a long period. Estimates listed are for the first 10 years. Without pre-emptive vaccination, the seroprevalence or immunity level reduces from 65.8% to 64.2% at the end of a 5-year period. The incoming outbreak is assumed to be induced by importation of infections as for the 2016/18 outbreak.

**SI.4.12 Effect of reduction in contact rate due to sexual behavioural change during the outbreak**

In the main text we assumed that there was no reduction in contact rate between MSM in the 2023 outbreak. In this sensitivity analysis, we included the 16.9% reduction in contact rate from week 49 since the onset of the modelled 2023 outbreak as in the 2016-18 outbreak. This reduction is assumed to take place no matter whether vaccination strategies are implemented or not. The results are given in Table S24. Comparing to Table 2, Table S24 shows that, with reduction in the contact rate from week 49 of the outbreak, pre-emptive vaccination is cost-effective (<£20,000/QALY) if it increases the seroprevalence among MSM attending SHS by <5.0% over 5 years. As for the scenario with no reduction in contact rate (Table 2), Table S24 shows that adding reactive vaccination to pre-emptive vaccination is cost-effective if pre-emptive vaccination increases the seroprevalence of MSM attending SHS clinics by less than 4% over 5 years.

**Table S24** **Cost-effectiveness analysis under pre-emptive vaccination (PV) and reactive vaccination (RV) alone or in combination when there is a reduction in contact rate during the outbreak.** No reduction in contact rate during the outbreak was assumed. Costs are in GB£ and ICER are in thousands of pounds per QALY.

| Increase in seroprevalence among SHS attendees | Duration of outbreak (days) | Total cases during outbreak | Vaccination costs | Outbreak costs | Productivity losses | Total cost | QALYs | Incremental in comparing current scenario to previous scenario | | | Incremental in comparing current scenario to baseline scenario | | | Incremental - comparing the combination of RV and PV to PV alone with same coverage | | |
| --- | --- | --- | --- | --- | --- | --- | --- | --- | --- | --- | --- | --- | --- | --- | --- | --- |
|  |  |  |  |  |  |  |  | Costs | QALYs | ICER | costs | QALYs | ICER | costs | QALYs | ICER |
| **Pre-emptive vaccination alone** **without control measures taken during the outbreak, with the outbreak cost only including costs due to Clinical Case Management** | | | | | | | | | | | | | | | | |
| 0(baseline) | 3559 | 18477 | 0 | 37861462 | 2523772 | 40385234 | 4123199 |  |  |  |  |  |  |  |  |  |
| 0.01 | 3554 | 4717 | 1902388 | 10858784 | 897489 | 13658661 | 4123734 | -26726573 | 535.2 | CS | -26726572 | 535 | CS |  |  |  |
| 0.02 | 3552 | 2896 | 2853582 | 7174023 | 615813 | 10643418 | 4123808 | -3015243 | 73.6 | CS | -29741815 | 608 | CS |  |  |  |
| 0.03 | 1451 | 2012 | 3614538 | 4828380 | 460392 | 8903310 | 4123846 | -1740108 | 38.4 | CS | -31481923 | 647 | CS |  |  |  |
| 0.04 | 1162 | 1501 | 4565732 | 3646580 | 352284 | 8564596 | 4123869 | -338714 | 22.9 | CS | -31820637 | 670 | CS |  |  |  |
| 0.05 | 982 | 1157 | 5516926 | 2852813 | 283441 | 8653180 | 4123885 | 88584 | 15.5 | 6 | -31732053 | 685 | CS |  |  |  |
| 0.06 | 854 | 917 | 6468120 | 2294127 | 246676 | 9008923 | 4123896 | 355743 | 10.9 | 33 | -31376310 | 696 | CS |  |  |  |
| 0.07 | 757 | 745 | 7609553 | 1893409 | 220546 | 9723507 | 4123903 | 714585 | 7.8 | 92 | -30661726 | 704 | CS |  |  |  |
| 0.08 | 680 | 617 | 8560747 | 1591764 | 200150 | 10352661 | 4123909 | 629154 | 5.8 | 108 | -30032572 | 710 | CS |  |  |  |
| 0.09 | 617 | 521 | 9702180 | 1367808 | 183509 | 11253497 | 4123914 | 900836 | 4.4 | 205 | -29131736 | 714 | CS |  |  |  |
| 0.1 | 565 | 446 | 10843613 | 1186146 | 169538 | 12199297 | 4123917 | 945800 | 3.4 | 278 | -28185936 | 717 | CS |  |  |  |
| **Reactive vaccination alone or with pre-emptive vaccination, with the outbreak cost including costs due to Clinical Case Management and Public Health Response** | | | | | | | | | | | | | | | | |
| RV alone | 956 | 2678 | 0 | 9930766 | 783582 | 10714348 | 4123815 |  |  |  | -29670885 | 617 | CS | -29670885 | 616 | CS |
| 0.01+RV | 934 | 1781 | 1902388 | 6720182 | 479920 | 9102489 | 4123856 | -1611858 | 40.765 | CS | -31282744 | 657 | CS | -4556171 | 122 | CS |
| 0.02+RV | 903 | 1498 | 2853582 | 5703213 | 395646 | 8952441 | 4123869 | -150048 | 12.842 | CS | -31432792 | 670 | CS | -1690977 | 61 | CS |
| 0.03+RV | 861 | 1257 | 3614538 | 4830235 | 334302 | 8779076 | 4123880 | -173365 | 10.914 | CS | -31606157 | 681 | CS | -124234 | 34 | CS |
| 0.04+RV | 812 | 1053 | 4565732 | 4086273 | 290195 | 8942200 | 4123889 | 163124 | 9.272 | 18 | -31443033 | 690 | CS | 377604 | 20 | 19 |
| 0.05+RV | 760 | 884 | 5516926 | 3469037 | 257095 | 9243058 | 4123897 | 300857 | 7.659 | 39 | -31142175 | 698 | CS | 589878 | 12 | 48 |
| 0.06+RV | 708 | 747 | 6468120 | 2962388 | 231231 | 9661739 | 4123903 | 418680 | 6.246 | 67 | -30723494 | 704 | CS | 652816 | 7.7 | 84 |
| 0.07+RV | 658 | 636 | 7609553 | 2549454 | 210315 | 10369322 | 4123908 | 707582 | 5.035 | 141 | -30015911 | 709 | CS | 645814 | 5.0 | 129 |
| 0.08+RV | 611 | 546 | 8560747 | 2211740 | 192906 | 10965393 | 4123912 | 596071 | 4.108 | 145 | -29419840 | 713 | CS | 612732 | 3.3 | 187 |
| 0.09+RV | 568 | 473 | 9702180 | 1940789 | 178159 | 11821128 | 4123915 | 855734 | 3.313 | 258 | -28564105 | 717 | CS | 567631 | 2.2 | 259 |
| 0.1+RV | 530 | 414 | 10843613 | 1715920 | 165470 | 12725003 | 4123918 | 903874 | 2.706 | 334 | -27660230 | 719 | CS | 525706 | 1.5 | 352 |

**SI.4.13 Effect of variation in utility weights on the cost-effectiveness of vaccination strategies**

In the main text, the following utility weights are used to calculate QALYs: 0.83 for asymptomatic, 0.64 for symptomatic, 0.26 for fulminant, 0.73 for post liver transplant cases, and 0.90 for other health states. To test whether different choices of utility weight can change our conclusion about the cost-effectiveness of vaccination strategies, we further consider another two utility weights:

1. increasing all weights by 10% in the absolute terms, that is, 0.93 for asymptomatic, 0.74 for symptomatic, 0.36 for fulminant, 0.83 for post liver transplant cases, and 1.00 for other health states.
2. decreasing weights by 10% in the absolute terms for HAV related states while maintaining the weight for other health states, that is, 0.73 for asymptomatic, 0.54 for symptomatic, 0.16 for fulminant, 0.63 for post liver transplant cases, and 0.90 for other health states.

As the changes in the utility weights only change the values of QALYs while other variables such as outbreak duration and size and the total cost remain the same as in Table 2, for simplicity, only Total Cost, QALYs and ICER will be listed here for comparison of the two choices with the utility weight used in the main text. The results listed in Table S25 show that the QALYs saved for different vaccination scenarios increase by over 38% when the baseline utility weights for each HAV-related health state are reduced by 10% (but unchanged for the non HAV infected health states) as in choice b). Conversely, increasing all utility weights by 10% as in setup a) changes estimates of the QALYs saved by <0.02%. Under these two different setups of utility weight, the conclusion about the effectiveness of vaccination strategies remain the same as in Table 2. That is, the main conclusion in the main text is robust.

**Table S25** **Effect of different utility weight assumptions on the costs, QALYs saved and cost-effectiveness of pre-emptive vaccination (PV) and reactive vaccination (RV) alone or in combination.** No reduction in contact rate during the outbreak was assumed. Costs are in thousands of GB£ and ICER are in thousands of pounds per QALY.

| **Increase in seroprevalence among SHS attendees** | **Total cost** | **QALYs** | **Incremental - comparing current scenario to previous scenario** | | | **Incremental - comparing current scenario to baseline scenario** | | | **Incremental - comparing the combination of RV and PV to PV alone with same coverage** | | |
| --- | --- | --- | --- | --- | --- | --- | --- | --- | --- | --- | --- |
|  |  |  | **Costs** | **QALYs** | **ICER** | **Costs** | **QALYs** | **ICER** | **Costs** | **QALYs** | **ICER** |
| **Pre-emptive vaccination alone** without control measures taken during the outbreak, with the outbreak cost only including costs due to Clinical Case Management | | | | | | | | | | | |
| Utility weight setup used in the main text | | | | | | | | | | | |
| 0(baseline) | 119,509 | 4,121,628 |  |  |  |  |  |  |  |  |  |
| 0.01 | 96,006 | 4,122,076 | -23,503 | 448 | CS | -23,503 | 448 | CS |  |  |  |
| 0.05 | 85,246 | 4,122,362 | -10,759 | 286 | CS | -34,262 | 734 | CS |  |  |  |
| 0.06 | 82,577 | 4,122,488 | -2,670 | 126 | CS | -36,932 | 859 | CS |  |  |  |
| 0.07 | 58,437 | 4,123,111 | -24,140 | 623 | CS | -61,071 | 1483 | CS |  |  |  |
| 0.08 | 16,547 | 4,123,818 | -41,890 | 707 | CS | -102,962 | 2190 | CS |  |  |  |
| 0.09 | 11,149 | 4,123,911 | -5,397 | 93 | CS | -108,359 | 2283 | CS |  |  |  |
| 0.1 | 12,041 | 4,123,916 | 892 | 5 | 184 | -107,467 | 2287 | CS |  |  |  |
| Utility weight setup a) | | | | | | | | | | | |
| 0(baseline) | 119,509 | 4,579,844 |  |  |  |  |  |  |  |  |  |
| 0.01 | 96,006 | 4,580,292 | -23,503 | 448 | CS | -23,503 | 448 | CS |  |  |  |
| 0.05 | 85,246 | 4,580,578 | -10,759 | 286 | CS | -34,262 | 734 | CS |  |  |  |
| 0.06 | 82,577 | 4,580,703 | -2,670 | 126 | CS | -36,932 | 859 | CS |  |  |  |
| 0.07 | 58,437 | 4,581,327 | -24,140 | 623 | CS | -61,071 | 1483 | CS |  |  |  |
| 0.08 | 16,547 | 4,582,034 | -41,890 | 707 | CS | -102,962 | 2190 | CS |  |  |  |
| 0.09 | 11,149 | 4,582,127 | -5,397 | 93 | CS | -108,359 | 2283 | CS |  |  |  |
| 0.1 | 12,041 | 4,582,132 | 892 | 5 | 184 | -107,467 | 2287 | CS |  |  |  |
| Utility weight setup b) | | | | | | | | | | | |
| 0(baseline) | 119,509 | 4,120,733 |  |  |  |  |  |  |  |  |  |
| 0.01 | 96,006 | 4,121,355 | -23,503 | 622 | CS | -23,503 | 622 | CS |  |  |  |
| 0.05 | 85,246 | 4,121,751 | -10,759 | 396 | CS | -34,262 | 1018 | CS |  |  |  |
| 0.06 | 82,577 | 4,121,926 | -2,670 | 174 | CS | -36,932 | 1193 | CS |  |  |  |
| 0.07 | 58,437 | 4,122,790 | -24,140 | 865 | CS | -61,071 | 2057 | CS |  |  |  |
| 0.08 | 16,547 | 4,123,772 | -41,890 | 981 | CS | -102,962 | 3039 | CS |  |  |  |
| 0.09 | 11,149 | 4,123,901 | -5,397 | 129 | CS | -108,359 | 3168 | CS |  |  |  |
| 0.1 | 12,041 | 4,123,908 | 892 | 7 | 133 | -107,467 | 3175 | CS |  |  |  |
| **Reactive vaccination alone or with pre-emptive vaccination**, with the outbreak cost including costs due to Clinical Case Management and Public Health Response | | | | | | | | | | | |
| Utility weight setup used in the main text | | | | | | | | | | | |
| RV alone | 14,160 | 4,123,767 |  |  |  | -105,348 | 2139 | CS | -105,348 | 2,138 | CS |
| 0.01+RV | 12,729 | 4,123,808 | -1,431 | 41 | CS | -106,779 | 2180 | CS | -83,276 | 1,732 | CS |
| 0.05+RV | 12,669 | 4,123,858 | -60 | 49 | CS | -106,840 | 2229 | CS | -72,577 | 1,495 | CS |
| 0.06+RV | 11,720 | 4,123,880 | -949 | 22 | CS | -107,788 | 2252 | CS | -70,857 | 1,392 | CS |
| 0.07+RV | 10,488 | 4,123,904 | -1,232 | 24 | CS | -109,021 | 2276 | CS | -47,949 | 793 | CS |
| 0.08+RV | 10,925 | 4,123,910 | 437 | 6 | 71 | -108,583 | 2282 | CS | -5,621 | 92 | CS |
| 0.09+RV | 11,702 | 4,123,915 | 777 | 4 | 178 | -107,807 | 2286 | CS | 553 | 4 | 153 |
| 0.1+RV | 12,571 | 4,123,918 | 869 | 3 | 268 | -106,937 | 2289 | CS | 530 | 2 | 264 |
| Utility weight setup a) | | | | | | | | | | | |
| RV alone | 14,160 | 4581983 |  |  |  | -105,348 | 2139 | CS | -105,348 | 2,138 | CS |
| 0.01+RV | 12,729 | 4582024 | -1,431 | 41 | CS | -106,779 | 2180 | CS | -83,276 | 1,732 | CS |
| 0.05+RV | 12,669 | 4582073 | -60 | 49 | CS | -106,840 | 2229 | CS | -72,577 | 1,495 | CS |
| 0.06+RV | 11,720 | 4582096 | -949 | 22 | CS | -107,788 | 2252 | CS | -70,857 | 1,392 | CS |
| 0.07+RV | 10,488 | 4582120 | -1,232 | 24 | CS | -109,021 | 2276 | CS | -47,949 | 793 | CS |
| 0.08+RV | 10,925 | 4582126 | 437 | 6 | 71 | -108,583 | 2282 | CS | -5,621 | 92 | CS |
| 0.09+RV | 11,702 | 4582130 | 777 | 4 | 178 | -107,807 | 2286 | CS | 553 | 4 | 153 |
| 0.1+RV | 12,571 | 4582134 | 869 | 3 | 268 | -106,937 | 2289 | CS | 530 | 2 | 264 |
| Utility weight setup b) | | | | | | | | | | | |
| RV alone | 14,160 | 4,123,702 |  |  |  | -105,348 | 2969 | CS | -105,348 | 2,969 | CS |
| 0.01+RV | 12,729 | 4,123,759 | -1,431 | 57 | CS | -106,779 | 3026 | CS | -83,276 | 2,404 | CS |
| 0.05+RV | 12,669 | 4,123,827 | -60 | 68 | CS | -106,840 | 3125 | CS | -72,577 | 2,075 | CS |
| 0.06+RV | 11,720 | 4,123,858 | -949 | 31 | CS | -107,788 | 3158 | CS | -70,857 | 1,932 | CS |
| 0.07+RV | 10,488 | 4,123,892 | -1,232 | 34 | CS | -109,021 | 3167 | CS | -47,949 | 1,101 | CS |
| 0.08+RV | 10,925 | 4,123,900 | 437 | 9 | 51 | -108,583 | 3173 | CS | -5,621 | 128 | CS |
| 0.09+RV | 11,702 | 4,123,906 | 777 | 6 | 128 | -107,807 | 3177 | CS | 553 | 5 | 110 |
| 0.1+RV | 12,571 | 4,123,911 | 869 | 5 | 193 | -106,937 |  | CS | 530 | 3 | 190 |

CS denotes cost saving where the option is cheaper compared to the comparator and QALYs are gained. The underlined rows show the maximum levels of pre-emptive vaccination rate that are CS alone and in combination with reactive vaccination, respectively.

**Other tables**

**Table S6** **Costs of interventions to control the Hepatitis A within MSM living in England- based on cost estimates and outcome data from the 2016/18 outbreak.** M is the number of cases in the 2023 outbreak.

| **Component** | **Unit cost(£)** | **Number of units** | **Total cost (2017£)** | **Ref notes** |
| --- | --- | --- | --- | --- |
| ***Clinical case management (CCM)*** | | | |  |
| Accident and Emergency attendance (AE) | U_AE_=147.80 | X_AE_=B(M,p_AE_) | C_AE_=U_AE_×X_AE_ | *p*_AE_= 58% or 127/220 |
| Hospital admission (HA) | U_HA_=3,184.84 | X_HA_=B(M,p_HA_) | C_HA_=U_HA_×X_HA_ | *p*_HA_= 63.7% or 452/(710-87) |
| GP consultation (GP) | U_GP_=37.00 | X_GP_=B(M,p_GP_) | C_GP_=U_GP_×X_GP_ | p_GP_=63.3% or 416/(796-140) |
| Fulminant hepatitis leading to liver transplant (LT) | U_LT_= 69637.00 | X_LT_=B(M,p_LT_) | C_LT_=U_LT_×X_LT_ | p_LT_= 1/796 |
| Cost__CCM_ = C_AE_+C_HA_+C_GP_+C_LT_ = U_AE_×X_AE_  + U_HA_×X_HA_  + U_GP_×X_GP_  + U_LT_×X_LT_  = U_AE_× B(M,p_AE_) + U_HA_× B(M,p_HA_) + U_GP_× B(M,p_GP_) + U_LT_× B(M,p_LT_) | | | | |
|  |  |  |  |  |
| ***Public health response (PHR)*** | | | | |
| Vaccination of contacts in primary care (VPC) | U_VpC_= £30.00 (£20 for 1 dose of vaccination, £10 for administering vaccine [3,17] | Y_VpC_=M×κ^PC^ | C_VC_=U_VC_×Y_VC_ | The ratio of vaccines per case κ^PC^=3.31(2.96,3.67) |
| Extra vaccinations ordered during outbreak by SHS clinics (VSHS) | U_VSHS_=£30.00 | Y_VSHS_=M×κ^SHS^ | C_VSHS_=U_VSHS_×Y_VSHS_ | the ratio of vaccines ordered per case κ^SHS^ =34.41 or (27,391÷796) |
| Routine management of outbreak cases (RM) | U_RM_=£200.29 | Y_RM_=M | C_RM_=U_RM_×Y_RM_ | Assume same unit cost per case |
| Extra management of exceptional outbreak cases  (EM) | U_EM_ =£176.84 | Y_EM_=B(M,p_EM_) | C_EM_=U_EM_×Y_EM_ | the ratio of exceptional cases p_EM_= 192/796 |
| Ordinary incidents during outbreak (OI) | U_OI_ =118.36 | Y_OI_=B(M,p_OI_) | C_OI_=U_OI_×Y_OI_ | the ratio of outbreak incidents per case p_OI_= 44/796 |
| Extraordinary incidents during outbreak (EI) | U_EI_ =8,246.32 | Y_EI_=B(M,p_EI_) | C_EI_=U_EI_×Y_EI_ | the ratio of extraordinary outbreaks incidents per case p_EI_= 1/796 |
| Changes to Standard operating procedures (CS) | U_CS_= £85.03 or 0 if M< 30 cases over 3 months | Y_CS_=B(M,p_CS_) | C_CS_=U_CS_×Y_CS_ | the ratio per case p_CS_= 27/796 |
| Data collection from SHS | U_DC_ = £26,538.65 | Y_DC_=D_OTB_/13 | C_DC_=U_DC_×Y_DC_ | Continues as long as outbreak (D_OTB_) – this was a little over 1 year (assume 13 months) for current outbreak. Divide down to shorter time frame for shorter epidemics. |
| Health promotion  (HP) | U_HP_=£13,738.59 | Y_HP_=M/796 | C_HP_=U_HP_×Y_HP_ | Proportion to number of cases in outbreaks |
| **Cost__PHR_ =** C_VpC_ +C_VSHS_ +C_RM_ +C_EM_ +C_OI_ +C_EI_ +C_CS_ +C_DC_+C_HP_  = U_VpC_×Y_VpC_ +U_VSHS_×Y_VSHS_ +U_RM_×Y_RM_ +U_EM_×Y_EM_ +U_OI_×Y_OI_ +U_EI_×Y_EI_ +U_CS_×Y_CS_ + U_DC_×Y_DC_ +U_HP_×Y_HP_  = U_VpC_× M×κ^PC^ +U_VSHS_× M×κ^SHS^ +U_RM_×M +U_EM_× B(M,p_EM_) +U_OI_× B(M,p_OI_) +U_EI_× B(M,p_EI_) +U_CS_× B(M,p_CS_) + U_DC_× D_OTB_/13+U_HP_×M/796 | | | | |
|  |  |  |  |  |
| ***Coordination & training (CT)*** | | | | |
| National outbreak meetings (NOM) | U_NOM_=£4,812.68 | Y_NOM_  =D_OTB_×(13/13) | C_NOM_=U_NOM_×Y_NOM_ | 13/13 meetings for each month of outbreak. |
| Local Regional outbreak meetings (ROM) | U_ROM_ =£1,221.64 | Y_ROM_  =max(2, D_OTB_×(38/13)) | C_ROM_=U_ROM_×Y_ROM_ | 38/13 meetings per month of outbreak with minimum of 2 meetings |
| HPT coordination meetings (HPTM) | U_HPTM_=£1,317.98 | Y_HPTM_  =if(M>30 cases in 3 months,1,0) | C_HPTM_=U_HPTM_×Y_HPTM_ | one meeting occurs if there is a outbreak |
| London Commissioner meetings (LCM) | U_LCM_=£1661.83 | Y_LCM_=2 | C_LCM_=U_LCM_×Y_LCM_ | occur irrespective of number of cases if there is a outbreak |
| Preparation of letters for communication about outbreak (WC) | U_WC_=£469.30 | Y_WC_=5 | C_WC_=U_WC_×Y_WC_ | Assume fixed cost if there is an outbreak |
| On-going communication to stakeholders during outbreak (discussion and sending letters) (com) | U_COM_=£14,808.02 | Y_COM_=M/796 | C_COM_=U_COM_×Y_COM_ | proportion to number of cases in outbreak |
| Training on taking sexual history (SXT) | U_SXT_ =£3523.04 | Y_SXT_=5 (0,5,10) | C_SXT_=U_SXT_×Y_SXT_ | the same total cost if there is any outbreak |
| Training on use of Sexual health history taking training App (LSXT) | ULSXT=£4,403.81 | YLSXT=1 | (CLSXT) | Assume a fixed constant cost if there is an outbreak |
| Cost__CT_ = C_NOM_ +C_ROM_ +C_HPTM_ +C_LCM_ +C_COM_ +C_LSXT_ + C_SXT_+ C_WC_  = U_NOM_×Y_NOM_ +U_ROM_×Y_ROM_ +U_HPTM_×Y_HPTM_ +U_LCM_×Y_LCM_ +U_COM_×Y_COM_ +C_LSXT_  = U_NOM_×D_Outbreak_×(13/25) +U_ROM_× max(2,D_Outbreak_×(19/13)) +U_HPTM_× if(30^+^cases in 3 months,1,0)) +U_LCM_×Y_LCM_ +U_COM_×M/796 +C_LSXT_ +U_SXT_×Y_SXT_(D_OTB_,M)+ U_WC_×Y_WC_(D_OTB_,M) | | | | |

**References**

1. Presanis, A.M., et al., *Trends in undiagnosed HIV prevalence in England and implications for eliminating HIV transmission by 2030: an evidence synthesis model.* Lancet Public Health, 2021. **6**(10): p. e739-e751.

2. MacGregor, L., et al., *Behavioural, not biological, factors drive the HCV epidemic among HIV-positive MSM: HCV and HIV modelling analysis including HCV treatment-as-prevention impact.* Int J Epidemiol, 2017. **46**(5): p. 1582-1592.

3. Plunkett, J., et al., *Hepatitis A outbreak among men who have sex with men (MSM) in England, 2016-2018: The contribution of past and current vaccination policy and practice.* Vaccine X, 2019. **1**: p. 100014.

4. Zhang, X.S. and A. Charlett, *Bayesian modelling of a hepatitis A outbreak in men who have sex with men in Sydney, Australia, 1991/1992.* Epidemiol Infect, 2019. **147**: p. e226.

5. Dankwa, E.A., et al., *Estimating vaccination threshold and impact in the 2017-2019 hepatitis A virus outbreak among persons experiencing homelessness or who use drugs in Louisville, Kentucky, United states.* Vaccine, 2021. **39(49)**: p. 7182-7190.

6. Koff, R.S., *Clinical manifestations and diagnosis of hepatitis A virus infection.* Vaccine, 1992. **10 Suppl 1**: p. S15-7.

7. Connor, B.A., *Hepatitis A vaccine in the last-minute traveler.* Am J Med, 2005. **118 Suppl 10A**: p. 58S-62S.

8. Anderson, R. and R.M. May, *Infectious diseases of humans*. 1992: Oxford University Press.

9. Vynnycky, E. and R.G. White, *An Introduction to Infectious Disease Modelling*. 2010, New York: Oxford University Press.

10. Birrell, P.J., et al., *Reconstructing a spatially heterogeneous epidemic: Characterising the geographic spread of 2009 A/H1N1pdm infection in England.* Sci Rep, 2016. **6**: p. 29004.

11. Fitzpatrick, C., et al., *Hepatitis A susceptibility in newly attending men who have sex with men to an urban sexual health centre.* Int J STD AIDS, 2021. **32**(3): p. 276-279.

12. Bhagey, A., et al., *High prevalence of anti-hepatitis A IgG in a cohort of UK HIV-negative men who have sex with men: implications for local hepatitis A vaccine policy.* Int J STD AIDS, 2018. **29**(10): p. 1007-1010.

13. Dhankhar, P., et al., *Public Health Impact and Cost-Effectiveness of Hepatitis A Vaccination in the United States: A Disease Transmission Dynamic Modeling Approach.* Value Health, 2015. **18**(4): p. 358-67.

14. Lin, Y., et al., *[Cost-effectiveness and affordability of strategy for preventing mother-to-child transmission of hepatitis B in China].* Zhonghua Liu Xing Bing Xue Za Zhi, 2017. **38**(7): p. 852-859.

15. Office of National Statistics. *Employee earnings in the UK: 2019 (*[*https://www.ons.gov.uk/employmentandlabourmarket/peopleinwork/earningsandworkinghours/bulletins/annualsurveyofhoursandearnings/2019*](https://www.ons.gov.uk/employmentandlabourmarket/peopleinwork/earningsandworkinghours/bulletins/annualsurveyofhoursandearnings/2019)*)*. 2019 16th November 2021].

16. Office of National Statistics. *Employment in the UK: May 2020 (*[*https://www.ons.gov.uk/employmentandlabourmarket/peopleinwork/employmentandemployeetypes/bulletins/employmentintheuk/may2020*](https://www.ons.gov.uk/employmentandlabourmarket/peopleinwork/employmentandemployeetypes/bulletins/employmentintheuk/may2020)*)*. 2020 16th November 2021].

17. Edelstein, M., et al., *Implementation and evaluation of the human papillomavirus (HPV) vaccination pilot for men who have sex with men (MSM), England, April 2016 to March 2017.* Euro Surveill, 2019. **24**(8).
